# Helixer: cross-species gene annotation of large eukaryotic genomes using deep learning

**DOI:** 10.1093/bioinformatics/btaa1044

**Published:** 2020-12-16

**Authors:** Felix Stiehler, Marvin Steinborn, Stephan Scholz, Daniela Dey, Andreas P M Weber, Alisandra K Denton

**Affiliations:** btaa1044-aff1 Institue of Plant Biochemistry, Faculty of Mathematics and Natural Sciences, Heinrich-Heine-University, Dusseldorf 40225, Germany; btaa1044-aff2 Institute of Human Genetics, Medical Faculty, RWTH Aachen University, Aachen 52062, Germany

## Abstract

**Motivation:**

Current state-of-the-art tools for the *de novo* annotation of genes in eukaryotic genomes have to be specifically fitted for each species and still often produce annotations that can be improved much further. The fundamental algorithmic architecture for these tools has remained largely unchanged for about two decades, limiting learning capabilities. Here, we set out to improve the cross-species annotation of genes from DNA sequence alone with the help of deep learning. The goal is to eliminate the dependency on a closely related gene model while also improving the predictive quality in general with a fundamentally new architecture.

**Results:**

We present Helixer, a framework for the development and usage of a cross-species deep learning model that improves significantly on performance and generalizability when compared to more traditional methods. We evaluate our approach by building a single vertebrate model for the base-wise annotation of 186 animal genomes and a separate land plant model for 51 plant genomes. Our predictions are shown to be much less sensitive to the length of the genome than those of a current state-of-the-art tool. We also present two novel post-processing techniques that each worked to further strengthen our annotations and show in-depth results of an RNA-Seq based comparison of our predictions. Our method does not yet produce comprehensive gene models but rather outputs base pair wise probabilities.

**Availability and implementation:**

The source code of this work is available at https://github.com/weberlab-hhu/Helixer under the GNU General Public License v3.0. The trained models are available at https://doi.org/10.5281/zenodo.3974409

**Supplementary information:**

Supplementary data are available at *Bioinformatics* online.

## 1 Introduction

Annotating genes is an integral part of genomic DNA sequence analysis, with many downstream taks dependent on annotation quality. Gene annotation can be performed at different levels of precision, from simple coding—non-coding classification to detailed structural labeling. Because of the sheer size of genomes alone, manual gene annotation is generally intractable. Instead, researchers can use pipelines such as Maker (Cantarel *et al.*, 2007), PASA (Haas *et al.*, 2003) or those offered by genomic database providers like NCBI (Thibaud-Nissen *et al.*, 2013) and Ensembl (Aken *et al.*, 2016). These pipelines integrate experimental data (from e.g. RNA-seq or proteogenomics) with homologous sequences in the database and ab-initio gene predictions. The latter is an attractive approach, because it is cheap and fast. State-of-the-art performance is achieved by higher order hidden markov models (HMMs), such as Genscan (Burge and Karlin, 1997), AUGUSTUS (Stanke and Waack, 2003) or SNAP (Johnson *et al.*, 2008). Their accuracy, however, leaves room for improvement. By encoding possible states and transitions in a probalistic model, designers of HMMs assume structure in the sequence that may limit its predictive power. In practice HMMs have trouble generalizing across species and the actual learning of sequence motifs is limited to very short sequences that indicate state transitions.

In the last decade, deep neural networks (DNN) have been applied with great success in many areas of statistical modeling, including biology (Ching *et al.*, 2018). For sequence data, such as DNA, speech or text, a special kind of recurrent neural network (RNN) called long-short term memory (LSTM) (Hochreiter and Schmidhuber, 1997) is an established building block for many different architectures. LSTM units can also be used to process sequential input starting from both ends, forming a bidirectional LSTM (BLSTM). It has also been shown that HMMs can be successfully combined with DNNs (Liu *et al.*, 2016a,b).

For the purpose of gene annotation, RNNs have already shown promising results. (Choudhary, 2017) carries out preliminary explorations on the potential of BLSTMs for cross species gene prediction and trains his model on human genes to test it later on two more species. DeepAnnotator (Amin *et al.*, 2018) uses BLSTMs for gene finding in prokaryotes. Gene prediction in prokaryotes is considered more amenable than in eukaryotes, as genes in prokaryotes are proportionately more frequent in the genome, feature simpler control structures and do not use splicing (Wang et al., 2004). DanQ (Quang and Xie, 2016) proposes the use of a BLSTM after a convolutional neural net (CNN) to find detailed motifs in the human genome. DeePromoter (Oubounyt *et al.*, 2019) trains a similar architecture for the recognition of promoter regions. Recently, several groups (Jaganathan *et al.*, 2019; Wang et al., 2019) successfully used CNNs to find splicing sites.

In this work, we present Helixer, a novel prototype software for training and utilizing a general purpose DNN for the ab-initio cross-species base-wise gene annotation of large eukaryotic genomes using only DNA sequence as input. Our model is trained to differentiate between four regions: Intergenic, Untranslated (UTR), Coding (CDS) and Intron. We demonstrate the effectiveness of this approach by training two models, one each for the annotation of a large set of genomes from the domains metazoa and viridiplantae, respectively, which we will call *animal* and *plant* from now on. We worked with the full data of 192 animal genomes and 60 plant genomes. These datasets were rich in vertebrates and land plants, respectively, which is reflected in a much better average model performance on those phylogenetic groups. We will thus call our models *vertebrate model* and *land plant model*.

Both the ability to generalize across species as well as the scope of the evaluation represent cutting edge progress in this field. The source code and all input data are publicly available.

## 2 Materials and methods

### 2.1 Datasets

The foundation of our work are 192 animal and 60 plant genomes. The data of each genome consists of the latest publicly available genomic assembly in form of a FASTA file and the latest annotation in the GFF format. We used one genome for each animal species in EnsemblMetazoa 45 (Howe *et al.*, 2020; Supplementary Table S1) as well as all non-embargoed plant species from the JGI Phytosome 13 database (Goodstein *et al.*, 2012; Supplementary Table S2); The exact genomes are listed in the aforementioned tables. Data was downloaded on the October 15, 2019 and March 29, 2019 from EnsembleMetazoa and Phytozome respectively. Both data groups were used completely separately throughout.

**Fig. 1.**
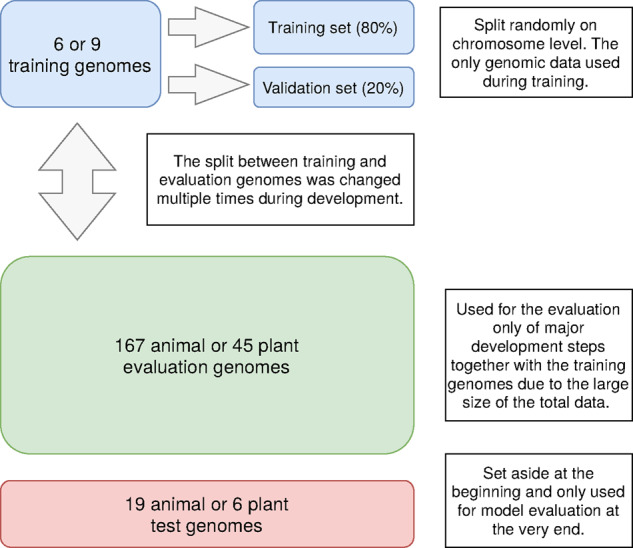
Division of the total set of genomes in both the animal and plant case

Directly after obtaining the data, we split off 19 or 6 *test genomes* from either group of genomes and set those aside for the final evaluation at the very end of the development process (Fig. 1). These genomes were chosen to be of decent quality and diversity based on collected metadata (see Supplementary Section S1 and Supplementary Dataset S1) while also representing a broad phylogenetic spread.

The remaining genomes were seperated into a set of *training genomes*, which were used to actually train the neural networks, and *evaluation genomes*, which provided crucial feedback on the generalization capabilities during development (Fig. 1). The exact division of which genomes we train with and which are just used for evaluation was changed multiple times and was found to be crucial for model performance.

To select the training genomes, we had to balance multiple conflicting trade-offs. On the one hand, we want to train with as much data as possible, but on the other not all data has sufficient quality to enable a powerful generalization. It is also very desirable to have diverse training genomes with a broad phylogenetic spread, a variety of genome sizes and average gene lengths as the model is increasingly unlikely to generalize well beyond the borders given by the training data. However, it also may be difficult for the model to learn and generalize if the genomic patterns inside the data are too different from each other. Practically, it was important for us to limit the size of our training genomes set to be small enough that we could get experimental results within a couple of days and thereby be able to test many different data and model configurations.

We used an iterative approach to effectively select a proper set of training genomes. We started with 3–4 genomes that were expected to be of the highest quality and then evaluated the performance of the resulting model on all training and evaluation genomes individually. There, we looked for candidates to add to the set of training genomes (which were evaluation genomes and had high Genic F1) or to remove from it (which were training genomes and had a rather low Genic F1). To make these decisions, we also factored in information like genomic metadata (Supplementary Section S1 and Supplementary Dataset S1) and the phylogenetic spread our new set of training genomes would have. This process was repeated multiple times alternatingly with the model search as the decisions about the model architecture and the training data tend to depend on each other. We stopped this process when we saw no more room for substantial improvement given our computational constraints.

We report only the generalization performance on the combined set of evaluation and test genomes as the evaluation genomes are by far the largest set, and there was not a noticeable difference in performance between those two groups (see Supplementary Section S2 for a performance breakdown by each species).

Once a set of training genomes was selected, we further split the sequences therein into a *training and validation set*. This split of the training genomes was done to get a quicker sense of the generalization capabilities and was used after each training epoch. We split off the validation set by selecting 20% of the FASTA sequences above and below the N90 of each training genome at random, ensuring a proper distribution of large and short sequences in both sets and a split on the chromosome level. Evaluation of the annotations on all training and evaluation genomes was done regularly after a promising model candidate was found based on its validation set performance. Ultimately we used the cross-species performance on all evaluation genomes as the decisive measure of model quality.

### 2.2 Data pre-processing

We first pre-processed and stored the raw genomic information by using GeenuFF (https://github.com/weberlab-hhu/GeenuFF). GeenuFF is a tool for checking and exploring genomic data and annotations, that stores all information inside a SQL database. Training and evaluation-ready data was generated by querying this database and then transforming the returned data into a numerical format suitable for machine learning. The encoding of the genomic sequence was done in line with the IUPAC nucleic acid notation and the structural gene annotation used as labels during training is transformed to a one hot encoding with the four classes *Intergenic*, *UTR*, *CDS* and *Intron*. See Table 1 for a more detailed description of the generated data types. 

**Table 1.** Data arrays generated and used by Helixer

Name	Information
Input	Genomic sequence in the 4-dimensional IUPAC encoding
	(one hot encoding for non-ambiguous bases)
Output	Labels in a 4-dimensional one hot encoding representing
	the classes *Intergenic*, *UTR*, *CDS* and *Intron*
Sample weights	One of {0, 1}; whether there is an error at a base

*Note:* The encoding does not differentiate between introns in coding and non-coding regions.

During data generation, we queried for the transcript with the longest protein of each gene and disregard FASTA sequences that have no structural gene annotation as it is ambiguous whether such sequences contain no genes or were simply not annotated in the reference. GeenuFF checks the genomic annotations for potential errors during import and is able to mark those areas. We used this information to effectively mask those bases during training by using the sample weights described in Table 1. The vast majority of masked bases lie in the intergenic region as the most prevalent error is a missing UTR and GeenuFF marks a potentially large intergenic region for it. Table 2 shows statistics about the masking.

**Table 2.** Data group statistics for all data

	Animals	Plants
Average genome size in Gbp	2.936 (±1.562)	0.787 (±0.995)
Average gene length	31 223 (±13 974)	3368 (±1510)
Geenuff error rate	0.311 (±0.129)	0.351 (±0.249)
Fraction of class Intergenic	0.777 (±0.042)	0.799 (±0.089)
Fraction of class UTR	0.006 (±0.006)	0.017 (±0.016)
Fraction of class CDS	0.016 (±0.014)	0.085 (±0.071)
Fraction of class Intron	0.201 (±0.035)	0.099 (±0.057)

*Note:* Values are averages of the individual values of each genome in a data group. All statistics except the average gene lengths exclude FASTA sequences without a gene and any 20 000 bp subsequences that were masked as completely erroneous. Each strand of DNA was counted separately. The gene length was determined by the length of the pre-mRNA of the longest protein at each loci. Brackets show the standard deviation.

For the training itself, we divided each continuous genomic sequence into 20 000 bp long subsequences, for which one-hot vectors of the base pairs and annotations are generated and respectively used as input and label for the neural network together with the sample weights. We appended zero padding if the subsequences are shorter than 20 000 bp. If a subsequence is fully marked as erroneous, we excluded it from all analyses.

### 2.3 Metrics

We mainly used two metrics to judge model performance against the references. The *Genic F1* was selected as our primary metric and provides the most comprehensive picture of annotation quality in one number. The *Subgenic F1* is similar to the Genic F1, except that it does not take UTR predictions into account. This was calculated for comparability with AUGUSTUS as further explained in Supplementary Section 2.7.

Both metrics work by first transforming the probalistic output of the model into concrete predictions with an *argmax* operation, as is commonly done in classification. As we are now given the true and predicted class of each base, we calculate the full confusion matrix for all classes. From there, the True Positives (TP), False Positives (FP) and False Negatives (FN) of each considered class are summed up. This means that if, for example, an intronic base is incorrectly labeled as CDS, it would lead to a FP for the CDS class and FN for the intron class. These three values are then combined into precision and recall before the final F1 score is calculated. The formulas 1-6 describe the calculations given the confusion matrix with *C* containing the set of the considered classes.

(1) $$\text{TP}=\sum_{c\in C}TP_{c},$$

(2) $$\text{FP}=\sum_{c\in C}FP_{c},$$

(3) $$\text{FN}=\sum_{c\in C}FN_{c},$$

(4) $$\text{Precision}=\frac{\text{TP}}{\text{TP}+\text{FP}},$$

(5) $$\text{Recall}=\frac{\text{TP}}{\text{TP}+\text{FN}},$$

(6) $$F_{1}=2\cdot \frac{\text{Precision}\cdot \text{Recall}}{\text{Precision}+\text{Recall}}.$$

Neither metric includes the intergenic class, as this class is very abundant and appears to be by far the easiest to predict. (See Table 2 for the class distribution and Supplementary Tables S3 and S4 for a more in-depth report our model performance including the intergenic class). In the case of the Genic F1, this means that effectively only the TP of the intergenic class were disregarded. The metrics essentially provide a weighted mean of the performances in the considered classes, with weights proportional to class frequency.

**Fig. 2.**
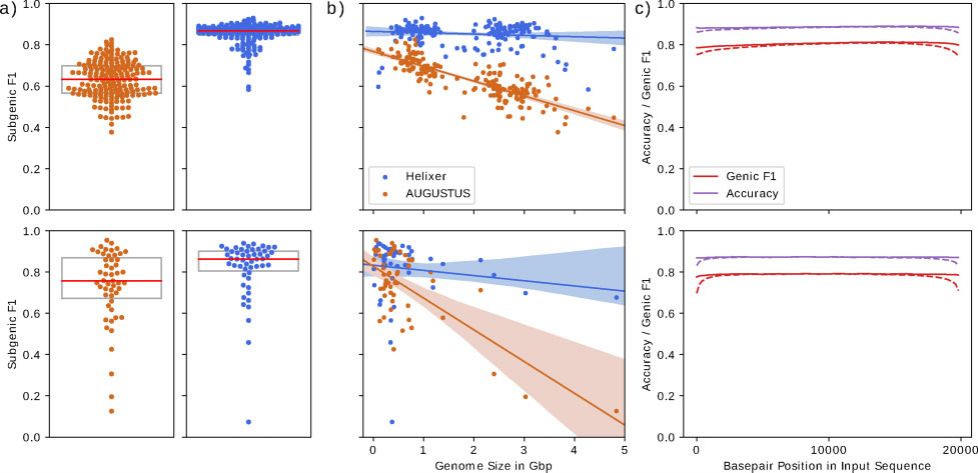
Main overall results and other investigations. The top and bottom rows show the animal and plant data, respectively. Orange dots are for AUGUSTUS and the blue ones are for Helixer (top vertebrate model, bottom land plant model). (**a**) Swarm—and boxplots showing the Subgenic F1 versus the reference scores for all evaluation and test genomes. Each dot represents the prediction performance for one genome. The median prediction scores are shown by the red line. Details of how AUGUSTUS was used are given in Supplementary Section S2.7. (**b**) Scatterplots showing the prediction performances measured in Subgenic F1 versus the reference by genome length. The regression line is shown with a 95% confidence interval. (**c**) The average Genic F1 (in red) and basepair wise accuracy (in purple) versus the reference with respect to the position in the 20 000 basepair long input sequences. The dashed line shows the same without overlapping. Each value is the average performance of a 200 basepair long subsection by one of the models of the final ensemble. See Supplementary Section S10 of for the effects of overlapping in individual species

In Figure 2c, we also report on the overall base pair level accuracy (correctly predicted base pairs/total base pairs) besides the Genic F1.

### 2.4 Model architecture and training

We used a 4-layer deep stacked BLSTM network with 256 units per layer and layer normalization (Ba *et al.*, 2016) between each BLSTM layer to produce the predictions in the form of a base pair wise classification. The model consists of circa 5.4 million parameters and was implemented with the deep learning library Keras (https://keras.io) on top of TensorFlow (Abadi *et al.*, 2016). We tested multiple different architectures before arriving at this model configuration, including convolutional neural networks (CNN) and hybrid architectures. We also used class weights as the class frequencies are both unbalanced and vary greatly between animals and plants. For example, the trade-off between the number of intergenic and intronic bases is quite different in both groups (see Table 2).

A class wise evaluation including the calculation of the Genic F1 score was performed on the validation set after every epoch. The best model from any training run was selected as the model with the highest Genic F1 after either maximum epochs were reached or Genic F1 stopped improving and training was interrupted.

The hyperparameters were optimized by a combination of manual and automatic optimization. Automatic optimization was carried out by using either the TPE algorithm (Bergstra *et al.*, 2011), random search or grid search depending on the situation. We used NNI (https://github.com/microsoft/nni) to facilitate the search. All relevant hyperparameters can be found in Supplementary Tables S6 and S7 or in the Helixer source code repository.

Our final model (Supplementary Figs S5 and S6) was trained with 10 bases of genomic sequence as input during each time step and produces individual predictions for each of those 10 bases simultaneously. This grouping enabled us to train effectively with far longer sequences than usual, as 10 bases can be processed essentially in the time of 1. The tradeoff is, that the data setup is now more complex from the point of view of the neural network. A donor splice site at the beginning of a 10 base pair block now has to result in a fundamentally different set of 40 (10 bases x 4 classes) floating point numbers than a donor splice site at the end.

These four floating point numbers per base are the final output of our model as we are currently not producing a fully coherent gene model in the form of a GFF file.

We also compared our final models to a dilated CNN (dCNN) and a hybrid architecture, that we call DanQ after an existing approach (Quang and Xie, 2016). These were chosen as both dilated CNN and hybrid architectures have been used when working with DNA data as input (Trabelsi et al., 2019). Details of the neural architecture search are given in Supplementary Section S3.

### 2.5 Inference techniques

We also used multiple techniques to improve the prediction quality after the training was done. One very effective way for genomes with larger genes was inputting longer sequences during inference than during training. This was done for all animal genomes except the invertebrates. The input sequences were up to 10 times longer, depending on the phylogenetic group and assembly quality. This also demonstrates the ability of our model to generalize as it is able to make successful prediction on far longer sequences than it has ever seen. The concrete lengths were chosen to keep the typical average gene length roughly proportional to the length of the sequence input. For more implementation details on this see Supplementary Section S9 or the source code.

The final predictions of a single model were constructed by overlapping predictions, which were made from a sliding window and then cropped to a core sequence. This was done to strongly reduce a typical drop in performance of the models toward the beginning and end of each sequence (see Fig. 2c). It also improves the average model performance by providing the model with multiple different starting points. The different overlapping sequences were combined by averaging the individual softmax values of each base. The figures in Supplementary Section S10 show the effect of overlapping for each genome, ordered by N75. We found that overlapping tends to work best if the genomes are not very fragmented and we used it for both animals and plants.

For both the vertebrate and land plant model, a model ensemble with 8 components was used to generate the final predictions for each species. To produce these 8 components, we performed 4 separate training runs and selected two checkpoints each (Supplementary Table S19). First we selected the checkpoint from the epoch with the highest genic F1, which, as it happens, also had either the highest precision or recall. The second checkpoint was selected to complement this, so that checkpoints from the epochs with both the best precision and the best recall were ultimately selected. This was done to increase the diversity of the model ensemble. As with the overlapping, the fusion of the eight individual predictions was done by simply averaging the softmax values of each base pair prediction.

### 2.6 Training and inference times

The training of one vertebrate and one land plant model took on average 9.5 or 5 h per epoch, respectively. The best model performance in terms of Genic F1 was reached in 7–9 epochs with the animal data and 10–13 epochs for the plants. We stopped the training when either there was no improvement in Genic F1 larger than 0.0001 for at least 2 epochs or the training diverged into a situation where a loss of zero was output for only intergenic predictions. This was likely caused by a floating point overflow in the GPU and usually happened as the improvements in model performance appeared to taper off.

We used a single Nvidia GeForce GTX 1080 TI provided by the HPC of the University of Dusseldorf for the training of six of the eight models that make up the final ensembles and a Nvidia GeForce RTX 2080 TI inside a desktop PC with a SSD attached for the other two (with one full GPU per training in each scenario). All times reported here are for the former setup up and training on the latter was roughly 1.5 times faster.

Inference on *Homo sapiens* (circa 6.12Gbp, including padding) took about 8.5 h with overlapping and circa 70 min without. Generating probalistic predictions for *Arabidopsis thaliana* (about 0.22Gbp) finished after close to 7 min. Inference was done on a Nvidia GeForce RTX 2080 TI attached to a regular HDD.

### 2.7 Evaluation of AUGUSTUS

Due to the practical time constraints for retraining and running, we decided to compare our Helixer models only to one existing *de novo* tool, namely the popular gene caller AUGUSTUS (Stanke and Waack, 2003). Typical usage requires retraining AUGUSTUS for each species, with the exception of a few lucky cases where a model for a sufficiently close relative is already available. To scale this for the large plant and animal datasets, we used protein-homology to create an AUGUSTUS training set and therefore could train and evaluate only models without UTRs.

Orthologs of highly conserved generally single-copy genes were identified in each genome using BUSCO (Simão, 2015). The *viridiplantae* set was used for plants and the *metazoa* set for animals. The training genbank files were generated directly by BUSCO, by utilizing the ‘–long’ parameter. For plants, the entire retraining could be performed as above; however, for animals we randomly selected only half the BUSCO-generated training set, which resulted in a training set size and runtime comparable to the plants (about 400 genes and several CPU days per species). For animals the training was carried out with the subsetted training file and using the following scripts provided by AUGUSTUS. An untrained model was setup with ‘new_species.pl’, and the model was fit by running ‘etraining’ before and after the major hyper-parameter optimization with ‘optimize_augustus.pl’. Using the trained model for each species, we ran the main prediction (‘augustus’) with ‘–UTR=off’ and ‘–gff3 = on’. The gffs produced by AUGUSTUS were imported into GeenuFF and exported as HDF5 files in the same manor as the reference, allowing for the direct comparison with both the reference and Helixer predictions. While this method was feasible for some 237 species, it allowed neither training nor prediction of UTR regions with AUGUSTUS, so the metric Subgenic F1, which disregards the class UTR, was used for all comparisons between AUGUSTUS and Helixer (see Supplementary Section S2.3 for more details).

### 2.8 Evaluation against independent RNAseq data

The reference annotations were created with existing tools and largely with a pipeline incorporating *de novo* gene predictions with RNAseq and homology data. As the references, like any data, are expected to contain errors we chose to use RNAseq data for an independent evaluation. In plants there is the additional concern that the references may share biases with AUGUSTUS or HMMs as AUGUSTUS was used as the *de novo* gene caller for the reference of many plant species and other HMM-based tools for many more. The Ensemble animal dataset by and large used the Ensembl annotation pipeline, which primarily uses extrinsic data but never-the-less incorporates Genscan (Burge and Karlin, 1997) predictions.

We downloaded and processed public RNAseq data to obtain an independent option for evaluating model performance. We selected three each of plant and animal genomes for detailed evaluation with RNAseq. These were selected to have relatively good (*Manihot esculenta* and *Papio anubis*), typical (*Medicago truncatula* and *Equus caballus*) and poor (*Theobroma cacao* and *Petromyzon marinus*) performance compared to AUGUSTUS (Supplementary Fig. S7 and S8) within our generalizable range (i.e. excluding the outgroups algae and invertebrates). Selections were further constrained by the availability of stranded RNAseq data.

For each of these six species the following search was performed on Sequence Read Archive (Leinonen *et al.*, 2010) ‘((‘<species name>’[Organism] OR <species name>[All Fields]) AND stranded[All Fields]) AND (‘biomol rna’[Properties] AND ‘library layout paired’[Properties])’. If more than 50 samples were identified, every Nth sample was selected so that in total under 50 samples were chosen for further processing (see Supplementary Table S15). Each sample was prepped, mapped and quality controlled in a pipeline using Trimmomatic (Bolger *et al.*, 2014), Hisat2 (Kim *et al.*, 2019), Samtools (Li *et al.*, 2009), PicardTools (http://broadinstitute.github.io/picard/), FastQC (www.bioinformatics.babraham.ac.uk/projects/fastqc/) and MultiQC (Ewels *et al.*, 2016; see Supplementary Table S16 for details). This pipeline was automated and the results visualized with the code available here (https://github.com/weberlab-hhu/RNAsleek). The samples were filtered to those with relatively high mapping rates, high mapping to exonic relative to non-exonic regions, low 3’ bias, normal FastQC and Trimmomatic stats, and a stranded mapping pattern (2nd read is sense strand). If more remained, seven of the high quality samples were selected randomly.

Finally, the selected and mapped RNAseq samples (Supplementary Table S15) were merged with Samtools and quantified to get the coverage (number of reads matching; i.e. cigar =, M or X), and spliced coverage (number of reads with gap or splice; i.e. cigar N or D) for every base pair in the genomes as implemented in Helixer’s ‘training_rnaseq.py’ script.

### 2.9 *In Silico* mutagenesis

To get a better sense of how our models are making their predicitions, two types of *In Silico* mutagenesis were performed. Base pairs of known motifs at targetted splice sites, start and stop codons were replaced with ’N’ characters (encoded as [0.25, 0.25, 0.25, 0.25]) to see how the network responded to an absence of the motif. We also manipulated coding potential (Brocchieri *et al.*, 2005) by scambling the chosen region in 3 bp steps to remove any codon-positional biases without changing the overall base composition. Perturbations were performed on an arbitrarily selected example gene in *M.esculenta* (Manes.01G003200.1.v6.1), and predictions for the modified sequence were created with the single best land plant bLSTM model (plants_a_e10.h5) with overlapping on.

## 3 Results

Figure 2a shows the side-by-side comparison of the distribution of performances on all non-training genomes in the animal and plant case by Subgenic F1 versus the reference. In the case of Helixer, these scores represent cross-species predictions. We also compare the median performances of different configurations of Helixer with AUGUSTUS and a dilated CNN architecture in Table 3. The results show a clear improvement over the AUGUSTUS both in higher median performance and reduced spread. We also outperform a dilated CNN architecture, and, for plants the hybrid DanQ architecture. Interestingly, during the review process, a single DanQ model topped the performance of the single best vertebrate bLSTM model, which will warrant further investigation.

**Table 3.** Summary of experimental results

	Animals	Plants
AUGUSTUS	0.632	0.757
Dilated CNN	0.666	0.802
DanQ hybrid	0.788	0.813
bLSTM model	0.770	0.833
bLSTM model + varied input length	0.834	–
bLSTM model (+varied input length) + overlapping	0.844	0.843
Ensemble of 8 (+varied input length) + overlapping	0.868	0.863

*Note:* Values are the median in Subgenic F1 versus the reference across all evaluation and test genomes of the respective group. Varied input length was only used in the animal case. The shown best bLSTM model (for vertebrates: animals_a_e07, for land plants: plants_a_e10) was chosen out of the eight models of the ensemble for having the best performance on the validation set of the training genomes.

Our models, however, tend to perform less well for the very smallest and largest genomes or species that are phylogenetically the furthest away from our training genomes. This is the case for both animals and plants and is visualized in Supplementary Figures S1 and S3. We do not, for example, consistently predict very well on the algae nor on the non-avian reptiles. While one of the algae was included in our training genomes it accounted for a tiny proportion of the total training data and the non-avian reptiles were not included at all.

AUGUSTUS outperforms us on some of the smallest genomes, but falls off much more drastically as the genomes get larger. The difference in prediction quality is especially strong for mammals, which tend to have big genomes with very long genes as well as the largest plants. Supplementary Figures S2 and S4 display the comparison to AUGUSTUS by phylogenetic position.

Two techniques were used during inference to improve performance and limit model bias. The usage of longer input lengths helped especially for genomes that tend to have longer genes and was enabled by our model architecture being a relatively simple BLSTM stack without any fully connected layers on top. We also constructed the final predictions out of overlapping ones, which greatly helped to reduce prediction bias in most genomes. To our knowledge, neither technique has been used before in a model developed for gene annotation.

**Fig. 3.**
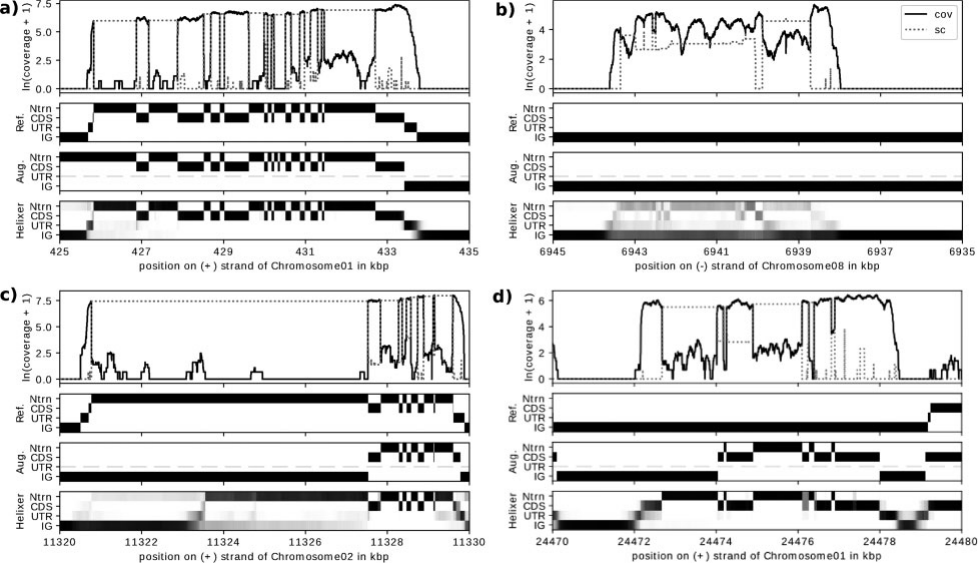
Four example helixer predictions in the context of RNAseq data, the reference and AUGUSTUS’ prediction for *M.esculenta*. The examples were chosen so that (**a**) Helixer had high accuracy against the reference and the reference was supported by the RNAseq data, (**b**) Helixer had high accuracy but the reference was not supported by the RNAseq data, (**c**) Helixer had low accuracy and the reference was supported by the RNAseq data and (**d**) Helixer had low accuracy but the reference was not supported by RNAseq data. Feasibility for visualization was a major secondary consideration. Each subplot shows from top to bottom (i) the natural log of the coverage (‘cov’, solid) and spliced coverage (‘sc’, dotted) + 1, (ii) the reference annotation in matrix form, (iii) AUGUSTUS’ predictions in matrix form (the dashed line is a reminder that no UTR predictions are expected) and (iv) Helixer’s predictions. The reference and AUGUSTUS have either 0 (white) or 1 (black) for each base pair and category, while Helixer emits a probability from 0-1 represented via gray-scale. ‘Ntrn’ stand for intron, and ‘IG’ stands for intergenic

To make an independent qualitative and quantitative evaluation of performance we compared Helixer predictions, AUGUSTUS predictions and the reference to RNAseq coverage data. Evaluation with RNAseq data was performed for three each of animal and plant species. RNAseq coverage provides support for an exonic annotation (UTR or CDS), spliced coverage provides support for an annotation of intron, and neither coverage nor spliced coverage is expected for intergenic annotations. Looking at selected subsequences (Fig. 3) we identified cases where RNAseq supported (i) both the annotations of the reference and Helixer, (ii) neither, (iii) the reference, but not Helixer and (iv) Helixer, but not the reference.

Helixer models do not yet have post-processing to make finalized single predictions, but instead output base-wise probabilities. We see that the model exhibits higher uncertainty around transitions between annotation classes, for instance between UTR and CDS, or more dramatically between UTR and intergenic even where Helixer predictions closely match the reference and RNAseq data (a). Helixer’s uncertainty around transitions from UTR to intergenic regions may relate to a fundamentally harder problem (there is no conserved motif at the site as is observed for splice sites and start/stop codons), lack of a precise one base pair biological site (Carninci *et al.*, 2006; Hon *et al.*, 2013) or noise in the reference, which we observed relative to the RNAseq data (Supplementary Figs S13 and S14).

Helixer models also sometimes showed uncertainty for larger regions. In some cases where Helixer did not receive RNAseq support for its highest probability annotation, it assigned a low but non-trivial probability to the RNAseq-supported exon/intron pattern (Fig. 3b and c, Supplementary Fig. S11c). However, in the extreme, there are cases where the Helixer model exhibits substantial indecision or confusion and shifts gradually between classes with no single class receiving a high probability for extended stretches (Supplementary Fig. S11c and d). Notably, in one of the examples (Supplementary Fig. S11d) the RNAseq shows evidence of alternative splicing; and in another (Supplementary Fig. S11c) Helixer’s prediction falls between that of the reference and of AUGUSTUS.

**Fig. 4.**
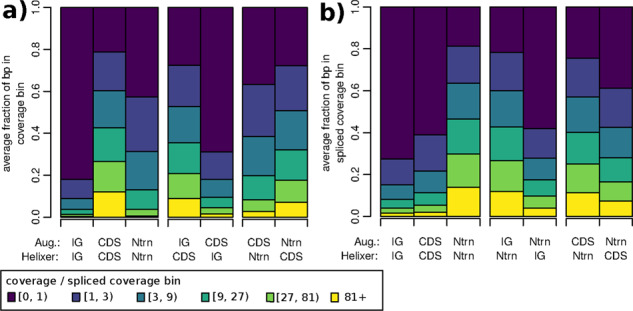
Fraction of bp with color-indicated (**a**) coverage and (**b**) spliced coverage of genomic positions broken down by the confusion matrix of AUGUSTUS’ and Helixer’s predictions. Categories are only displayed if they can be meaningfully compared by examining (a) coverage or (b) spliced coverage. The displayed fractions are the averages of the individual fractions for the six RNAseq-evaluation species. The left-most bars show cases where the two tools agree, while the remaining bars show paired conflicts. ‘Ntrn’ stand for intron, and ‘IG’ stands for intergenic

Coverage and Spliced coverage were broken down by the confusion matrix of both Helixer versus AUGUSTUS as well as Helixer versus the reference for all genomes (Fig. 4 and Supplementary Fig. S15). Where both tools agree on far left in the figure, coverage and spliced coverage closely matched expectations. Specifically for CDS: CDS and UTR: UTR most base pairs had some, and many had moderate or high coverage. The same pattern was seen for intron: intron and spliced coverage. Finally, intergenic: intergenic showed only a small fraction of base pairs with any of either coverage or spliced coverage. In all conflicts the amount of RNAseq support fell between the cases where tools agreed, indicating that all options were at least capable of finding weak-spots in the other annotations.

In cases of conflict, Helixer’s predictions were substantially more consistent with the RNAseq data than those of AUGUSTUS. Specifically, for base pairs where one tool predicted CDS and the other intergenic or intron, there was more coverage when Helixer predicted CDS (Fig. 4a). Similarly, for base pairs where one tool predicted intron and the other CDS or intergenic, there was more spliced coverage when Helixer predicted intron (Fig. 4b). These patterns were consistent in direction but varied in magnitude in the individual species, with the exception of *P.marinus* where Helixer and AUGUSTUS performed comparably at differentiating between CDS and intron regions (Supplementary Fig. S16).

RNAseq-based comparison of Helixer and the reference was less clear cut. Averaged across species, Helixer’s predictions received slightly more support than the reference when differentiating CDS, UTR and introns from intergenic; however Helixer’s predictions received slightly less support than the reference when differentiating CDS and UTR from introns (Supplementary Fig. S15). Performance varies between individual species, from *P.marinus*, where the reference receives more support in every conflict, to *T.cacao*, where Helixer models receive equivalent or more support in every conflict (Supplementary Fig. S17). Interestingly, these two species were selected as examples where Helixer had poor Subgenic F1 versus the reference; for the former the RNAseq confirms relatively weak performance for Helixer, while the for the latter RNAseq rather indicates a sub-par reference.

Finally, to take a first look at how Helixer models respond to known factors and motifs, we performed *in silico* mutagenesis on an example gene. The Helixer predictions were sensitive to perturbations of a donor and acceptor splice site, the stop codon and the coding potential, but indifferent to removal of the start codon (Supplementary Figs S18–S22). Most perturbations induced uncertainty in Helixer’s prediction, with the exception of removing the stop codon, where the network could simply use a second, proximal down-stream stop codon. This indicates the Helixer model often uses, but does not entirely rely on, known patterns.

## 4 Discussion

With Helixer, we introduce a novel, deep-learning based framework for the development of more effective tools for gene annotation prediction. Helixer outperforms AUGUSTUS on base pair wise metrics and on consistency with independent RNASeq data while also predicting cross-species for a wide range of genomes with one model.

We include trained models for land plants and vertebrates which achieve high prediction accuracy on gene annotation for broad phylogenetic groups (land plants and vertebrates, respectively). Within these groups, this eliminates the dependency on retraining and the expertise and data required therefore. Production of comparable, single-method annotations for broad groups has the potential to greatly facilitate downstream analyses; for instance it could avoid some of the inconsistency and errors that are otherwise seen in RNAseq analyses when different annotations are used (Torres-Oliva *et al.*, 2016; Zhao and Zhang, 2015).

We found our models to be highly sensitive to the training genomes we chose. A different set could lead to a significant shift in strengths and weaknesses of the model and a larger and more spread out set of high quality genomes could also result in a wider range of genomes with decent predictions. For this, more computational resources would be required. Simply training with a similar amount of data, but for a different group (e.g. invertebrates of fungi) could also be used to increase the functional predictive range.

An avenue for future research could be the addition of RNASeq data as additional input. This would bring Helixer on even footing with current tools and could lead to real world applicable performance improvements if the results of this work are any indication, as deep learning has been shown to excel in a multimodal settings (Ching *et al.*, 2018).

Finally, development of a post-processing method to go from base pair wise predictions to integrated predictions for whole transcripts at each loci could both further improve performance and would greatly increase real world applications. A post-processing method could for instance take the form of an HMM that worked with the current Helixer output instead of or in addition to raw sequence, or could even take the form of additional neural network layers that output precise locations of transitions (e.g. start & stop codons).

## Supplementary Material

btaa1044_Supplementary_Data
