# Supplementary material for: Helixer: cross-species gene annotation of large eukaryotic genomes using deep learning
Source: Bioinformatics. 2020 Dec 16;36(22-23):5291–8. doi: 10.1093/bioinformatics/btaa1044 (PMC8016489; doi:10.1093/bioinformatics/btaa1044)
Supplement: btaa1044_Supplementary_Data [file btaa1044_supplementary_data.zip › supplements.pdf]

Supplemental Materials for Helixer: Cross-species Gene Annotation of Large Eukaryotic Genomes Using Deep Learning

Felix Stiehler[1], Marvin Steinborn[1], Stephan Scholz, Daniela Dey, Andreas P.M. Weber[1], and Alisandra K. Denton[1]

[1]Institue for Plant Biochemistry, Heinrich-Heine-University, Dusseldorf, D-40225, Germany

Contents

1 Data collection and preparation **2**

2 Model Evaluation by Species **7**
2.1 Animal Data, Vertebrate Model 7
2.2 Plant Data, Land Plant Model 16

3 Architecture Selection and Hyperparameter Optimization **19**
3.1 BLSTM Model Architecture 19
3.2 DCNN Model Architecture 23
3.3 DanQ Model Architecture 24
3.4 Data Hyperparameters 25

4 RNAseq Evaluation Setup **26**

5 Longer Sequence Input **30**

6 Effect of Overlapping by Species **31**
6.1 Animal Data, Vertebrate Model 31
6.2 Plant Data, Land Plant Model 45

7 Training Data statistics **49**

8 Detailed results **50**

9 Evaluation against RNAseq **50**

10 *In silico* mutagenesis **58**

1 Data collection and preparation

This section describes exactly which animal and plant genomes were downloaded from Ensemble (Table S1) and Phytozome13 (Table S2), respectively. Further we describe assesment of genome quality based on metadata.

For each acquired genome we assembled a set of metadata for assessing genome and annotation quality. This metadata includes basic length and composition statistics with Quast (Gurevich *et al.*, 2013); bp content, 2-mer content with Jellyfish (Marçais and Kingsford, 2011); conserved gene content of the genome, transcriptome, and proteome (Simão *et al.* (2015); the transcriptome and proteome used were generated from the genome and gff3 file using gffread from Trapnell *et al.* (2012)); and finally simple counts of genic features from the gff3 files.

While the metadata ultimately does not provide a way to quantify "genome quality", there were several features that were, all the same, particularly useful to consider when evaulating, e.g. whether a particular genome was of sufficient quality to be used for training. Often these features did not directly measure quality of the annotation, but still reflect the time, resources and skill invested in creating the genome and annotation.

- Assembly Quality
  - Assembly contiguity–as measured by N50 / total assembly size
- Annotation Quality
  - Gene detection consistency–we looked at the difference between complete BUSCOs that could be identified in the proteom and genome. An inability to identify BUSCOs in the proteome that could alredy be identified in the genome without employing an extensive annotation pipeline is a sign of poor annotation quality.
  - Alternative splicing–we looked at two measures of alternative splicing annotation: the ratio mRNA to gene gff3 features, and the ratio of 'single' to 'duplicated' BUSCOs identified in the transcriptome. For species where extensive alternative splicing was expected, its absence indicates an absence of effort or extrinsic information inclusion in gene annotation.
  - UTRs–The average of 3' and 5' UTR features per mRNA feature. A handful of genomes included no, or very few, UTR annotations, and were thus innapropriate for training a model to predict UTRs.

All of the raw metadata and the four derived features mentioned above can be found in dataset S1.

Ensembl98 name	Version info	Test
acanthochromis_polyacanthus	ASM210954v1	
ailuropoda_melanoleuca	ailMel1	
amphilophus_citrinellus	Midas_v5	
amphiprion_ocellaris	AmpOce10	
amphiprion_percula	Nemo_v1	
anabas_testudineus	fAnaTes11	
anas_platyrhynchos_platyrhynchos	CAU_duck10	
anolis_carolinensis	AnoCar20	
anser_brachyrhynchus	ASM259213v1	
aotus_nancymaae	Anan_20	
apteryx_haastii	aptHaa1	
apteryx_owenii	aptOwe1	
astatotilapia_calliptera	fAstCal12	
astyanax_mexicanus	Astyanax_mexicanus-20	
betta_splendens	fBetSpl52	
bison_bison_bison	Bison_UMD10	
bos_indicus_hybrid	UOA_Brahman_1	
bos_mutus	BosGru_v20	
bos_taurus	ARS-UCD12	
caenorhabditis_elegans	WBcel235	
calidris_pygmaea	ASM369795v1	
callithrix_jacchus	ASM275486v1	
callorhinchus_milii	Callorhinchus_milii-613	
canis_familiaris	CanFam31	
canis_lupus_dingo	ASM325472v1	
capra_hircus	ARS1	
carlito_syrichta	Tarsius_syrichta-201	
castor_canadensis	can_genome_v10	
cavia_aperea	CavAp10	
cavia_porcellus	Cavpor30	
cebus_capucinus	Cebus_imitator-10	
cercocebus_atys	Caty_10	
chelonoidis_abingdonii	ASM359739v1	
chinchilla_lanigera	ChiLan10	
chlorocebus_sabaeus	ChlSab11	
chrysemys_picta_bellii	Chrysemys_picta_bellii-303	
ciona_intestinalis	KH	
ciona_savignyi	CSAV20	
clupea_harengus	Ch_v202	
colobus_angolensis_palliatus	pa_10	
cottoperca_gobio	fCotGob31	
coturnix_japonica	Coturnix_japonica_20	
cricetulus_griseus_picr	CriGri-PICR	
crocodylus_porosus	CroPor_comp1	
cynoglossus_semilaevis	Cse_v10	
cyprinodon_variegatus	C_variegatus-10	
dasypus_novemcinctus	Dasnov30	
denticeps_clupeoides	fDenClu11	
dipodomys_ordii	Dord_20	
dromaius_novaehollandiae	droNov1	
drosophila_melanogaster	BDGP622	
echinops_telfairi	TENREC	
electrophorus_electricus	Ee_SOAP_WITH_SSPACE	
eptatretus_burgeri	Eburgeri_32	
equus_asinus_asinus	ASM303372v1	
equus_caballus	EquCab30	
erinaceus_europaeus	HEDGEHOG	
erpetoichthys_calabaricus	fErpCal11	
esox_lucius	Eluc_V3	
felis_catus	Felis_catus_90	
ficedula_albicollis	FicAlb_14	
fukomys_damarensis	DMR_v10	
fundulus_heteroclitus	Fundulus_heteroclitus-302	
gallus_gallus	GRCg6a	

Ensembl98 name	Version info	Test
gambusia_affinis	ASM309773v1	
gasterosteus_aculeatus	BROADS1	
gopherus_agassizii	ASM289641v1	
gorilla_gorilla	gorGor4	
gouania_willdenowi	fGouWil21	
haplochromis_burtoni	AstBur10	
heterocephalus_glaber_female	HetGla_female_10	
hippocampus_comes	H_comes_QL1_v1	
homo_sapiens	GRCh38	
hucho_hucho	ASM331708v1	
ictalurus_punctatus	IpCoco_12	
ictidomys_tridecemlineatus	SpeTri20	
jaculus_jaculus	JacJac10	
kryptolebias_marmoratus	ASM164957v1	
labrus_bergylta	BallGen_V1	
larimichthys_crocea	L_crocea_20	
lates_calcarifer	ASB_HGAPassembly_v1	
latimeria_chalumnae	LatCha1	
lepidothrix_coronata	Lepidothrix_coronata-10	
lepisosteus_oculatus	LepOcu1	
lonchura_striata_domestica	LonStrDom1	
loxodonta_africana	loxAfr3	
macaca_fascicularis	Macaca_fascicularis_50	
macaca_nemestrina	Mnem_10	
manacus_vitellinus	ASM171598v2	
mandrillus_leucophaeus	le_10	
marmota_marmota_marmota	marMar21	
mastacembelus_armatus	fMasArm11	
meleagris_gallopavo	Turkey_201	
melopsittacus_undulatus	Melopsittacus_undulatus_63	
meriones_unguiculatus	MunDraft-v10	
mesocricetus_auratus	MesAur10	
microcebus_murinus	Mmur_30	
microtus_ochrogaster	MicOch10	
mola_mola	ASM169857v1	
monopterus_albus	M_albus_10	
mus_caroli	CAROLI_EIJ_v11	
mus_musculus	GRCm38	
mus_pahari	PAHARI_EIJ_v11	
mus_spicilegus	MUSP714	
mus_spretus	SPRET_EiJ_v1	
mustela_putorius_furo	MusPutFur10	
myotis_lucifugus	Myoluc20	
neolamprologus_brichardi	NeoBri10	
neovison_vison	v01	
nomascus_leucogenys	Nleu_30	
notamacropus_eugenii	Meug_10	
notechis_scutatus	TS10Xv2-PRI	
ochotona_princeps	OchPri20-Ens	
octodon_degus	OctDeg10	
oreochromis_niloticus	Orenil10	
ornithorhynchus_anatinus	OANA5	
oryctolagus_cuniculus	OryCun20	
oryzias_latipes	ASM223467v1	
otolemur_garnettii	OtoGar3	
ovis_aries	Oar_v31	
pan_paniscus	panpan11	
panthera_pardus	PanPar10	
panthera_tigris_altaica	PanTig10	
pan_troglodytes	Pan_tro_30	
papio_anubis	Panu_30	
parambassis_ranga	fParRan21	
paramormyrops_kingsleyae	PKINGS_01	
parus_major	Parus_major11	

Ensembl98 name	Version info	Test
pelodiscus_sinensis	PelSin_10	
periophthalmus_magnuspinnatus	fa	
peromyscus_maniculatus_bairdii	HU_Pman_21	
petromyzon_marinus	Pmarinus_70	
phascolarctos_cinereus	phaCin_unsw_v41	
piliocolobus_tephrosceles	ASM277652v2	
poecilia_formosa	PoeFor_512	
poecilia_latipinna	P_latipinna-10	
poecilia_mexicana	P_mexicana-10	
poecilia_reticulata	Guppy_female_10_MT	
pogona_vitticeps	pvi11	
pongo_abelii	PPYG2	
procavia_capensis	proCap1	
prolemur_simus	Prosim_10	
propithecus_coquereli	Pcoq_10	
pteropus_vampyrus	pteVam1	
pygocentrus_nattereri	Pygocentrus_nattereri-102	
rhinopithecus_bieti	ASM169854v1	
rhinopithecus_roxellana	Rrox_v1	
saimiri_boliviensis_boliviensis	SaiBol10	
salvator_merianae	HLtupMer3	
sarcophilus_harrisii	DEVIL70	
scleropages_formosus	ASM162426v1	
seriola_dumerili	Sdu_10	
seriola_lalandi_dorsalis	Sedor1	
sorex_araneus	COMMON_SHREW1	
spermophilus_dauricus	ASM240643v1	
sphenodon_punctatus	ASM311381v1	
stegastes_partitus	Stegastes_partitus-102	
sus_scrofa	Sscrofa111	
taeniopygia_guttata	taeGut324	
takifugu_rubripes	FUGU5	
tetraodon_nigroviridis	TETRAODON8	
theropithecus_gelada	Tgel_10	
tupaia_belangeri	TREESHREW	
tursiops_truncatus	turTru1	
urocitellus_parryii	ASM342692v1	
ursus_americanus	ASM334442v1	
ursus_maritimus	UrsMar_10	
vicugna_pacos	vicPac1	
vombatus_ursinus	bare-nosed_wombat_genome_assembly	
vulpes_vulpes	VulVul22	
xenopus_tropicalis	Xenopus_tropicalis_v91	
xiphophorus_couchianus	Xiphophorus_couchianus-401	
xiphophorus_maculatus	X_maculatus-50-male	
apteryx_rowi	aptRow1	✓
calidris_pugnax	ASM143184v1	✓
choloepus_hoffmanni	choHof1	✓
cyanistes_caeruleus	cyaCae2	✓
danio_rerio	GRCz11	✓
gadus_morhua	gadMor1	✓
junco_hyemalis	ASM382977v1	✓
macaca_mulatta	Mmul_10	✓
maylandia_zebra	M_zebra_UMD2a	✓
monodelphis_domestica	ASM229v1	✓
nannospalax_galili	galili_v10	✓
nothoprocta_perdicaria	notPer1	✓
numida_meleagris	NumMel10	✓
oryzias_melastigma	Om_v07RACA	✓
pundamilia_nyererei	PunNye10	✓
rattus_norvegicus	Rnor_60	✓
scophthalmus_maximus	ASM318616v1	✓
serinus_canaria	SCA1	✓
zonotrichia_albicollis	Zonotrichia_albicollis-101	✓

Table S1: Exact animal genome and annotation versions as download from Ensembl98 Genomes not included were redundant (same species, different accession, assembly or version) with one of the above genomes.

Species Name	Phytozome name	Phytozome ID	Version	Test
Ananas comosus	Acomosus	321	v3	
Amaranthus hypochondriacus	Ahypochondriacus	459	v2.1	
Asparagus officinalis	Aofficinalis	498	V1.1	
Arabidopsis thaliana	Athaliana	167	TAIR10	
Amborella trichopoda	Atrichopoda	291	v1.0	
Brachypodium distachyon	Bdistachyon	314	v3.1	
Brassica oleracea	Boleraceacapitata	446	v1.0	
Cicer arietinum	Carietinum	492	v1.0	
Citrus clementina	Cclementina	182	v1.0	
Capsella grandiflora	Cgrandiflora	266	v1.1	
Carica papaya	Cpapaya	113	ASGPBv0.4	
Chenopodium quinoa	Cquinoa	392	v1.0	
Chlamydomonas reinhardtii	Creinhardtii	281	v5.6	
Capsella rubella	Crubella	474	v1.1	
Citrus sinensis	Csinensis	154	v1.1	
Coccomyxa subellipsoidea C-169	CsubellipsoideaC169	227	v2.0	
Chromochloris zofingiensis	Czofingiensis	461	v5.2.3.2	
Daucus carota	Dcarota	388	v2.0	
Dunaliella salina	Dsalina	325	v1.0	
Eucalyptus grandis	Egrandis	297	v2.0	
Eutrema salsugineum	Esalsugineum	173	v1.0	
Fragaria vesca	Fvesca	501	v2.0.a2	
Glycine max	Gmax	508	Wm82.a4.v1	
Gossypium raimondii	Graimondii	221	v2.1	
Helianthus annuus	Hannuus	494	r1.2	
Hordeum vulgare	Hvulgare	462	r1	
Kalanchoe fedtschenkoi	Kfedtschenkoi	382	v1.1	
Lactuca sativa	Lsativa	467	v5	
Linum usitatissimum	Lusitatissimum	200	v1.0	
Malus domestica	Mdomestica	491	v1.1	
Manihot esculenta	Mesculenta	305	v6.1	
Mimulus guttatus	Mguttatus	256	v2.0	
Marchantia polymorpha	Mpolymorpha	320	v3.1	
Micromonas pusilla	MpusillaCCMP1545	228	v3.0	
Micromonas sp. RCC299	MspRCC299	229	v3.0	
Medicago truncatula	Mtruncatula	285	Mt4.0v1	
Olea europaea	Oeuropaea	451	v1.0	
Ostreococcus lucimarinus	Olucimarinus	231	v2.0	
Oryza sativa	Osativa	323	v7.0	
Oropetium thomaeum	Othomaeum	386	v1.0	
Prunus persica	Ppersica	298	v2.1	
Populus trichocarpa	Ptrichocarpa	444	v3.1	
Porphyra umbilicalis	Pumbilicalis	456	v1.5	
Ricinus communis	Rcommunis	119	v0.1	
Sorghum bicolor	Sbicolor	454	v3.1.1	
Setaria italica	Sitalica	312	v2.2	
Solanum lycopersicum	Slycopersicum	514	ITAG3.2	
Selaginella moellendorffii	Smoellendorffii	91	v1.0	
Spirodela polyrhiza	Spolyrhiza	290	v2	
Solanum tuberosum	Stuberosum	448	v4.03	
Theobroma cacao	Tcacao	233	v1.1	
Vitis vinifera	Vvinifera	457	v2.1	
Zostera marina	Zmarina	324	v2.2	
Zea mays	Zmays	493	RefGen_V4	
Volvox carteri	Vcarteri	317	v2.1	✓
Cucumis sativus	Csativus	122	v1.0	✓
Musa acuminata	Ppatens	318	v3.3	✓
Physcomitrella patens	Macuminata	304	v1	✓
Triticum aestivum	Taestivum	296	v2.2	✓
Arabidopsis lyrata	Alyrata	384	v2.1	✓

Table S2: Exact plant genome and annotation versions as download from Phytozome13. Genomes not included were either still under embargo or redundant (same species, different accession, assembly or version) with one of the above genomes.

2 Model Evaluation by Species

The following tables and plots show the prediction performance of Helixer for each species we worked with. The tables show a breakdown by basepair level accuracy, F1 score for each individual class and our two overall metrics, the Subgenic F1 and Genic F1. An F1 score of zero can indicate that the reference did not include that class.

The plots display the raw Subgenic F1 score as well as the difference to AUGUSTUS in Subgenic F1 with respect to the phylogenetic placement by the NCBI taxonomy database (Federhen, 2012).

2.1 Animal Data, Vertebrate Model

Genome	Acc Overall	IG F1	UTR F1	CDS F1	Intron F1	Subgenic F1	Genic F1
acanthochromis_polyacanthus	0.9036	0.9323	0.6	0.8663	0.8764	0.8753	0.8547
ailuropoda_melanoleuca	0.9343	0.9488	0	0.8757	0.9173	0.9153	0.913
amphilophus_citrinellus	0.8894	0.8501	0.0004	0.8609	0.9189	0.9123	0.9039
amphiprion_ocellaris	0.9001	0.9304	0.5473	0.8391	0.8763	0.8723	0.8472
amphiprion_percula	0.8942	0.923	0.5586	0.8375	0.8781	0.874	0.851
anabas_testudineus	0.9036	0.93	0.6076	0.8632	0.9037	0.8978	0.8709
anas_platyrhynchos_platyrhynchos	0.9218	0.9498	0.3189	0.8432	0.8579	0.857	0.8478
anolis_carolinensis	0.9051	0.942	0.4548	0.7631	0.788	0.7867	0.7776
anser_brachyrhynchus	0.9286	0.9533	0.3737	0.8571	0.8766	0.8754	0.8675
aotus_nancymaae	0.944	0.9629	0.3618	0.8557	0.9012	0.8996	0.895
apteryx_haastii	0.9025	0.9207	0.2082	0.8287	0.89	0.886	0.8793
apteryx_owenii	0.9072	0.935	0.3271	0.8354	0.8641	0.8623	0.8534
apteryx_rowi	0.9102	0.9366	0.3352	0.8395	0.8716	0.8696	0.8608
astatotilapia_calliptera	0.8663	0.9014	0.5666	0.7731	0.8438	0.8352	0.8169
astyanax_mexicanus	0.8849	0.9124	0.5784	0.8246	0.8632	0.8601	0.8454
betta_splendens	0.8423	0.8834	0.3626	0.8065	0.8227	0.8197	0.7842
bison_bison_bison	0.9021	0.9187	0	0.8575	0.8829	0.8819	0.8796
bos_indicus_hybrid	0.9304	0.9569	0.5481	0.8079	0.8505	0.8489	0.8414
bos_mutus	0.9234	0.9498	0.3885	0.843	0.8593	0.8586	0.8525
bos_taurus	0.9385	0.9622	0.4702	0.8379	0.8637	0.8627	0.8549
caenorhabditis_elegans	0.7423	0.8288	0.1477	0.7385	0.5006	0.5965	0.5709
calidris_pugnax	0.9166	0.9452	0.4135	0.8282	0.8568	0.8552	0.846
calidris_pygmaea	0.9195	0.9461	0.4404	0.8485	0.8682	0.867	0.8576
callithrix_jacchus	0.9352	0.9584	0.2818	0.833	0.8692	0.8679	0.8642
callorhinchus_milii	0.8735	0.9123	0.3684	0.8019	0.8083	0.8079	0.7951
canis_familiaris	0.9271	0.9542	0.2754	0.8239	0.8481	0.8472	0.8388
canis_lupus_dingo	0.937	0.9572	0.3463	0.8522	0.8976	0.8956	0.8906
capra_hircus	0.9395	0.962	0.4262	0.8521	0.8767	0.8757	0.8695
carlito_syrichta	0.9312	0.9554	0.4552	0.8477	0.8651	0.8644	0.8603
castor_canadensis	0.9177	0.9467	0.3725	0.8477	0.843	0.8433	0.8356
cavia_aperea	0.9405	0.9619	0	0.8462	0.8822	0.8805	0.8784
cavia_porcellus	0.9462	0.968	0.5285	0.8483	0.8652	0.8645	0.8563
cebus_capucinus	0.946	0.9659	0.5059	0.8455	0.8928	0.8911	0.885
cercocebus_atys	0.9457	0.9658	0.4896	0.8506	0.8917	0.8902	0.8834
chelonoidis_abingdonii	0.8808	0.9174	0.296	0.7582	0.8106	0.808	0.803
chinchilla_lanigera	0.9437	0.9664	0.507	0.8537	0.862	0.8617	0.8527
chlorocebus_sabaeus	0.9418	0.9645	0.4666	0.8569	0.8646	0.8643	0.8574
choloepus_hoffmanni	0.9036	0.9253	0	0.7481	0.8818	0.8744	0.8725
chrysemys_picta_bellii	0.8882	0.9294	0.3391	0.7153	0.7733	0.7706	0.7631
ciona_intestinalis	0.7474	0.8196	0.1528	0.6708	0.6638	0.6655	0.6383
ciona_savignyi	0.7662	0.826	0.071	0.6471	0.6982	0.6878	0.6764
clupea_harengus	0.863	0.9009	0.3972	0.7854	0.8341	0.8282	0.8018
colobus_angolensis_palliatus	0.9426	0.9631	0.5241	0.8557	0.8874	0.8863	0.8821
cottoperca_gobio	0.8889	0.9177	0.5953	0.8284	0.8756	0.8694	0.848
coturnix_japonica	0.9209	0.9461	0.3351	0.8604	0.8757	0.8747	0.8666
cricetulus_griseus_picr	0.9315	0.9546	0.3524	0.8647	0.8778	0.8772	0.8712
crocodylus_porosus	0.917	0.9472	0.3471	0.8005	0.8337	0.8323	0.8262
cyanistes_caeruleus	0.9228	0.9518	0.3437	0.8501	0.8445	0.8449	0.8362
cynoglossus_semilaevis	0.8751	0.9027	0.5883	0.8718	0.8702	0.8704	0.8456
cyprinodon_variegatus	0.8978	0.9298	0.5196	0.8531	0.8577	0.8573	0.835
danio_rerio	0.873	0.9151	0.3666	0.7688	0.7974	0.7954	0.7734
dasypus_novemcinctus	0.9371	0.9628	0.4583	0.8148	0.8328	0.832	0.8245
denticeps_clupeoides	0.8766	0.9144	0.3753	0.8112	0.8603	0.8528	0.814
dipodomys_ordii	0.9385	0.9599	0.4335	0.8549	0.8877	0.8863	0.8798
dromaius_novaehollandiae	0.9118	0.9414	0.41	0.8481	0.8486	0.8486	0.8395
drosophila_melanogaster	0.8409	0.8902	0.4738	0.9278	0.6762	0.7623	0.7467
echinops_telfairi	0.7972	0.8476	0	0.741	0.7035	0.7048	0.7033
electrophorus_electricus	0.8849	0.9176	0.3795	0.837	0.882	0.8749	0.84
eptatretus_burgeri	0.7511	0.8463	0.1436	0.5056	0.3671	0.371	0.3674
equus_asinus_asinus	0.9301	0.9476	0.2589	0.8651	0.9073	0.9055	0.9011

Genome	Acc Overall	IG F1	UTR F1	CDS F1	Intron F1	Subgenic F1	Genic F1
equus_caballus	0.9327	0.9571	0.2984	0.8417	0.8686	0.8675	0.86
erinaceus_europaeus	0.8202	0.863	0	0.7519	0.7471	0.7472	0.7454
erpetoichthys_calabaricus	0.8659	0.9173	0.3119	0.6275	0.6816	0.6794	0.6738
esox_lucius	0.8749	0.912	0.5692	0.8331	0.8293	0.8297	0.8123
felis_catus	0.9301	0.9557	0.3452	0.85	0.8561	0.8559	0.8494
ficedula_albicollis	0.9287	0.9559	0.5561	0.8493	0.8587	0.8581	0.8473
fukomys_damarensis	0.9434	0.9664	0.4492	0.8454	0.857	0.8565	0.8479
fundulus_heteroclitus	0.8951	0.927	0.543	0.8397	0.863	0.8609	0.84
gadus_morhua	0.886	0.8931	0	0.8422	0.8924	0.887	0.8806
gallus_gallus	0.9325	0.9575	0.4856	0.8165	0.8784	0.8743	0.8637
gambusia_affinis	0.8873	0.9182	0.5344	0.8719	0.875	0.8747	0.8437
gasterosteus_aculeatus	0.8774	0.8987	0.1991	0.8583	0.8969	0.8896	0.8568
gopherus_agassizii	0.8679	0.9133	0.3725	0.7513	0.7523	0.7522	0.7456
gorilla_gorilla	0.9422	0.9635	0.5272	0.8459	0.8808	0.8796	0.8748
gouania_willdenowi	0.8533	0.8976	0.2835	0.7337	0.805	0.7979	0.7674
haplochromis_burtoni	0.9008	0.9309	0.6155	0.8732	0.8684	0.8689	0.8492
heterocephalus_glaber_female	0.9416	0.9644	0.4958	0.828	0.8665	0.865	0.8565
hippocampus_comes	0.8695	0.9026	0.4316	0.8332	0.8622	0.8581	0.83
homo_sapiens	0.9492	0.9701	0.4823	0.8367	0.8648	0.8639	0.8553
hucho_hucho	0.874	0.9203	0.3735	0.7266	0.7813	0.7754	0.7537
ictalurus_punctatus	0.8791	0.9124	0.5555	0.8398	0.849	0.8481	0.8279
ictidomys_tridecemlineatus	0.944	0.9655	0.5465	0.8627	0.8771	0.8765	0.8686
jaculus_jaculus	0.9344	0.9578	0.2006	0.8484	0.8692	0.8685	0.8632
junco_hyemalis	0.9308	0.9538	0.4298	0.8626	0.893	0.8911	0.8797
kryptolebias_marmoratus	0.8961	0.9269	0.5683	0.8598	0.8748	0.873	0.8458
labrus_bergylta	0.894	0.9237	0.5812	0.8426	0.8788	0.8741	0.8513
larimichthys_crocea	0.8758	0.9079	0.3582	0.8572	0.8693	0.8677	0.8282
lates_calcarifer	0.8741	0.9062	0.3905	0.8387	0.867	0.8632	0.8276
latimeria_chalumnae	0.8817	0.9251	0.3917	0.7154	0.7628	0.7605	0.7529
lepidothrix_coronata	0.9261	0.9489	0.4337	0.8596	0.8919	0.8899	0.8805
lepisosteus_oculatus	0.9119	0.9401	0.5533	0.8676	0.8706	0.8703	0.8559
lonchura_striata_domestica	0.9261	0.9503	0.43	0.8522	0.8859	0.8837	0.8731
loxodonta_africana	0.9311	0.948	0	0.8752	0.9058	0.9044	0.9024
macaca_fascicularis	0.9452	0.9654	0.4878	0.8549	0.8897	0.8885	0.8821
macaca_mulatta	0.9416	0.9637	0.3885	0.8124	0.876	0.8738	0.8664
macaca_nemestrina	0.946	0.966	0.4801	0.8495	0.8916	0.8901	0.8833
manacus_vitellinus	0.9125	0.9426	0.3877	0.8354	0.8483	0.8475	0.8378
mandrillus_leucophaeus	0.9414	0.9623	0.5191	0.8525	0.8856	0.8844	0.8802
marmota_marmota_marmota	0.9238	0.9439	0.3195	0.8671	0.8922	0.8911	0.8869
mastacembelus_armatus	0.8885	0.9193	0.6051	0.8577	0.8714	0.8695	0.8464
maylandia_zebra	0.8784	0.9159	0.5813	0.762	0.8453	0.8352	0.8151
meleagris_gallopavo	0.912	0.9324	0.3154	0.8695	0.8911	0.8895	0.8826
melopsittacus_undulatus	0.9237	0.9493	0.4358	0.8516	0.8732	0.8719	0.8632
meriones_unguiculatus	0.9295	0.9535	0.4615	0.8735	0.8772	0.877	0.8682
mesocricetus_auratus	0.9448	0.9657	0.5247	0.8626	0.8837	0.8828	0.8759
microcebus_murinus	0.9429	0.9645	0.494	0.8372	0.8818	0.8801	0.8724
microtus_ochrogaster	0.9443	0.9646	0.5009	0.864	0.8923	0.8911	0.8831
mola_mola	0.8815	0.653	0	0.8648	0.9275	0.9197	0.9095
monodelphis_domestica	0.9257	0.9562	0.3206	0.7962	0.7846	0.785	0.7792
monopterus_albus	0.8819	0.9102	0.5546	0.8518	0.8671	0.8653	0.8461
mus_caroli	0.9372	0.9623	0.4256	0.8419	0.8348	0.8351	0.8305
mus_musculus	0.9542	0.9736	0.6151	0.8638	0.8659	0.8658	0.8569
mus_pahari	0.9352	0.9608	0.4229	0.8431	0.8344	0.8347	0.8301
mus_spicilegus	0.9339	0.9583	0.4436	0.8647	0.863	0.8631	0.8562
mus_spretus	0.9337	0.96	0.4093	0.8314	0.8267	0.8269	0.8224
mustela_putorius_furo	0.9317	0.9578	0.4319	0.857	0.8494	0.8497	0.8413
myotis_lucifugus	0.942	0.9622	0.0703	0.8534	0.9013	0.8983	0.8933
nannospalax_galili	0.9486	0.9685	0.4998	0.8469	0.8844	0.883	0.8767
neolamprologus_brichardi	0.8895	0.9094	0.4797	0.8523	0.8911	0.8868	0.8696
neovison_vison	0.9362	0.9565	0.4219	0.8752	0.895	0.8942	0.8894

Genome	Acc Overall	IG F1	UTR F1	CDS F1	Intron F1	Subgenic F1	Genic F1
nomascus_leucogenys	0.9433	0.9634	0.489	0.8497	0.8912	0.8897	0.8856
notamacropus_eugenii	0.8951	0.9203	0	0.7692	0.8619	0.8567	0.8552
notechis_scutatus	0.8491	0.89	0.2625	0.7682	0.7845	0.7834	0.776
nothoprocta_perdicaria	0.9062	0.8467	0	0.8533	0.9358	0.9294	0.9255
numida_meleagris	0.9276	0.952	0.3472	0.8565	0.8803	0.8788	0.871
ochotona_princeps	0.8374	0.8737	0	0.7899	0.7795	0.7799	0.7776
octodon_degus	0.9381	0.9602	0.198	0.8305	0.8797	0.8778	0.8721
oreochromis_niloticus	0.8967	0.9299	0.5269	0.8292	0.8495	0.8472	0.8284
ornithorhynchus_anatinus	0.9007	0.939	0.4056	0.7987	0.7723	0.7738	0.7665
oryctolagus_cuniculus	0.9025	0.9397	0.2709	0.7731	0.7786	0.7784	0.7715
oryzias_latipes	0.8864	0.9179	0.6008	0.8146	0.8671	0.8609	0.8401
oryzias_melastigma	0.8842	0.9189	0.5583	0.8437	0.8535	0.8524	0.8265
otolemur_garnettii	0.95	0.9681	0.1822	0.8767	0.9011	0.9001	0.8954
ovis_aries	0.9325	0.9569	0.2919	0.8441	0.8695	0.8684	0.8622
pan_paniscus	0.9532	0.972	0.5101	0.8553	0.8818	0.8809	0.8757
pan_troglodytes	0.9458	0.9665	0.499	0.8469	0.8822	0.881	0.8745
panthera_pardus	0.9366	0.9592	0.498	0.8428	0.8787	0.8774	0.8716
panthera_tigris_altaica	0.9289	0.954	0.4484	0.835	0.8641	0.863	0.8574
papio_anubis	0.943	0.9608	0.3066	0.8673	0.9071	0.9057	0.9019
parambassis_ranga	0.8571	0.8962	0.3735	0.8001	0.845	0.8375	0.7963
paramormyrops_kingsleyae	0.8926	0.925	0.591	0.8542	0.8639	0.8628	0.8418
parus_major	0.9307	0.955	0.3955	0.8683	0.8818	0.881	0.8703
pelodiscus_sinensis	0.8755	0.92	0.348	0.6937	0.7558	0.7529	0.744
periophthalmus_magnuspinnatus	0.9109	0.9341	0	0.8391	0.9087	0.8999	0.889
peromyscus_maniculatus_bairdii	0.9164	0.9274	0.002	0.8843	0.9059	0.905	0.903
petromyzon_marinus	0.9126	0.9496	0.094	0.714	0.8285	0.8155	0.8039
phascolarctos_cinereus	0.9254	0.953	0.3568	0.8302	0.8395	0.8392	0.8336
piliocolobus_tephrosceles	0.9374	0.9571	0.3872	0.8289	0.8967	0.8942	0.8908
poecilia_formosa	0.8969	0.9291	0.5553	0.8592	0.8762	0.8742	0.8445
poecilia_latipinna	0.8973	0.9281	0.4831	0.8562	0.879	0.8763	0.8486
poecilia_mexicana	0.8975	0.9298	0.5679	0.8584	0.8696	0.8683	0.8422
poecilia_reticulata	0.8654	0.8732	0.4516	0.8738	0.8772	0.8768	0.8591
pogona_vitticeps	0.8852	0.9268	0.2963	0.7753	0.7736	0.7737	0.7641
pongo_abelii	0.9393	0.9631	0.5004	0.8195	0.8536	0.8525	0.8472
procavia_capensis	0.8892	0.9093	0	0.7863	0.8673	0.8636	0.8619
prolemur_simus	0.9409	0.9534	0	0.885	0.9256	0.9239	0.9223
propithecus_coquereli	0.9408	0.9613	0.4612	0.854	0.8906	0.8892	0.8844
pteropus_vampyrus	0.8878	0.9056	0	0.8487	0.8679	0.8669	0.8647
pundamilia_nyererei	0.9026	0.9284	0.5454	0.8598	0.8918	0.8882	0.868
pygocentrus_nattereri	0.9061	0.9327	0.5784	0.8557	0.8799	0.878	0.8611
rattus_norvegicus	0.9436	0.9674	0.4773	0.8384	0.8336	0.8338	0.8247
rhinopithecus_bieti	0.9438	0.9645	0.4634	0.8464	0.8876	0.8861	0.8799
rhinopithecus_roxellana	0.9446	0.9647	0.5251	0.8489	0.8918	0.8901	0.8843
saimiri_boliviensis_boliviensis	0.9412	0.9625	0.5019	0.8488	0.884	0.8828	0.8771
salvator_merianae	0.8947	0.9334	0.3899	0.7896	0.782	0.7824	0.774
sarcophilus_harrisii	0.9245	0.9523	0.4197	0.8504	0.8387	0.8392	0.8336
scleropages_formosus	0.9055	0.9342	0.5883	0.8616	0.8885	0.8855	0.8633
scophthalmus_maximus	0.8944	0.9223	0.6249	0.8864	0.8822	0.8828	0.8575
serinus_canaria	0.9198	0.9463	0.4193	0.8563	0.8723	0.8713	0.8605
seriola_dumerili	0.9002	0.9263	0.5769	0.8745	0.8923	0.8902	0.8646
seriola_lalandi_dorsalis	0.903	0.9289	0.6162	0.8732	0.8925	0.89	0.8679
sorex_araneus	0.8512	0.8868	0	0.7659	0.7934	0.7923	0.7901
spermophilus_dauricus	0.9238	0.9418	0	0.8297	0.9005	0.8974	0.8951
sphenodon_punctatus	0.8151	0.884	0.2405	0.5038	0.5878	0.5838	0.58
stegastes_partitus	0.9075	0.9332	0.577	0.8697	0.8949	0.8922	0.87
sus_scrofa	0.9186	0.952	0.4099	0.731	0.8049	0.8018	0.7894
taeniopygia_guttata	0.8963	0.8963	0.2082	0.8642	0.9045	0.9017	0.8963
takifugu_rubripes	0.8759	0.9133	0.5619	0.8475	0.86	0.8575	0.8282
tetraodon_nigroviridis	0.8745	0.8944	0.1826	0.8695	0.8888	0.8838	0.8602
theropithecus_gelada	0.9554	0.9694	0.5335	0.8786	0.9283	0.9266	0.9233

Genome	Acc Overall	IG F1	UTR F1	CDS F1	Intron F1	Subgenic F1	Genic F1
tupaia_belangeri	0.8092	0.8584	0	0.732	0.7162	0.7167	0.7147
tursiops_truncatus	0.8792	0.8984	0	0.8473	0.8556	0.8552	0.8535
urocitellus_parryii	0.9391	0.9593	0.4454	0.8788	0.8948	0.8941	0.888
ursus_americanus	0.9168	0.9455	0.3849	0.8449	0.8512	0.8508	0.8433
ursus_maritimus	0.9257	0.9528	0.3279	0.8313	0.8528	0.852	0.8453
vicugna_pacos	0.8516	0.886	0	0.7738	0.7945	0.7939	0.7923
vombatus_ursinus	0.9315	0.9572	0.4186	0.8413	0.8494	0.8491	0.8425
vulpes_vulpes	0.9349	0.9595	0.3522	0.8444	0.8616	0.8609	0.8541
xenopus_tropicalis	0.8699	0.9175	0.317	0.7277	0.7438	0.7427	0.7289
xiphophorus_couchianus	0.8575	0.5599	0	0.8585	0.9119	0.9052	0.8932
xiphophorus_maculatus	0.8854	0.9156	0.5964	0.8368	0.871	0.8671	0.8439
zonotrichia_albicollis	0.9216	0.9482	0.382	0.8601	0.8667	0.8663	0.857

Table S3: Detailed results for all animal genomes.

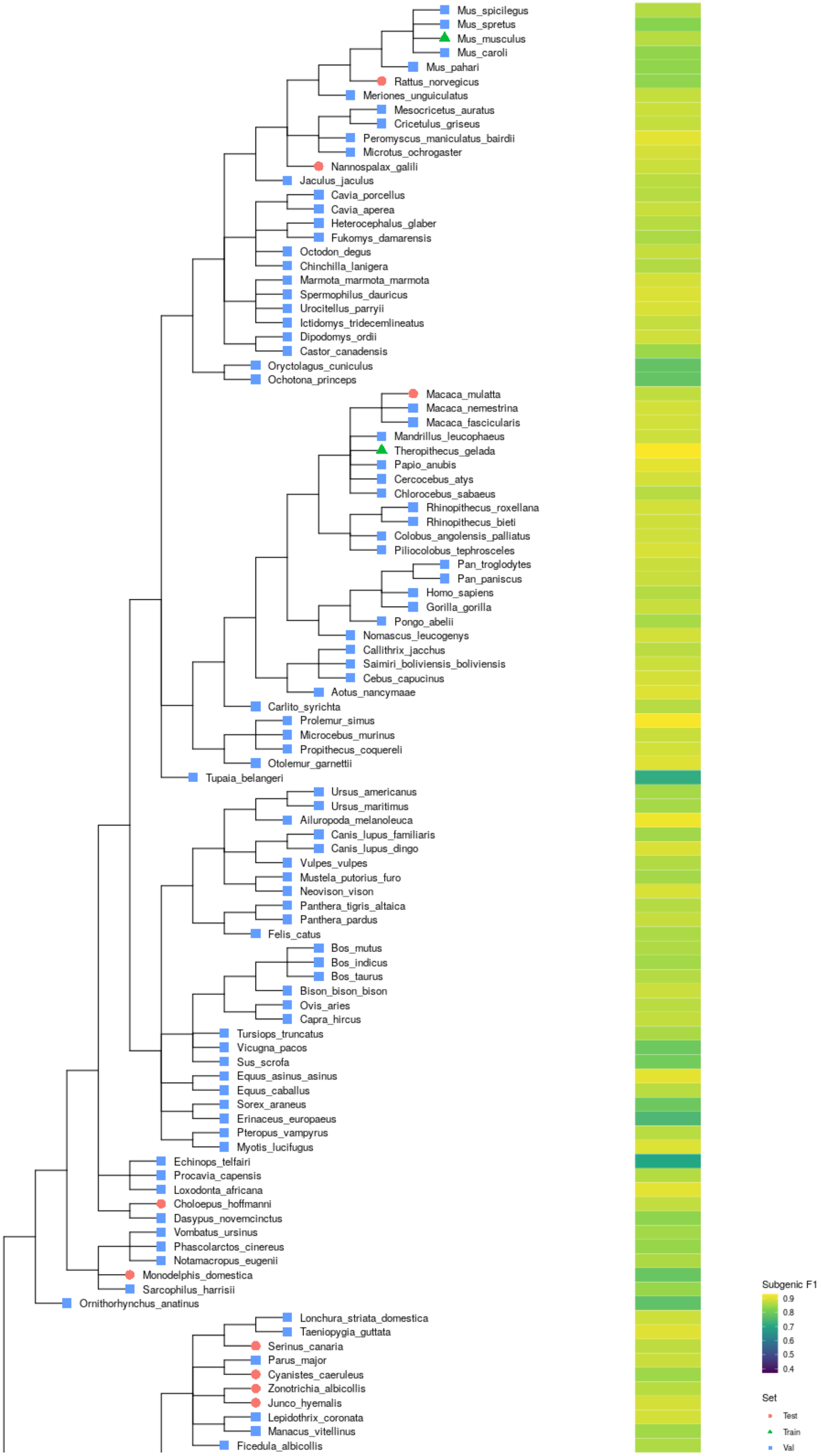
Mus_spicilegus
Mus_spretus
Mus_musculus
Mus_caroli
Mus_pahari
Rattus_norvegicus
Meriones_unguiculatus
Mesocricetus_auratus
Cricetulus_griseus
Peromyscus_maniculatus_bairdii
Microtus_ochrogaster
Nannospalax_galili
Jaculus_jaculus
Cavia_porcellus
Cavia_aperea
Heterocephalus_glaber
Fukomys_damarensis
Octodon_degus
Chinchilla_lanigera
Marmota_marmota_marmota
Spermophilus_dauricus
Urocitellus_parryii
Ictidomys_tridecemlineatus
Dipodomys_ordii
Castor_canadensis
Oryctolagus_cuniculus
Ochotona_princeps
Macaca_mulatta
Macaca_nemestrina
Macaca_fascicularis
Mandrillus_leucophaeus
Theropithecus_gelada
Papio_anubis
Cercocebus_atys
Chlorocebus_sabaeus
Rhinopithecus_roxellana
Rhinopithecus_bieti
Colobus_angolensis_palliatus
Piliocolobus_tephrosceles
Pan_troglodytes
Pan_paniscus
Homo_sapiens
Gorilla_gorilla
Pongo_abelii
Nomascus_leucogenys
Callithrix_jacchus
Saimiri_boliviensis_boliviensis
Cebus_capucinus
Aotus_nancymaae
Carlito_syrichta
Prolemur_simus
Microcebus_murinus
Propithecus_coquereli
Otolemur_garnettii
Tupaia_belangeri
Ursus_americanus
Ursus_maritimus
Ailuropoda_melanoleuca
Canis_lupus_familiaris
Canis_lupus_dingo
Vulpes_vulpes
Mustela_putorius_furo
Neovison_vison
Panthera_tigris_altaica
Panthera_pardus
Felis_catus
Bos_mutus
Bos_indicus
Bos_taurus
Bison_bison_bison
Ovis_aries
Capra_hircus
Tursiops_truncatus
Vicugna_pacos
Sus_scrofa
Equus_asinus_asinus
Equus_caballus
Sorex_araneus
Erinaceus_europaeus
Pteropus_vampyrus
Myotis_lucifugus
Echinops_telfairi
Procavia_capensis
Loxodonta_africana
Choloepus_hoffmanni
Dasypus_novemcinctus
Vombatus_ursinus
Phascolarctos_cinereus
Notamacropus_eugenii
Monodelphis_domestica
Sarcophilus_harrisii
Ornithorhynchus_anatinus
Lonchura_striata_domestica
Taeniopygia_guttata
Serinus_canaria
Parus_major
Cyanistes_caeruleus
Zonotrichia_albicollis
Junco_hyemalis
Lepidothrix_coronata
Manacus_vitellinus
Ficedula_albicollis
Subgenic F1
0.9
0.8
0.7
0.6
0.5
0.4
Set
Test
Train
Val

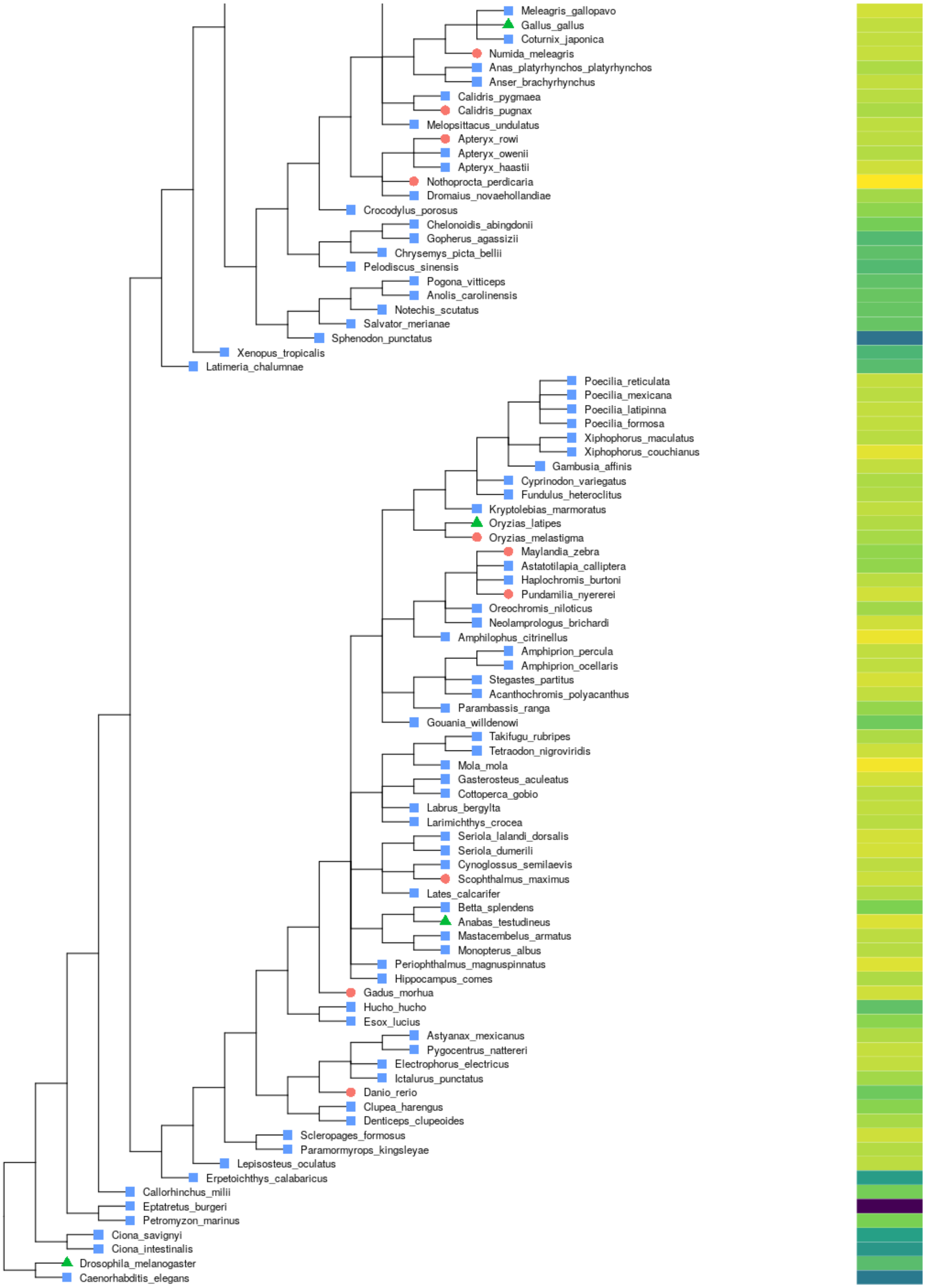

Figure S1: Subgenic F1 vs reference of Helixer's vertebrate model for all animal genomes shown by phylogenetic position.

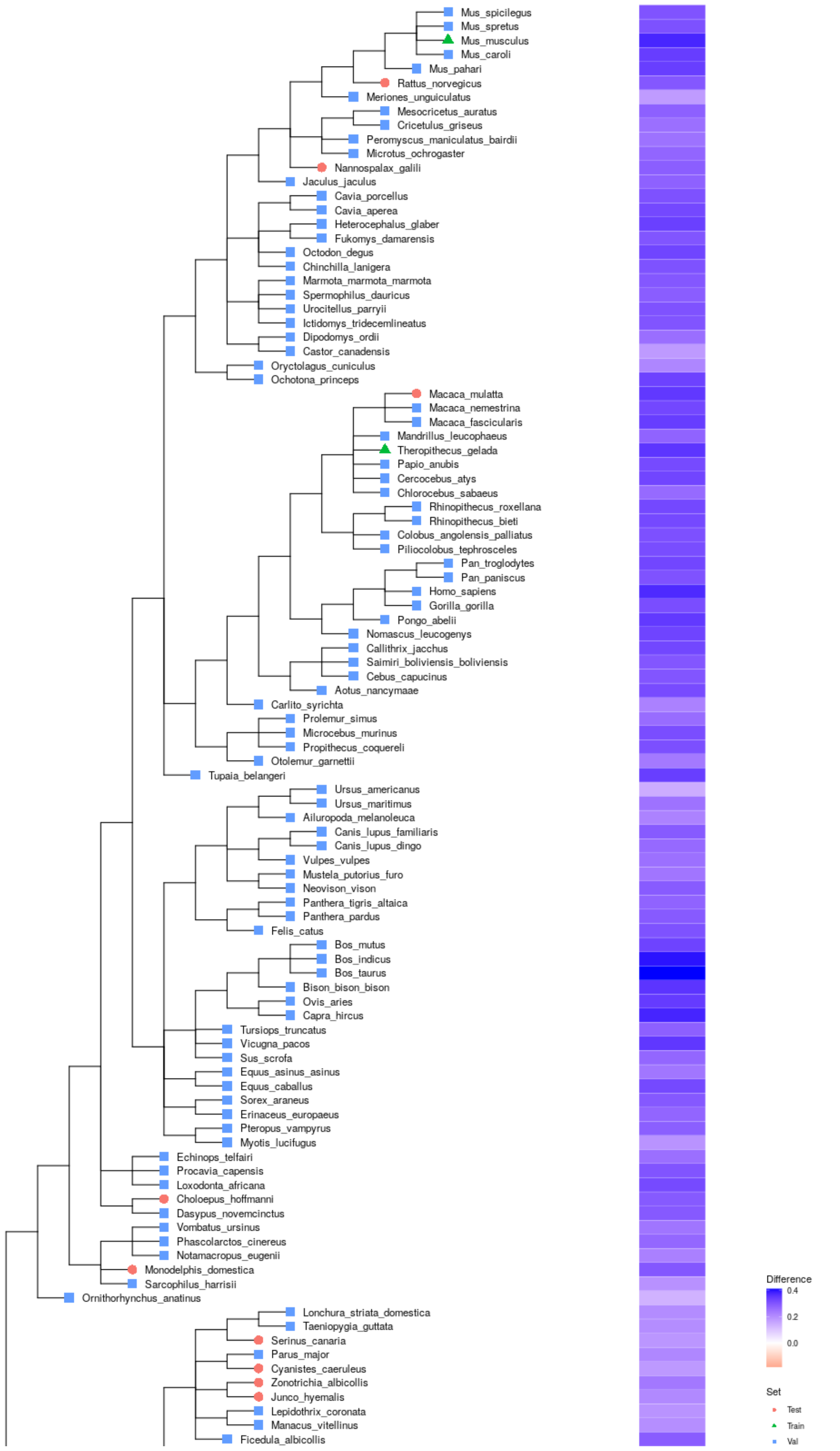
Mus_spicilegus
Mus_spretus
Mus_musculus
Mus_caroli
Mus_pahari
Rattus_norvegicus
Meriones_unguiculatus
Mesocricetus_auratus
Cricetulus_griseus
Peromyscus_maniculatus_bairdii
Microtus_ochrogaster
Nannospalax_galili
Jaculus_jaculus
Cavia_porcellus
Cavia_aperea
Heterocephalus_glaber
Fukomys_damarensis
Octodon_degus
Chinchilla_lanigera
Marmota_marmota_marmota
Spermophilus_dauricus
Urocitellus_parryii
Ictidomys_tridecemlineatus
Dipodomys_ordii
Castor_canadensis
Oryctolagus_cuniculus
Ochotona_princeps
Macaca_mulatta
Macaca_nemestrina
Macaca_fascicularis
Mandrillus_leucophaeus
Theropithecus_gelada
Papio_anubis
Cercocebus_atys
Chlorocebus_sabaeus
Rhinopithecus_roxellana
Rhinopithecus_bieti
Colobus_angolensis_palliatus
Piliocolobus_tephrosceles
Pan_troglodytes
Pan_paniscus
Homo_sapiens
Gorilla_gorilla
Pongo_abelii
Nomascus_leucogenys
Callithrix_jacchus
Saimiri_boliviensis_boliviensis
Cebus_capucinus
Aotus_nancymaae
Carlito_syrichta
Prolemur_simus
Microcebus_murinus
Propithecus_coquereli
Otolemur_garnettii
Tupaia_belangeri
Ursus_americanus
Ursus_maritimus
Ailuropoda_melanoleuca
Canis_lupus_familiaris
Canis_lupus_dingo
Vulpes_vulpes
Mustela_putorius_furo
Neovison_vison
Panthera_tigris_altaica
Panthera_pardus
Felis_catus
Bos_mutus
Bos_indicus
Bos_taurus
Bison_bison_bison
Ovis_aries
Capra_hircus
Tursiops_truncatus
Vicugna_pacos
Sus_scrofa
Equus_asinus_asinus
Equus_caballus
Sorex_araneus
Erinaceus_europaeus
Pteropus_vampyrus
Myotis_lucifugus
Echinops_telfairi
Procavia_capensis
Loxodonta_africana
Choloepus_hoffmanni
Dasypus_novemcinctus
Vombatus_ursinus
Phascolarctos_cinereus
Notamacropus_eugenii
Monodelphis_domestica
Sarcophilus_harrisii
Ornithorhynchus_anatinus
Lonchura_striata_domestica
Taeniopygia_guttata
Serinus_canaria
Parus_major
Cyanistes_caeruleus
Zonotrichia_albicollis
Junco_hyemalis
Lepidothrix_coronata
Manacus_vitellinus
Ficedula_albicollis
Difference
0.4
0.2
0.0
Set
Test
Train
Val

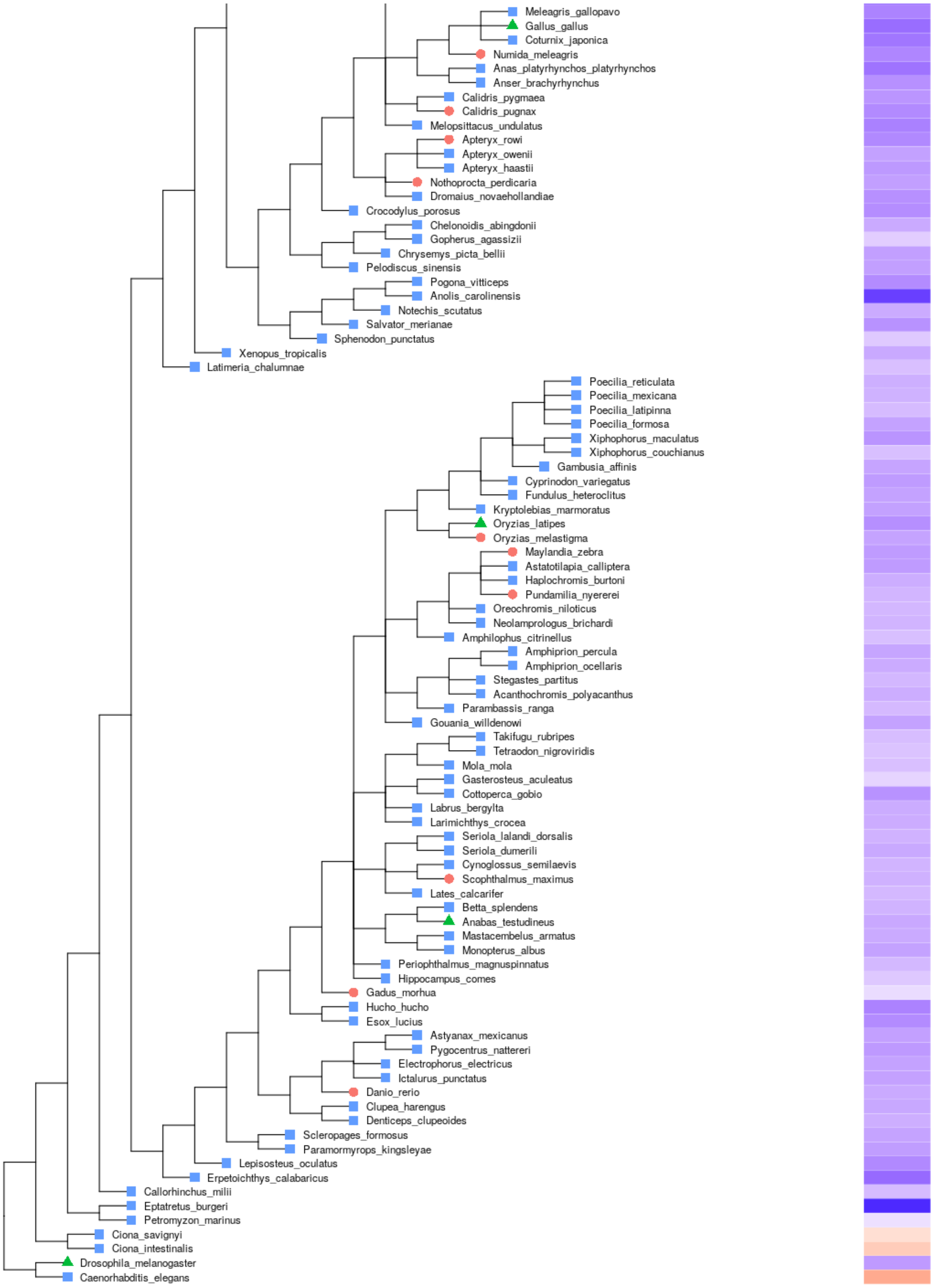

Figure S2: Difference in Subgenic F1 vs reference between the Helixer vertebrate model and AUGUSTUS for all animal genomes shown by phylogenetic position.

2.2 Plant Data, Land Plant Model

Genome	Acc Overall	IG F1	UTR F1	CDS F1	Intron F1	Subgenic F1	Genic F1
Ananas_comosus	0.8749	0.9224	0.5674	0.8591	0.7325	0.7691	0.753
Amaranthus_hypochondriacus	0.947	0.9728	0.5729	0.8535	0.8964	0.8856	0.864
Arabidopsis_lyrata	0.9438	0.9701	0.637	0.8957	0.8196	0.8649	0.8314
Asparagus_officinalis	0.6042	0.2635	0	0.8517	0.6979	0.7246	0.7206
Arabidopsis_thaliana	0.9542	0.9729	0.7294	0.953	0.9329	0.9456	0.9181
Amborella_trichopoda	0.6936	0.3451	0	0.8617	0.7816	0.7965	0.7933
Brachypodium_distachyon	0.9326	0.9625	0.6232	0.9027	0.8772	0.8879	0.8426
Brassica_oleracea	0.8648	0	0	0.9321	0.8785	0.911	0.9051
Cicer_arietinum	0.8583	0.7838	0	0.8872	0.8952	0.8921	0.8885
Citrus_clementina	0.9554	0.977	0.6277	0.9175	0.9027	0.9095	0.8771
Capsella_grandiflora	0.9465	0.9656	0.7117	0.9545	0.9139	0.9389	0.9145
Carica_papaya	0.9009	0.8926	0	0.9043	0.9156	0.9113	0.9057
Chenopodium_quinoa	0.944	0.9703	0.6057	0.855	0.8311	0.8382	0.8244
Chlamydomonas_reinhardtii	0.9484	0.9657	0.8039	0.9577	0.9546	0.9562	0.9254
Capsella_rubella	0.9455	0.9669	0.668	0.9485	0.9138	0.9352	0.9032
Cucumis_sativus	0.9515	0.9729	0.6174	0.9268	0.9237	0.9249	0.8962
Citrus_sinensis	0.9545	0.9766	0.5878	0.9147	0.9027	0.9082	0.8778
Coccomyxa_subellipsoidea_C-169	0.8467	0.8573	0.4164	0.8771	0.8896	0.8847	0.8409
Chromochloris_zofingiensis	0.8849	0.3095	0	0.9129	0.9299	0.9203	0.9027
Daucus_carota	0.8924	0.703	0	0.9284	0.9212	0.924	0.9175
Dunaliella_salina	0.7458	0.8473	0.1832	0.6152	0.4306	0.4585	0.4352
Eucalyptus_grandis	0.9447	0.9712	0.5327	0.8391	0.8245	0.8305	0.7956
Eutrema_salsugineum	0.9555	0.9755	0.6501	0.9301	0.8985	0.9179	0.8908
Fragaria_vesca	0.8776	0.9192	0.633	0.8666	0.7813	0.8218	0.7972
Glycine_max	0.9755	0.9886	0.6564	0.9265	0.9165	0.92	0.8825
Gossypium_raimondii	0.9784	0.9901	0.6836	0.9216	0.8835	0.9004	0.8701
Helianthus_annuus	0.9747	0.9875	0.4514	0.7758	0.6562	0.6975	0.6763
Hordeum_vulgare	0.9826	0.9917	0.4462	0.7774	0.624	0.6762	0.6503
Kalanchoe_fedtschenkoi	0.9415	0.9693	0.6067	0.9112	0.8569	0.8833	0.8372
Lactuca_sativa	0.98	0.9908	0.5771	0.867	0.7075	0.7848	0.7679
Linum_usitatissimum	0.8411	0.3605	0	0.9452	0.8435	0.8991	0.8912
Musa_acuminata	0.926	0.9598	0.4746	0.8856	0.8984	0.8946	0.8682
Malus_domestica	0.9629	0.9816	0.6214	0.9049	0.8661	0.8825	0.852
Manihot_esculenta	0.9748	0.9879	0.681	0.9365	0.9199	0.9261	0.8981
Mimulus_guttatus	0.9704	0.985	0.7101	0.9405	0.9234	0.9319	0.9062
Marchantia_polymorpha	0.9409	0.9708	0.6294	0.8786	0.8797	0.8792	0.8083
Micromonas_pusilla	0.6896	0.639	0.3364	0.7517	0.617	0.7358	0.7223
Micromonas_sp._RCC299	0.592	0.5639	0.2706	0.637	0.5212	0.6301	0.6152
Medicago_truncatula	0.9181	0.9474	0.6813	0.8625	0.8639	0.8633	0.8475
Olea_europaea	0.7935	0.7695	0	0.794	0.8262	0.8142	0.8096
Ostreococcus_sp._lucimarinus	0.2174	0.3241	0.0451	0.0746	0.0441	0.0734	0.0725
Oryza_sativa	0.9061	0.9439	0.6234	0.8367	0.827	0.8312	0.8044
Oropetium_thomaeum	0.8682	0.9194	0.5482	0.8605	0.8045	0.8275	0.8058
Physcomitrella_patens	0.9589	0.9816	0.637	0.8701	0.8554	0.8626	0.8064
Prunus_persica	0.9434	0.9688	0.6422	0.9089	0.8944	0.9012	0.8605
Populus_trichocarpa	0.9609	0.9806	0.6771	0.9244	0.916	0.9196	0.882
Porphyra_umbilicalis	0.7648	0.8529	0.2087	0.6096	0.2986	0.565	0.5139
Ricinus_communis	0.9256	0.9442	0.4673	0.8984	0.9364	0.9192	0.9055
Sorghum_bicolor	0.9654	0.983	0.5792	0.9033	0.8608	0.8775	0.8292
Setaria_italica	0.9561	0.9775	0.6527	0.9166	0.8933	0.9037	0.8646
Solanum_lycopersicum	0.9476	0.9725	0.4948	0.8826	0.8367	0.8509	0.8127
Selaginella_moellendorffii	0.7166	0.7977	0.1825	0.7436	0.435	0.662	0.6388
Spirodela_polyrhiza	0.7784	0	0	0.903	0.8414	0.8624	0.8529
Solanum_tuberosum	0.7272	0.0994	0	0.8551	0.8199	0.8335	0.8299
Triticum_aestivum	0.9238	0.9601	0.5238	0.8455	0.8356	0.8399	0.7961
Theobroma_cacao	0.8743	0.9295	0.5257	0.8677	0.545	0.6421	0.627
Volvox_carteri	0.8546	0.9045	0.5535	0.831	0.8241	0.8269	0.7706
Vitis_vinifera	0.7918	0.0076	0	0.9229	0.8613	0.8771	0.8724
Zostera_marina	0.9148	0.9463	0.5865	0.9052	0.8261	0.8571	0.8387
Zea_mays	0.9843	0.9931	0.6073	0.886	0.854	0.8643	0.8406

Table S4: Detailed results for all plant genomes.

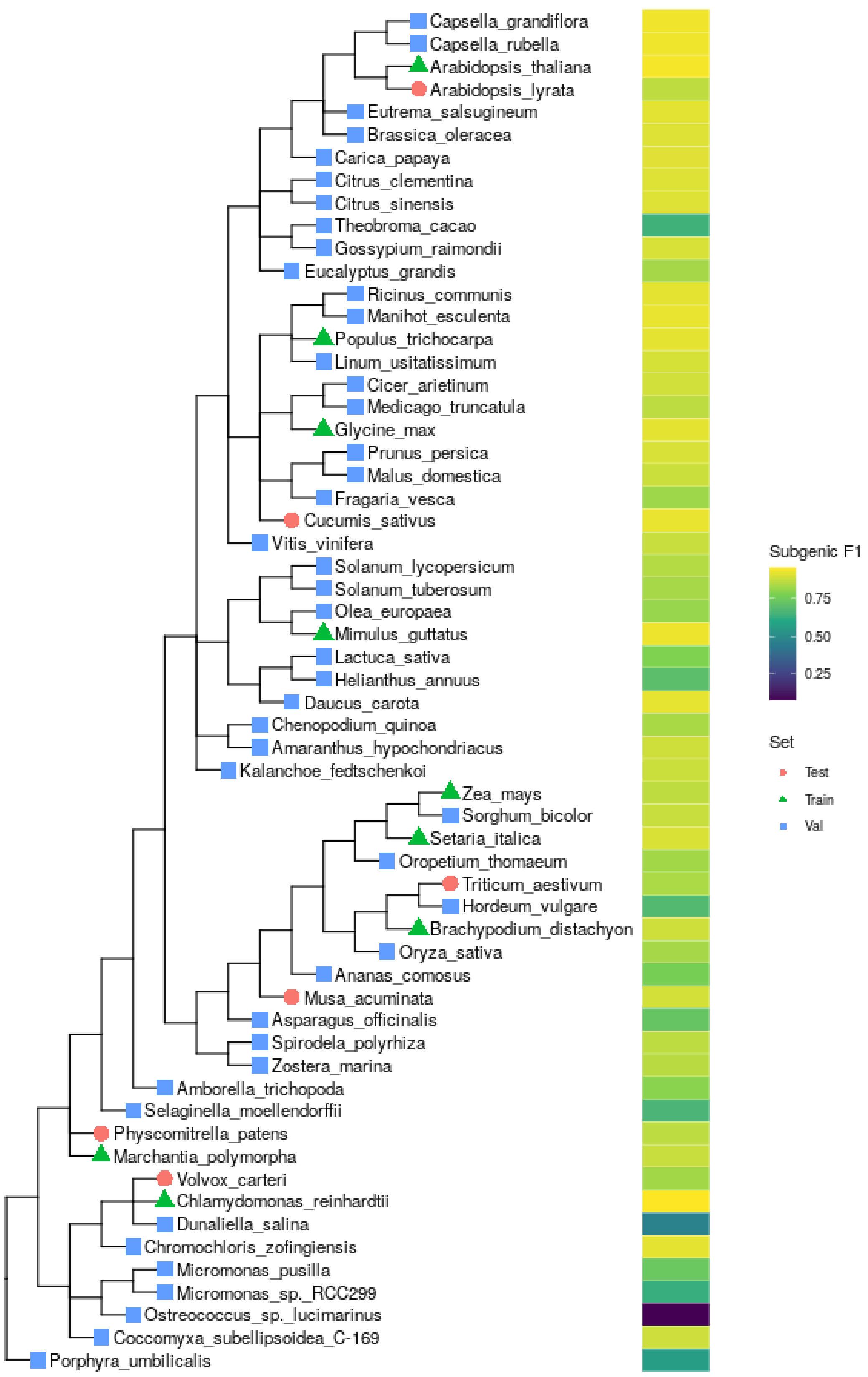

Figure S3: Subgenic F1 vs reference of the Helixer land plant model for all plant genomes shown by phylogenetic position.

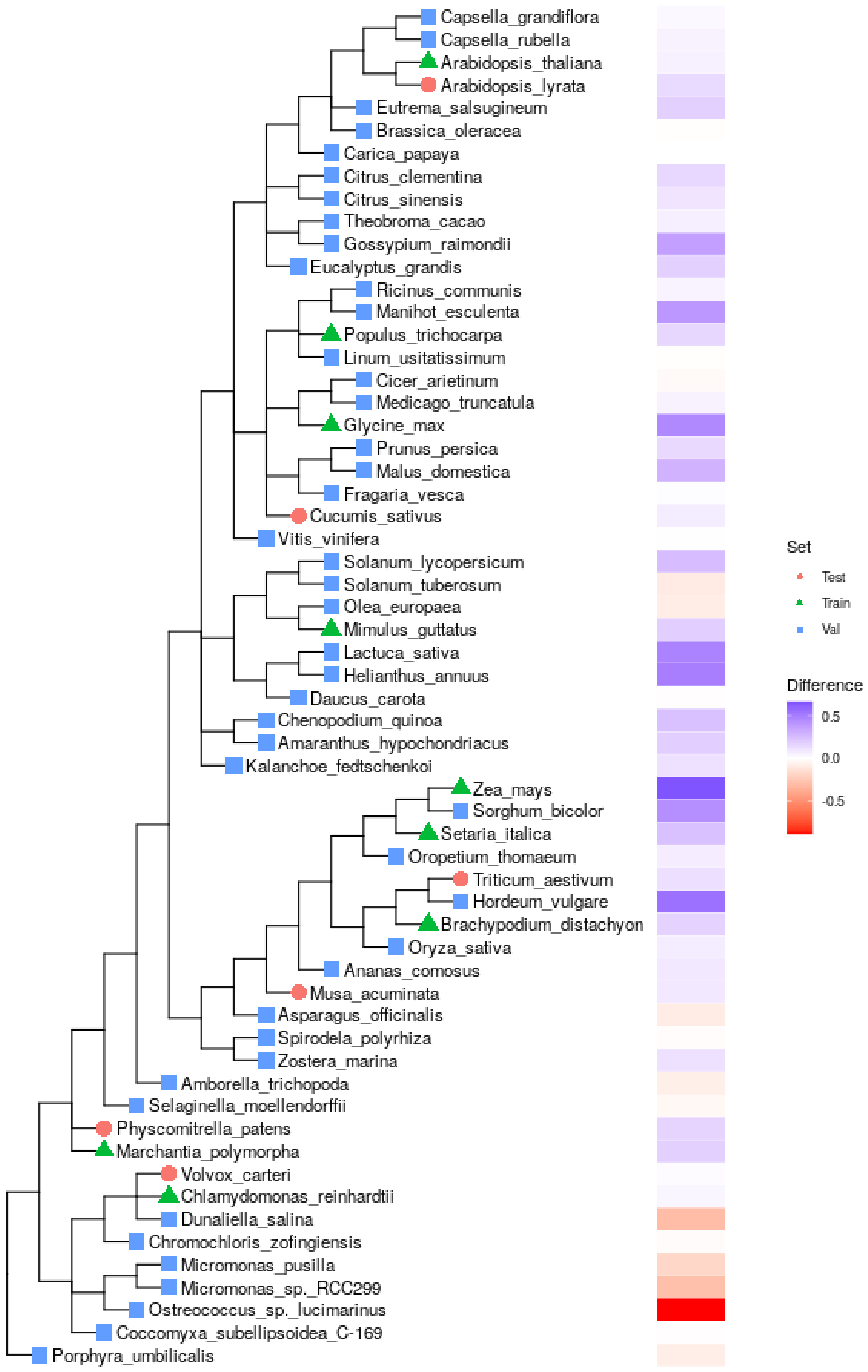

Figure S4: Difference in Subgenic F1 vs reference between the Helixer land plant model and AUGUSTUS for all plant genomes shown by phylogenetic position.

3 Architecture Selection and Hyperparameter Optimization

During development of Helixer three major classes of DNN architecture were trained, hyperoptimized, and evaluated in an iterative and intertwined fashion with modifications to the data and project as a whole.

Early efforts focussed on CNNs and then LSTMs, which consistently outperformed the CNNs. The early LSTM models were single layer and used no pooling. Intermediate efforts tried a hybrid architecture with an initial convolutional layer followed by an LSTM layer. This hybrid architecture was inspired by (Quang and Xie, 2016) and is hereafter called DanQ. DanQ models showed top performance and were focussed on until two major changes were made to the LSTMs. These changes were 1) using multi-layer LSTMs, and critically 2) using pooling to feed the LSTMs more than 1 bp per time step, which allowed the LSTMs to train more effectively on longer input data. With these changes, LSTMs surpassed the performance of DanQ, and were focussed on until near-publication.

For a final comparison, dilated CNN hyperparameters were re-optimized and a dilated CNN model was re-trained; while DanQ, which had received far more optimization during development, was simply retrained with previously selected hyperparameters on the final data (during the review process).

The data itself also changed during development, both in terms of which data was used and how it was presented to the network. Chronologically, we started with plant data, and expanded later to include animal data; experimenting with which exact training genomes worked best for each along the way. The gains of the larger chunk sizes (e.g. 20kbp) were only seen after pooling (for both DanQ and the LSTMs) was added.

While detailing the hundreds of training runs would add more confusion than clarity, we summarize below a list of most model and data hyperparameters that were attempted at some point during development, as well as the final selected parameters.

3.1 BLSTM Model Architecture

Parameter	Search Space
LSTM Layers	{1, 2, 3, 4, 5}
LSTM Units per layer	{2, 4, 8, 16, 32, 64, 128, 192, 256, 384, 512}
Learning rate	{1e-2, 1e-3, 1e-4, 1e-5}
Batch Size	52 {16, 32, 52, 64, 128}
Optimizer	{Adam, RMSprop, AdaGrad}
Clipnorm	{1.0, 2.5, 5.0, 10.0}
Loss	{Binary Crossentropy, Categorical Crossentropy}
Float Precision	{16 bit, 32 bit, 64 bit}
Pool Size	{1, 8, 10, 13, 15, 17, 20}
Dropout	{0.0, 0.05, 0.1, 0.15, 0.2}
Layer Normalization	{y, n}

Table S5: Summary of hyperparameters that were tried at some point during development for the bidirectional LSTM models. Parameters were not tried in a single optimization run, but rather some were tested and narrowed-down or decided-on early, some were repeatedly refined, and others were only implemented later.

Parameter	Value
LSTM Layers	4
LSTM Units per layer	256
Learning rate	1e-3
Batch Size	52
Optimizer	Adam
Clipnorm	1.0
Loss	Categorical Crossentropy
Float Precision	32 bit
Dropout	0.0
Layer Normalization	y

Table S6: The hyperparameters that were used for the training of both the vertebrate and land plant models. For more implementation details please consult the source code.

Class	Vertebrate Value	Land Plant Value
Intergenic	0.7	0.3
UTR	1.6	1.0
CDS	1.2	0.9
Intron	1.2	0.3

Table S7: The class weights used during training. We found this parameter to be highly impactful for establishing a more balanced performance across the different classes. These are the only hyperparameters that are different for vertebrate and land plant models. The exact weights were determined manually and partially reflect the difference in the class distributions of both datasets (see Table 2).

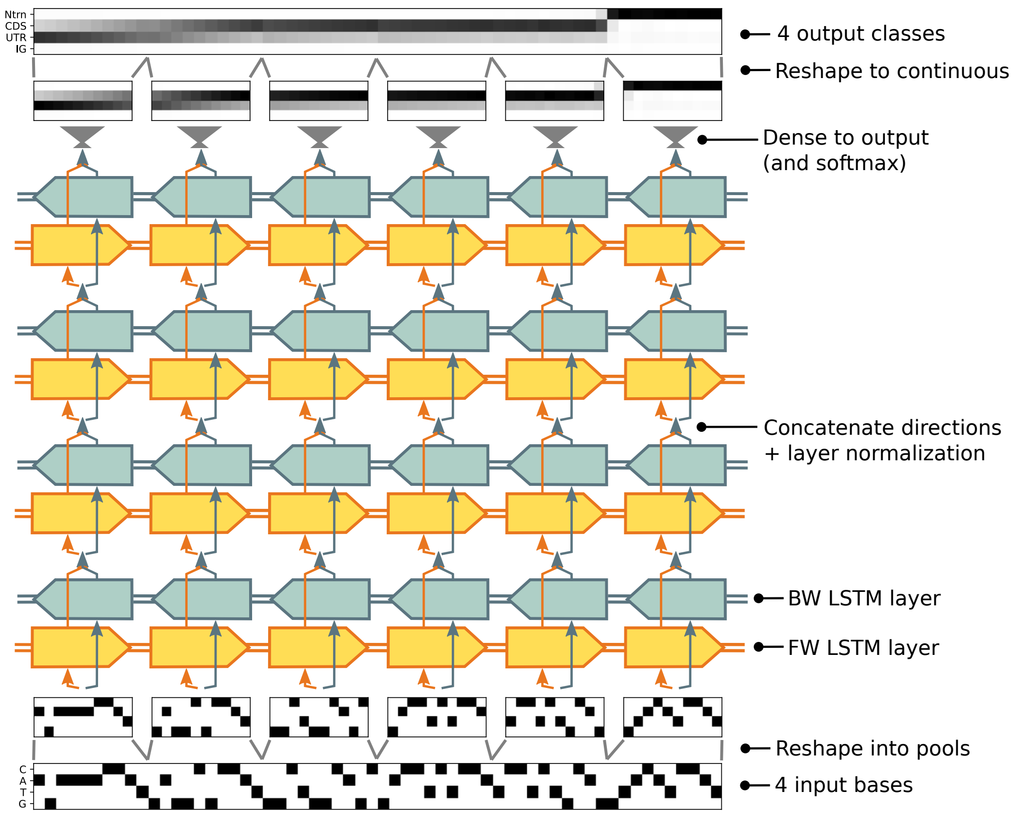

Figure S5: Depiction of final deep bLSTM model architechture. Sequence data is first reshaped so that 10bp of information are fed into each time step of the forward and backward LSTM. Each LSTM cell uses the standard Keras architecture (Fig S6).Once both directions are finished, outputs are concatenated and layer normalization is performed before the next bLSTM layer. The outputs of the last bLSTM are fed through a dense layer and softmax activation at each time step to produce 10bp worth of output. This pooled output is then reshaped to be continuous and match the original input. During training, 2,000 and not 6 time steps were used.

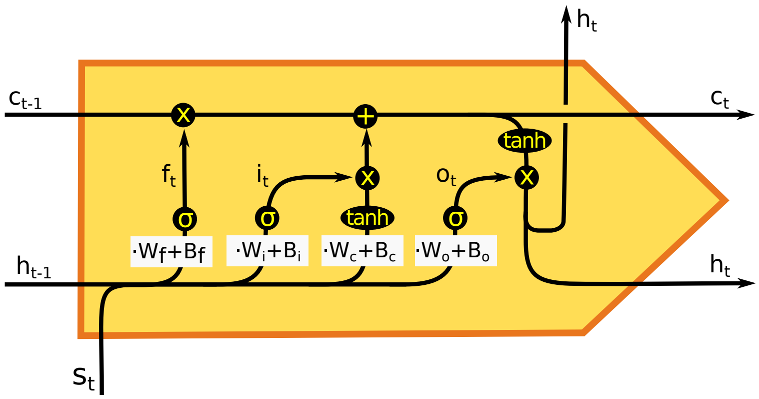

Figure S6: Depiction of single LSTM cell, using the default Keras implementation, with the parameter 'return_sequences' set to 'True' to return output for every time step ($_t$). Each timestep receives sequence (S) information (either DNA sequence or the sequence returned by the previous layer) and both outputs from the previous time step ($_{t-1}$)–hidden (h) and carry (c)–as input. The concatenation of S and h is copied into four pieces, each of which is multiplied by the corresponding weights and the bias is added. Three of the resulting matrices are sigmoid transformed to make a gate or mask, with values between 0 and 1. The resulting gates are the forget (f), input (i), and output (o), and are used to mask the old carry value, the updates to the carry value, and the output hidden values, respectively. The remaining matrix is tanh transformed to make candidate values between -1 and 1 that are multiplied by the input gate described above and then added to the carry values. Finally, the updated carry value is tanh transformed and multiplied by the output gate to make both the hidden values and the output sequence.

3.2 DCNN Model Architecture

Parameter	Search Space
Kernel Size	{4, 8, 12, 16}
Initial Filter Depth	{32, 64, 96, 128}
Number of Layers Before Doubling Filter Count	{1, 2}
Dilation Multiplier	{2, 3}
Number of Convolutional Layers	{2, 3, 4, 5, 6, 7, 8}
Number of Hidden Fully Connected (FC) Layers	{0, 1, 2}
Dropout Used on FC and Final Conv Layer Output	{0.0, 0.01, 0.1, 0.2, 0.3}
Learning Rate	{1e-3, 1e-4}

Table S8: Above are all parameters used during neural architecture search for the dilated CNN baseline. The same overall space was used for plant and animal data. We did, however, run multiple distinct searches that sometimes only operated over a subset of the given parameters. This was done as those seemed to be the most promising. We for example restricted the search space of the number of convolutional layers to the highest 3 values in later runs. Decisions were guided by the Genic F1 on the validation set of exactly the same data we trained our final LSTM models with. Runs with a Genic F1 below 0.5 after 10 epochs were stopped and the overall maximum epoch was 15. The performances seemed to be leveling off well before that. The batch size used for almost all runs was 32, the dilation was capped to 81 and the size of each fully connected layer was fixed at 128. New parameters where chosen at random. The implementation and parameter space is very roughly based on (Gupta and Rush, 2017) and can be found in the Helixer source code repository. In total, we trained 30 models with the animal data and 48 with the plant data.

Parameter	Vertebrate	Land Plants
Kernel Size	16	16
Initial Filter Depth	96	32
Number of Layers Before Doubling Filter Count	2	2
Dilation Multiplier	3	3
Number of Convolutional Layers	6	8
Number of Hidden Fully Connected (FC) Layers	2	0
Dropout Used on FC Output	0.0	0.0
Learning Rate	1e-4	1e-3

Table S9: The parameters of the dilated CNN model that performed the best according to the Genic F1 of the validation data of the respective training genomes. The best vertebrate model has circa 4.6 million parameters; the best land plant model around 2.1 million. Any unspecified parameters matched those of the bLSTM.

3.3 DanQ Model Architecture

Parameter	Search Space
Conv Kernal Size	{10, 18, 26, 34, 42}
Conv Filter Depth	{32, 64, 128, 192, 256, 320, 384, 512}
Post Conv layer Dropout	{0.0, 0.1, 0.2, 0.3, 0.4}
LSTM Units per layer	{32, 64, 128, 192, 256, 320, 384, 512}
Post LSTM layer Dropout	{0.0, 0.1, 0.2, 0.3, 0.4, 0.5, 0.6}
Learning rate	{1e-3, 1e-4}
Optimizer	{Adam, RMSprop}
Max Pooling Size	{1, 4, 5, 8, 10, 12, 15, 16, 20, 25}
Layer Normalization	{y, n}

Table S10: Summary of hyperparameters that were tried at some point during development for the DanQ models. Parameters were not tried in a single optimization run, but rather some were tested and narrowed-down or decided-on early, some were repeatedly refined, and others were only implemented later.

Parameter	Value
Conv Kernal Size	34
Conv Filter Depth	384
Post Conv layer Dropout	0.3
LSTM Units per layer	512
Post LSTM layer Dropout	0.5
Learning rate	1e-3
Optimizer	Adam
Max Pooling Size	10
Layer Normalization	y

Table S11: The hyperparameters that were used for the training of both the vertebrate and land plant models DanQ. For more implementation details please consult the source code. All unspecified parameters matched those of the bLSTMs.

3.4 Data Hyperparameters

Parameter	Search Space
Chunk Size in kbp	{1, 5, 10, 20, 30, 50, 100}

Table S12: The major numeric data-specific hyperparameter that was varied on multiple occasions before selecting the final value of **20kbp per chunk**.

Species	Selected
**Arabidopsis thaliana**	y
**Brachypodium distachyon**	y
Chenopodium quinoa	n
**Chlamydomonas reinhardtii**	y
**Glycine max**	y
**Mimulus guttatus**	y
**Marchantia polymorpha**	y
**Populus trichocarpa**	y
**Setaria italica**	y
Solanum lycopersicum	n
Theobroma cacao	n
Vitis vinifera	n
**Zea mays**	y

Table S13: All plant genomes tried at any point for training and whether they were used for the final training runs. Note that various subsets and combinations were also tried.

Species	Selected
**Anabas testudineus**	y
Caenorhabditis elegans	n
**Drosophila melanogaster**	y
**Gallus gallus**	y
Homo sapiens	n
**Mus musculus**	y
**Oryzias latipes**	y
**Theropithecus gelada**	y
Xiphophorus maculatus	n

Table S14: All animal genomes tried at any point for training and whether they were used for the final training runs. Note that various subsets and combinations were also tried.

4 RNAseq Evaluation Setup

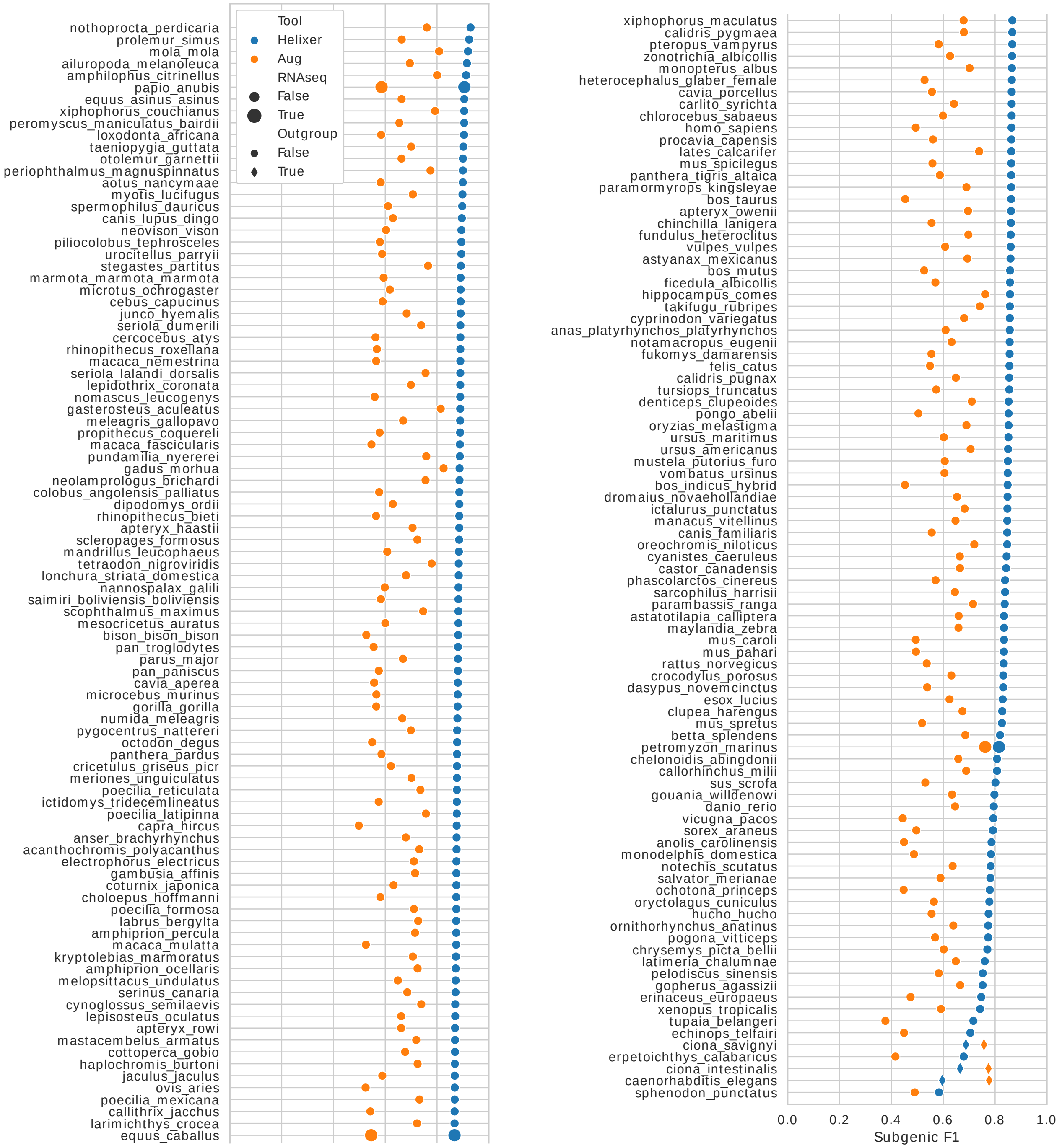

Figure S7: Visualizes how the performance of the Helixer vertebrate model (in consideration of Augustus' performance) was used to select three animal species (larger points) for detailed RNAseq analysis. The species were chosen to have good, medium and poor performance; invertebrates (diamonds) were not considered as the generalizability and recommended use of the Helixer vertebrate model currently only extends to vertebrates; candidate species were checked for the availibility of stranded, paired-end RNAseq data and skipped if none was available.

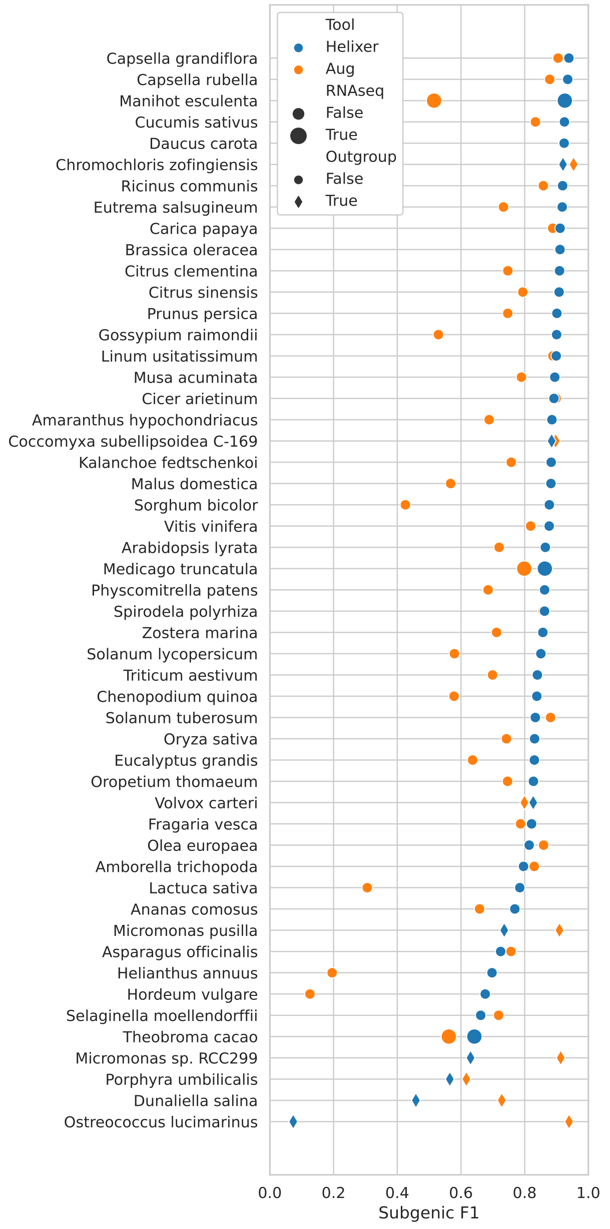

Figure S8: Visualizes how the performance of the Helixer land plant model (in consideration of Augustus' performance) was used to select three plant species (larger points) for detailed RNAseq analysis. The species were chosen to have good, medium and poor performance; algae (diamonds) were not considered as the generalizability and recommended use of the Helixer land plant model currently only extends to higher plants; candidate species were checked for the availibility of stranded, paired-end RNAseq data and skipped if none was available.

Samples	HQ	S	Samples	HQ	S	Samples	HQ	S	Samples	HQ	S
P. anubis											
SRS3122053			SRS3122094	✓	✓	SRS3270781			SRS3270847		
SRS3122057	✓	✓	SRS3122111			SRS3270785			SRS3270853		
SRS3122063	✓	✓	SRS3270734			SRS3270808			SRS3270858		
SRS3122070			SRS3270735			SRS3270811			SRS3270862		
SRS3122074			SRS3270737			SRS3270812			SRS3270863		
SRS3122079	✓	✓	SRS3270738			SRS3270821			SRS3270870		
SRS3122081			SRS3270746			SRS3270824			SRS3270872		
SRS3122088			SRS3270751			SRS3270839			SRS3270880		
SRS3122092	✓	✓	SRS3270763			SRS3270840			SRS819311	✓	✓
SRS3122093			SRS3270773			SRS3270841			SRS819317	✓	✓
E. caballus											
ERS1517592			ERS2382444			ERS2681771			SRS2552997	✓	✓
ERS1517600			ERS2681720	✓	✓	ERS2681772	✓	✓	SRS2553004	✓	✓
ERS1517608			ERS2681730			ERS2681775			SRS2770963		
ERS1517617			ERS2681733			ERS3563898			SRS2770972		
ERS1517625			ERS2681734			SRS1102326			SRS3347177		
ERS1517633			ERS2681740			SRS1102328			SRS3347182		
ERS1517641			ERS2681742			SRS1102336			SRS4826859		
ERS2382439			ERS2681750			SRS2158774			SRS4826867		
ERS2382440			ERS2681754			SRS2158777			SRS4890460	✓	✓
ERS2382442			ERS2681758			SRS2158788			SRS5330332	✓	✓
ERS2382443			ERS2681770			SRS2552988	✓	✓	SRS5330340		
P. marinus											
SRS5315360	✓	✓	SRS5315363	✓		SRS5315366	✓		SRS5315369	✓	
SRS5315361	✓	✓	SRS5315364	✓	✓	SRS5315367	✓	✓			
SRS5315362	✓	✓	SRS5315365	✓	✓	SRS5315368	✓	✓			
M. esculenta											
SRS1608507			SRS1610089	✓	✓	SRS1610201			SRS946172		
SRS1608510	✓	✓	SRS1610128	✓	✓	SRS1610205	✓	✓	SRS946227		
SRS1610036			SRS1610150			SRS1610207			SRS946633		
SRS1610041			SRS1610177			SRS1610208			SRS946650		
SRS1610076	✓	✓	SRS1610181			SRS945853			SRS946703		
SRS1610079	✓	✓	SRS1610187			SRS946078			SRS947897		
SRS1610084	✓	✓	SRS1610197			SRS946138					
M. truncatula											
ERS1556003			SRS1948297			SRS3700721	✓		SRS4425365	✓	
ERS1556006			SRS1948300			SRS3700725	✓	✓	SRS4538246		
ERS1556009			SRS1948304	✓		SRS4425341			SRS4538249		
ERS1556012			SRS1948306			SRS4425343	✓		SRS5045319		
ERS1556015			SRS1948309			SRS4425346	✓	✓	SRS5045322		
ERS1556018	✓		SRS1948313	✓	✓	SRS4425347	✓	✓	SRS5045325		
ERS1556021			SRS1948315			SRS4425350	✓		SRS5045328		
ERS1556024			SRS3700712	✓		SRS4425353			SRS5045330		
SRS1736395	✓	✓	SRS3700715	✓		SRS4425356	✓	✓	SRS5045334		
SRS1736398	✓		SRS3700717			SRS4425357			SRS5045337		
SRS1948293			SRS3700718	✓	✓	SRS4425364	✓				
T. cacao											
SRS5244538	✓		SRS5244546	✓	✓	SRS5244554			SRS5244562	✓	✓
SRS5244539			SRS5244547			SRS5244555			SRS5244563		
SRS5244540			SRS5244548			SRS5244556	✓	✓	SRS5244564		
SRS5244541			SRS5244549			SRS5244557	✓		SRS5244565	✓	✓
SRS5244542			SRS5244550			SRS5244558			SRS5244566		
SRS5244543	✓	✓	SRS5244551			SRS5244559			SRS5244567		
SRS5244544	✓	✓	SRS5244552			SRS5244560			SRS5244568		
SRS5244545			SRS5244553	✓	✓	SRS5244561					

Table S15: List of RNAseq samples that were selected for processing, whether they were of high quality (HQ), and finally, whether they were ultimately selected (S) for merging and use in coverage calculation.

Purpose	Tool	Version	Customized Parameters
Read quality control	FastQC	v0.11.5	
Adapter, Quality trimming	Trimmomatic	0.36	ILLUMINACLIP:TruSeq3-PE-2.fa:3:30:10:1:true MAXINFO:36:0.7 MINLEN:36
Mapping	Hisat2	2.1.0	Both: –max-seeds 8 –dta<br>Plants only: –pen-canintronlen G,-8,1.5 –pen-noncanintronlen G,-8,1.5
Bam conversion, sorting and indexing	Samtools	1.6	
Mapping quality control	PicardTools	52.0	CollectRnaSeqMetrics STRAND=SECOND_READ_TRANSCRIPTION_STRAND
Summarizing and organizing	MultiQC	1.8	

Table S16: List of bioinformatics software, versions, and parameters used in processing public RNAseq data.

5 Longer Sequence Input

Class	Input Length in bp
Mammalia	200.000
Non-avian Reptilia	200.000
Aves	100.000
Amphibia	100.000
Actinopterygii	50.000
Chondrichthyes	50.000
Actinistia	50.000
Ascidiacea	20.000
Insecta	20.000

Table S17: Input sequence length for each phylogenetic class present in our animal dataset. As a rule of thumb, we found the input length should be proportional to the average gene length of the class while staying in the interval [20.000, 200.000]. If the N75 of a specific species was less than twice as high as the supposed sequence length it was lowered until either this criteria as met or a length of 50.000 was reached.

6 Effect of Overlapping by Species

The following plots show the effect of overlapping for each species we worked with individually. The plots are ordered with descending N75, as we found that overlapping tends to produce less desirable patterns in the prediction quality for the very fragmented genomes.

6.1 Animal Data, Vertebrate Model

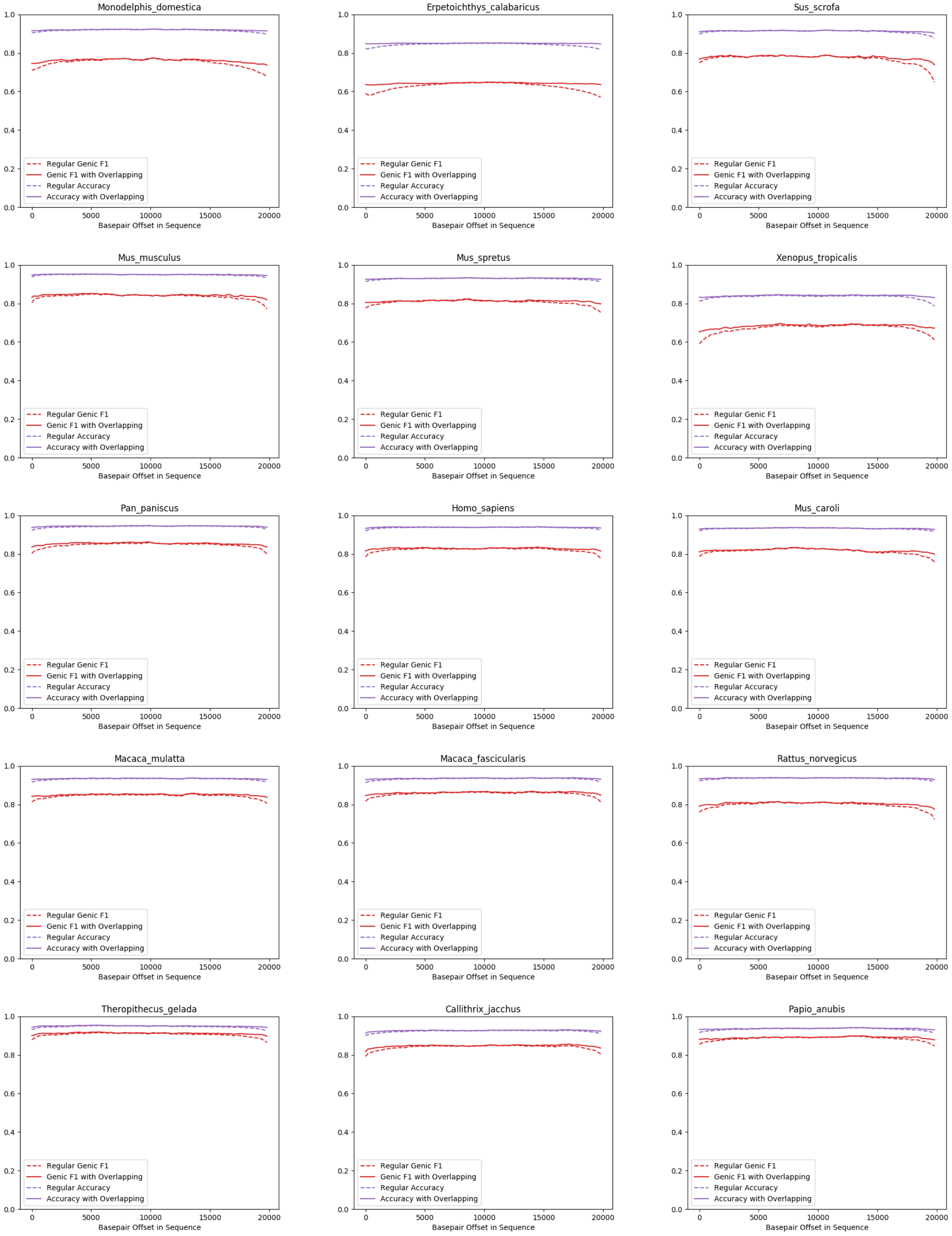
Monodelphis_domestica
Erpetoichthys_calabaricus
Sus_scrofa
Mus_musculus
Mus_spretus
Xenopus_tropicalis
Pan_paniscus
Homo_sapiens
Mus_caroli
Macaca_mulatta
Macaca_fascicularis
Rattus_norvegicus
Theropithecus_gelada
Callithrix_jacchus
Papio_anubis
Regular Genic F1
Genic F1 with Overlapping
Regular Accuracy
Accuracy with Overlapping
Basepair Offset in Sequence
0.0
0.2
0.4
0.6
0.8
1.0
0
5000
10000
15000
20000

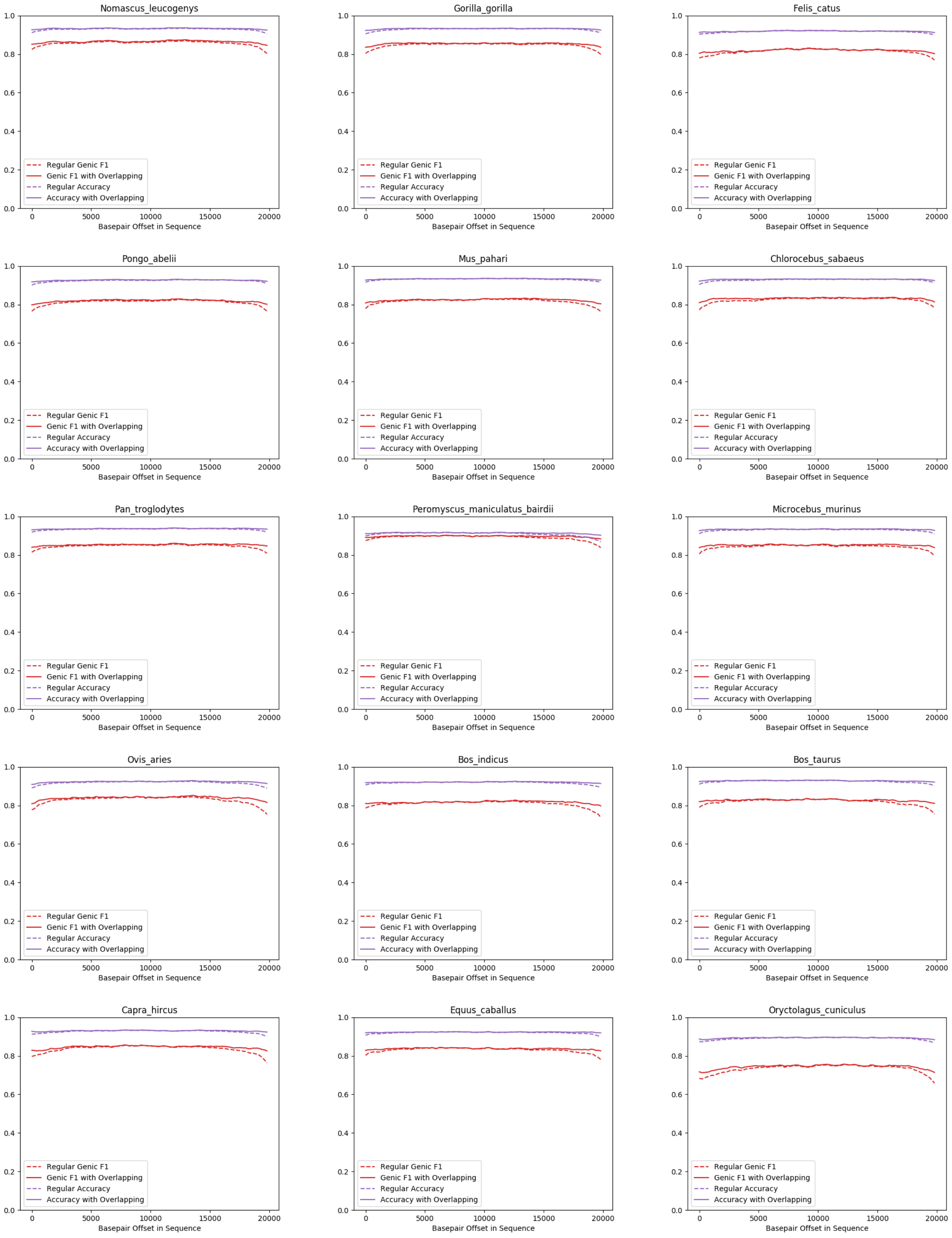
Nomascus_leucogenys
Gorilla_gorilla
Felis_catus
Pongo_abelii
Mus_pahari
Chlorocebus_sabaeus
Pan_troglodytes
Peromyscus_maniculatus_bairdii
Microcebus_murinus
Ovis_aries
Bos_indicus
Bos_taurus
Capra_hircus
Equus_caballus
Oryctolagus_cuniculus
Regular Genic F1
Genic F1 with Overlapping
Regular Accuracy
Accuracy with Overlapping
Basepair Offset in Sequence
0.0
0.2
0.4
0.6
0.8
1.0
0
5000
10000
15000
20000

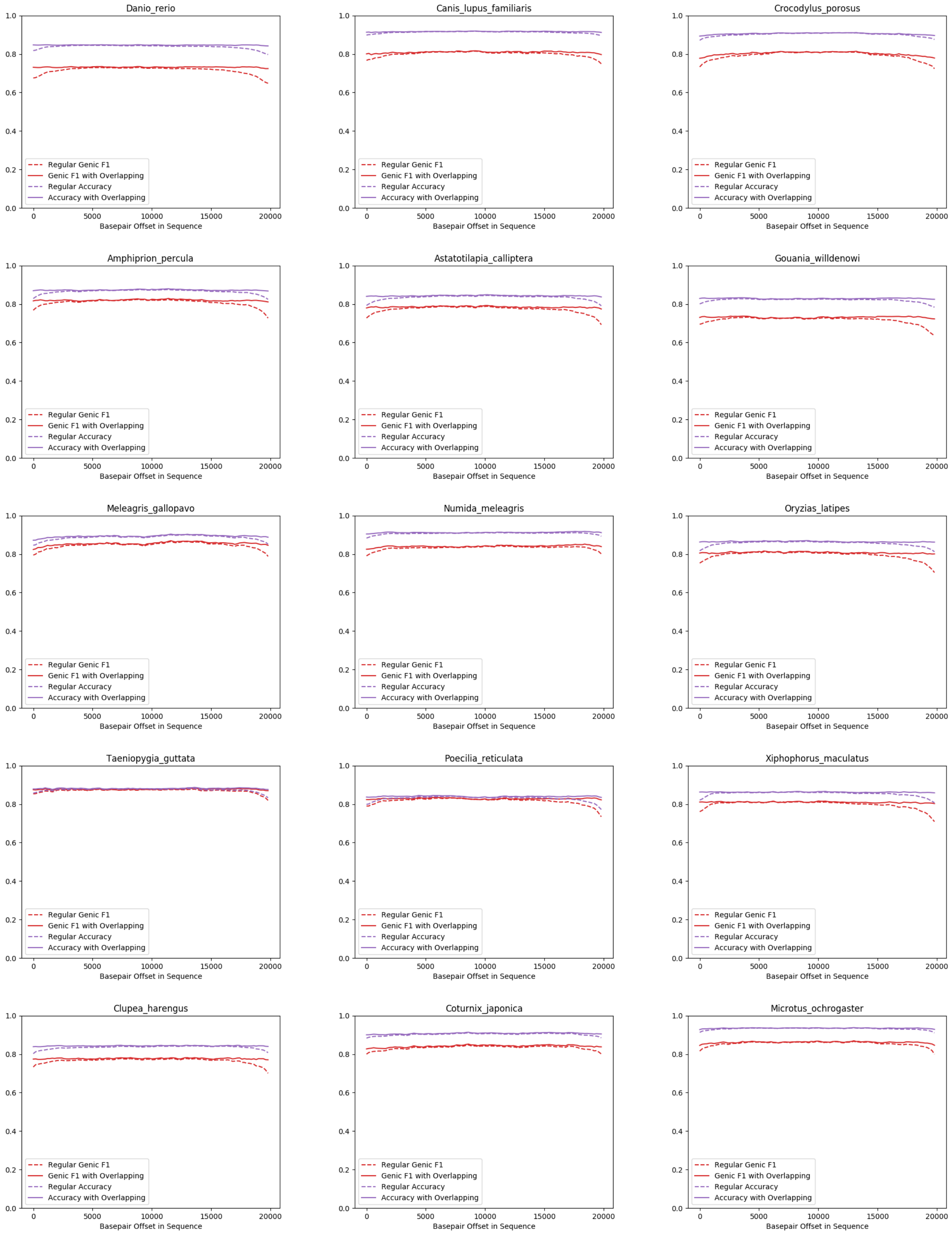
Danio_rerio
Canis_lupus_familiaris
Crocodylus_porosus
Amphiprion_percula
Astatotilapia_calliptera
Gouania_willdenowi
Meleagris_gallopavo
Numida_meleagris
Oryzias_latipes
Taeniopygia_guttata
Poecilia_reticulata
Xiphophorus_maculatus
Clupea_harengus
Coturnix_japonica
Microtus_ochrogaster
Regular Genic F1
Genic F1 with Overlapping
Regular Accuracy
Accuracy with Overlapping
Basepair Offset in Sequence

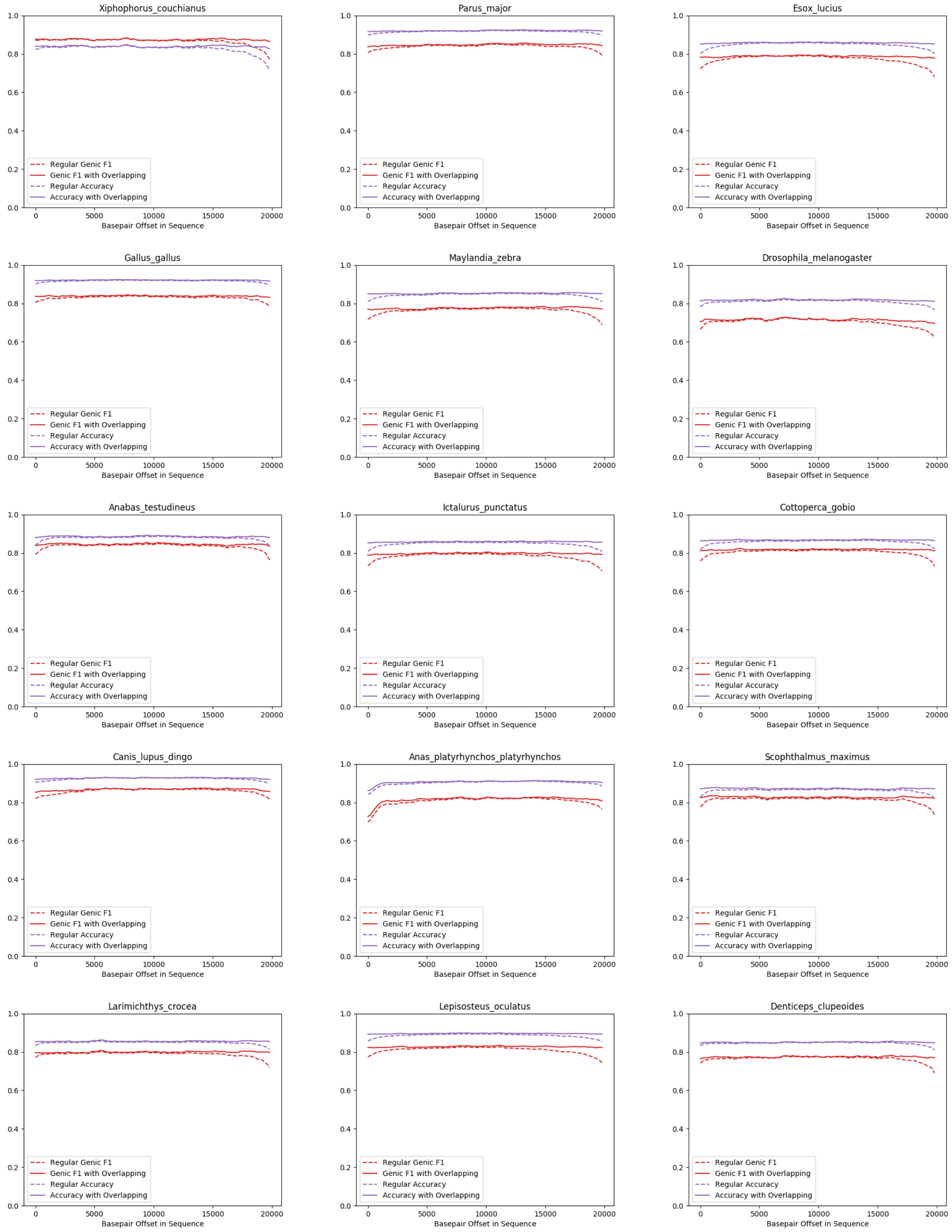
Xiphophorus_couchianus
Parus_major
Esox_lucius
Gallus_gallus
Maylandia_zebra
Drosophila_melanogaster
Anabas_testudineus
Ictalurus_punctatus
Cottoperca_gobio
Canis_lupus_dingo
Anas_platyrhynchos_platyrhynchos
Scophthalmus_maximus
Larimichthys_crocea
Lepisosteus_oculatus
Denticeps_clupeoides
Regular Genic F1
Genic F1 with Overlapping
Regular Accuracy
Accuracy with Overlapping
Basepair Offset in Sequence
0 5000 10000 15000 20000
0.0 0.2 0.4 0.6 0.8 1.0

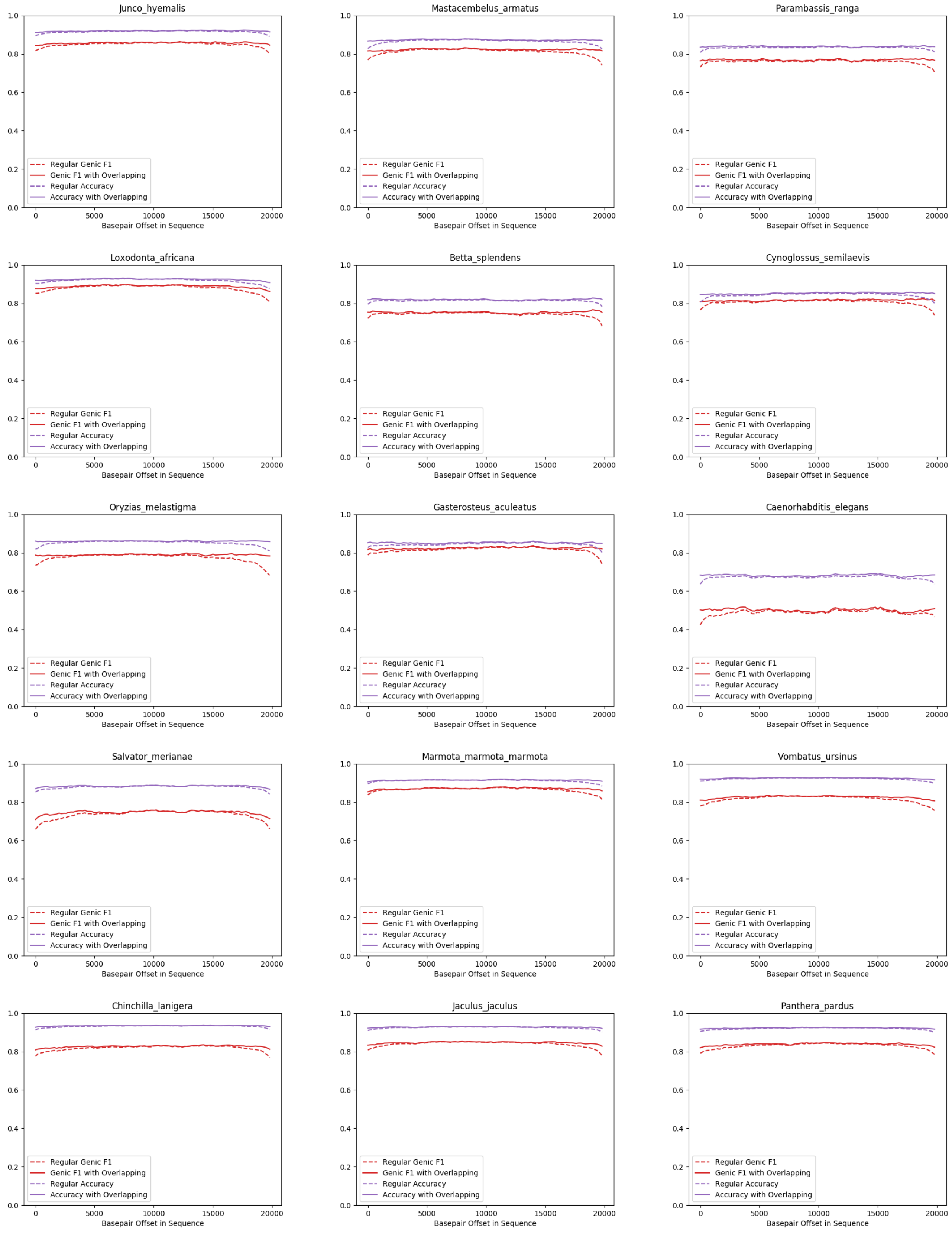
Junco_hyemalis
Mastacembelus_armatus
Parambassis_ranga
Loxodonta_africana
Betta_splendens
Cynoglossus_semilaevis
Oryzias_melastigma
Gasterosteus_aculeatus
Caenorhabditis_elegans
Salvator_merianae
Marmota_marmota_marmota
Vombatus_ursinus
Chinchilla_lanigera
Jaculus_jaculus
Panthera_pardus
Regular Genic F1
Genic F1 with Overlapping
Regular Accuracy
Accuracy with Overlapping
Basepair Offset in Sequence

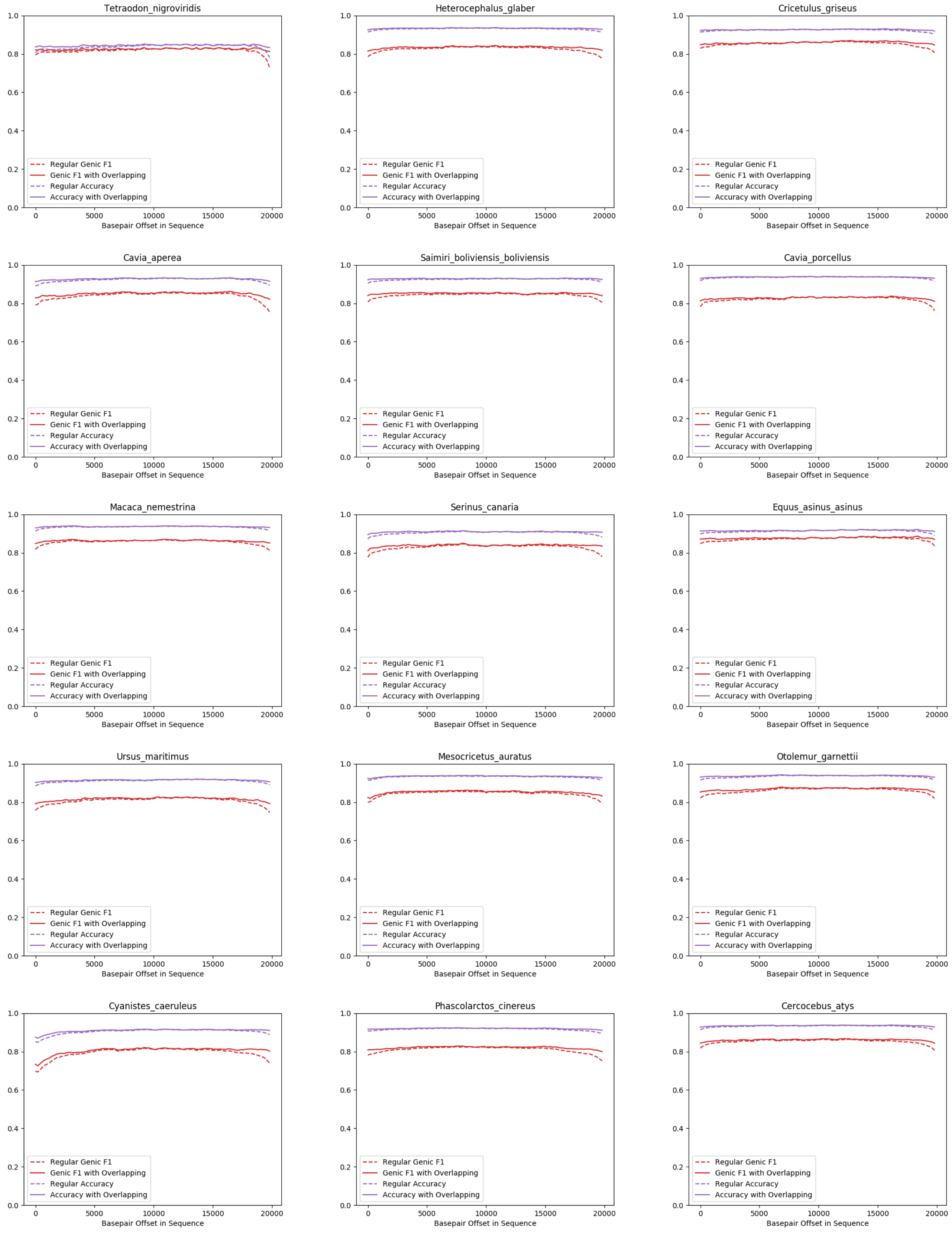
Tetraodon_nigroviridis
Heterocephalus_glaber
Cricetulus_griseus
Cavia_aperea
Saimiri_boliviensis_boliviensis
Cavia_porcellus
Macaca_nemestrina
Serinus_canaria
Equus_asinus_asinus
Ursus_maritimus
Mesocricetus_auratus
Otolemur_garnettii
Cyanistes_caeruleus
Phascolarctos_cinereus
Cercocebus_atys
Regular Genic F1
Genic F1 with Overlapping
Regular Accuracy
Accuracy with Overlapping
Basepair Offset in Sequence
0.0
0.2
0.4
0.6
0.8
1.0
0
5000
10000
15000
20000

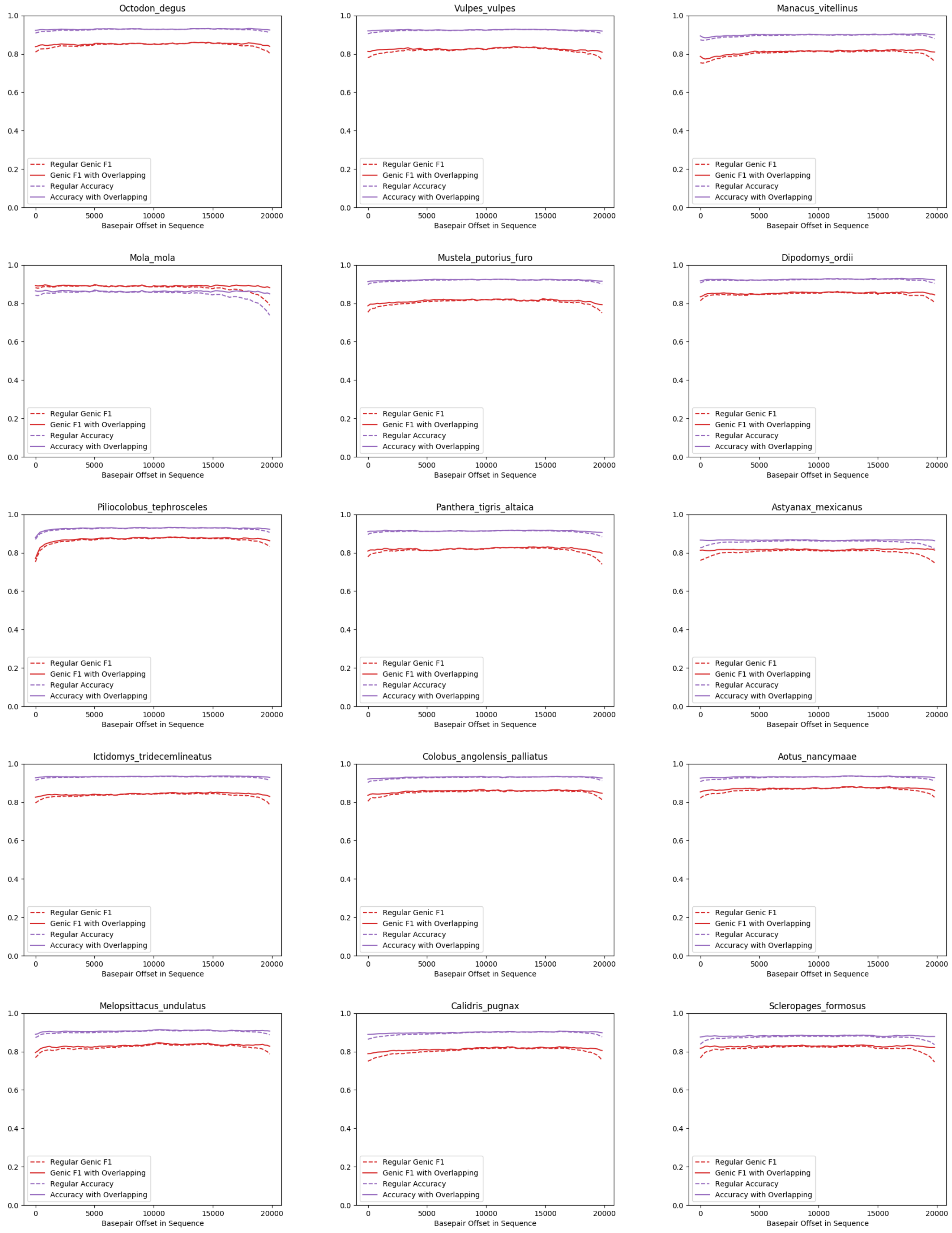
Octodon_degus
Vulpes_vulpes
Manacus_vitellinus
Mola_mola
Mustela_putorius_furo
Dipodomys_ordii
Piliocolobus_tephrosceles
Panthera_tigris_altaica
Astyanax_mexicanus
Ictidomys_tridecemlineatus
Colobus_angolensis_palliatus
Aotus_nancymaae
Melopsittacus_undulatus
Calidris_pugnax
Scleropages_formosus
Regular Genic F1
Genic F1 with Overlapping
Regular Accuracy
Accuracy with Overlapping
Basepair Offset in Sequence

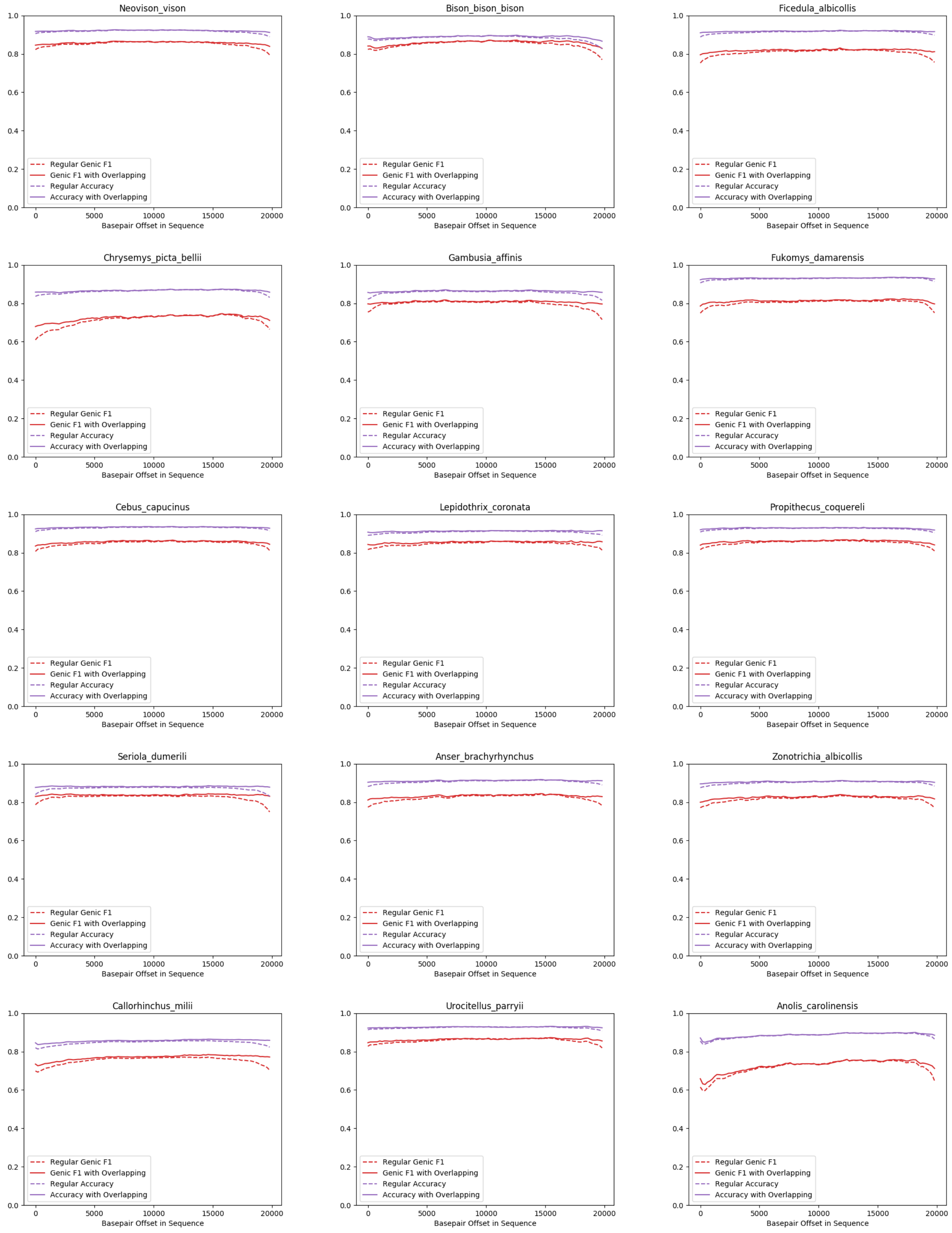
Neovison_vison
Bison_bison_bison
Ficedula_albicollis
Chrysemys_picta_bellii
Gambusia_affinis
Fukomys_damarensis
Cebus_capucinus
Lepidothrix_coronata
Propithecus_coquereli
Seriola_dumerili
Anser_brachyrhynchus
Zonotrichia_albicollis
Callorhinchus_milii
Urocitellus_parryii
Anolis_carolinensis
Regular Genic F1
Genic F1 with Overlapping
Regular Accuracy
Accuracy with Overlapping
Basepair Offset in Sequence

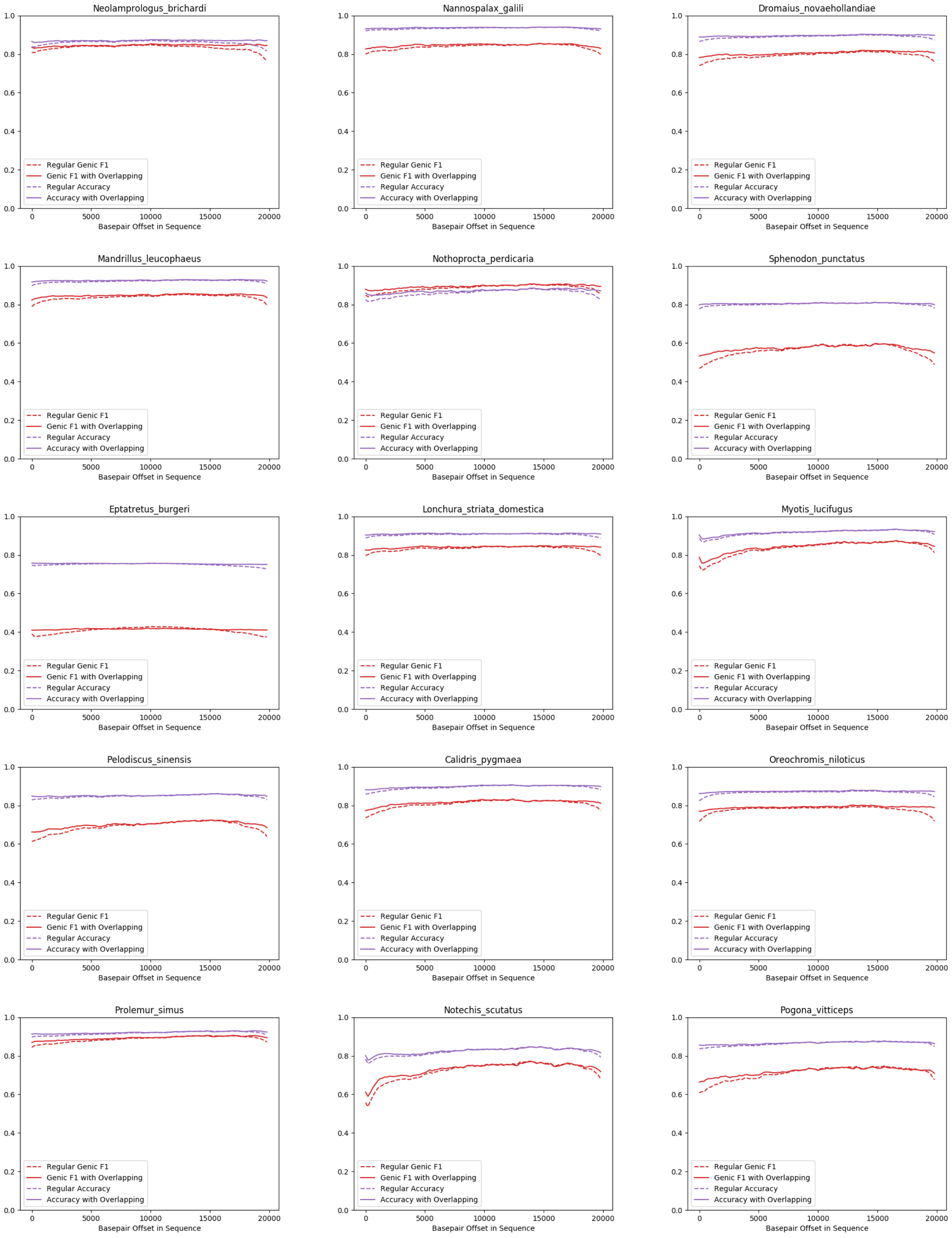
Neolamprologus_brichardi
Nannospalax_galili
Dromaius_novaehollandiae
Mandrillus_leucophaeus
Nothoprocta_perdicaria
Sphenodon_punctatus
Eptatretus_burgeri
Lonchura_striata_domestica
Myotis_lucifugus
Pelodiscus_sinensis
Calidris_pygmaea
Oreochromis_niloticus
Prolemur_simus
Notechis_scutatus
Pogona_vitticeps
Regular Genic F1
Genic F1 with Overlapping
Regular Accuracy
Accuracy with Overlapping
Basepair Offset in Sequence

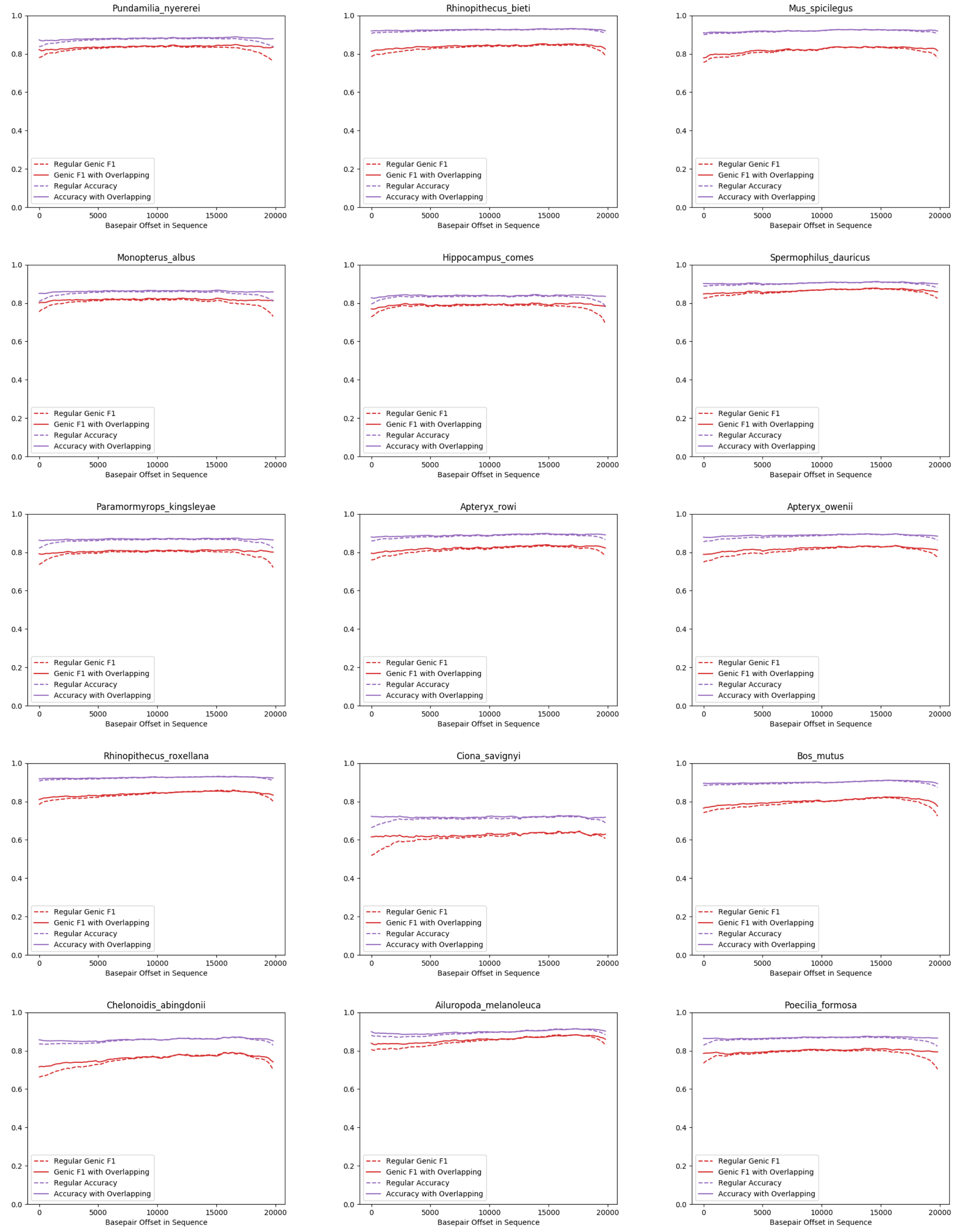
Pundamilia_nyererei
Rhinopithecus_bieti
Mus_spicilegus
Monopterus_albus
Hippocampus_comes
Spermophilus_dauricus
Paramormyrops_kingsleyae
Apteryx_rowi
Apteryx_owenii
Rhinopithecus_roxellana
Ciona_savignyi
Bos_mutus
Chelonoidis_abingdonii
Ailuropoda_melanoleuca
Poecilia_formosa
Regular Genic F1
Genic F1 with Overlapping
Regular Accuracy
Accuracy with Overlapping
Basepair Offset in Sequence

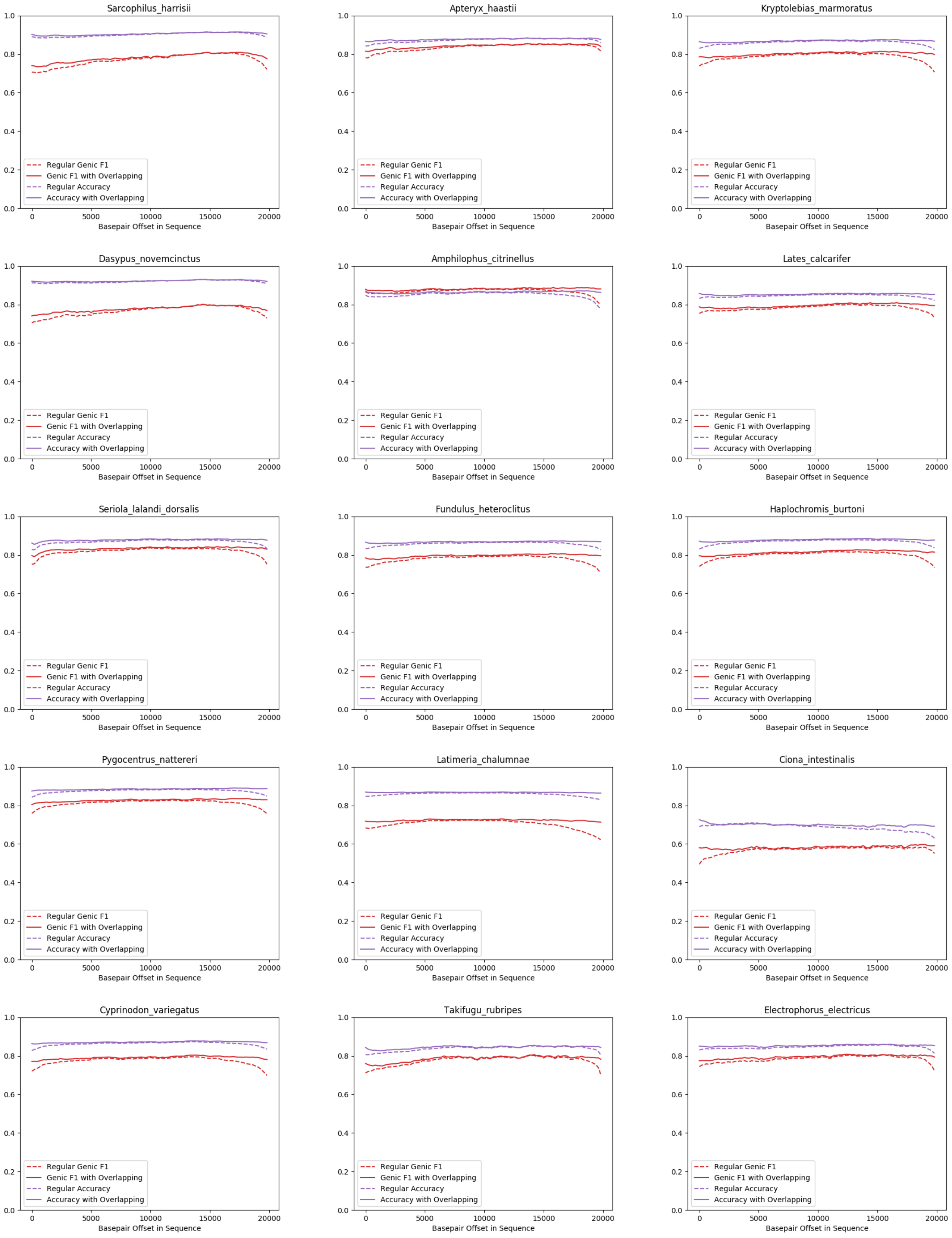
Sarcophilus_harrisii
Apteryx_haastii
Kryptolebias_marmoratus
Dasypus_novemcinctus
Amphilophus_citrinellus
Lates_calcarifer
Seriola_lalandi_dorsalis
Fundulus_heteroclitus
Haplochromis_burtoni
Pygocentrus_nattereri
Latimeria_chalumnae
Ciona_intestinalis
Cyprinodon_variegatus
Takifugu_rubripes
Electrophorus_electricus
Regular Genic F1
Genic F1 with Overlapping
Regular Accuracy
Accuracy with Overlapping
Basepair Offset in Sequence
0.0
0.2
0.4
0.6
0.8
1.0
0
5000
10000
15000
20000

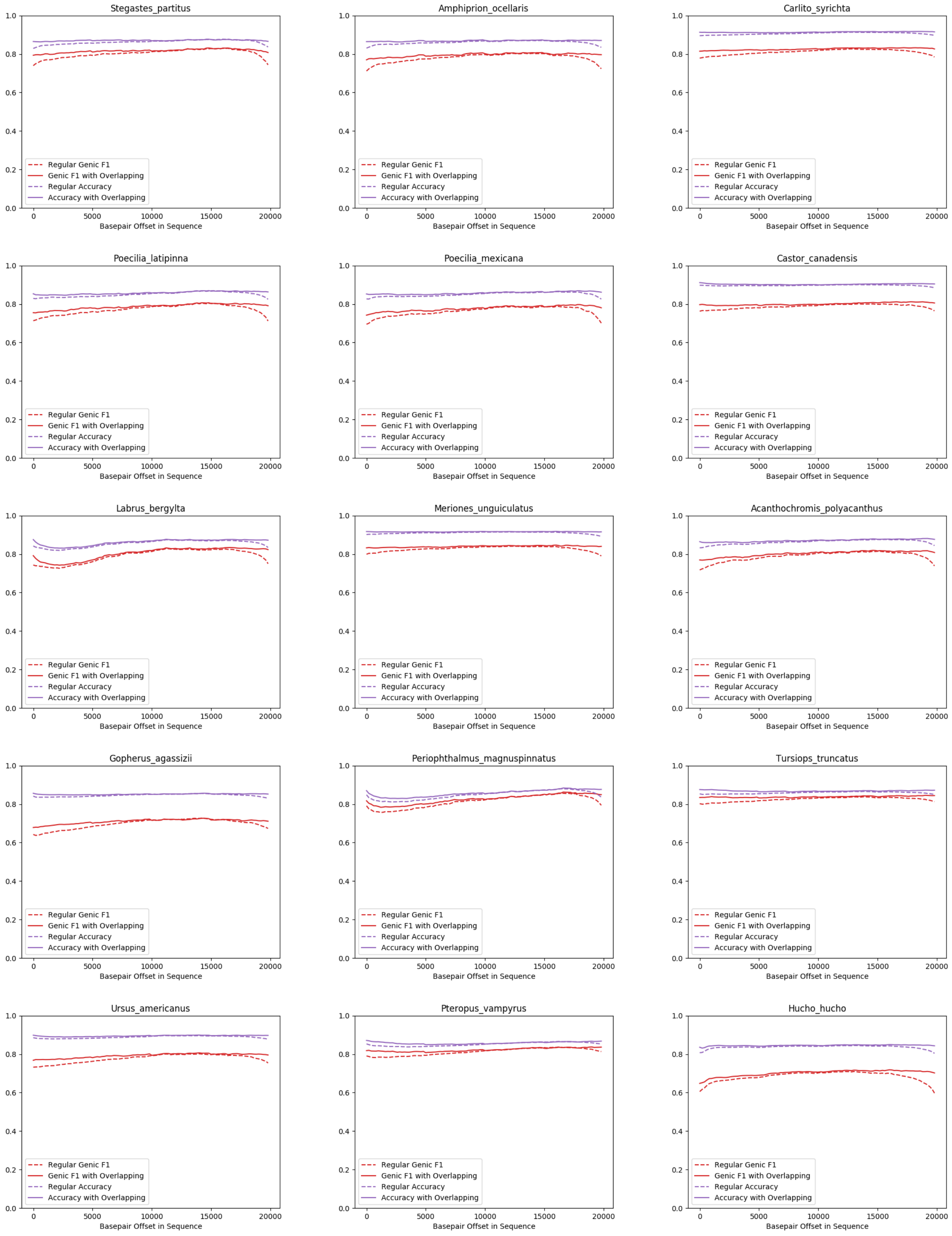
Stegastes_partitus
Regular Genic F1
Genic F1 with Overlapping
Regular Accuracy
Accuracy with Overlapping
Basepair Offset in Sequence
Amphiprion_ocellaris
Regular Genic F1
Genic F1 with Overlapping
Regular Accuracy
Accuracy with Overlapping
Basepair Offset in Sequence
Carlito_syrichta
Regular Genic F1
Genic F1 with Overlapping
Regular Accuracy
Accuracy with Overlapping
Basepair Offset in Sequence
Poecilia_latipinna
Regular Genic F1
Genic F1 with Overlapping
Regular Accuracy
Accuracy with Overlapping
Basepair Offset in Sequence
Poecilia_mexicana
Regular Genic F1
Genic F1 with Overlapping
Regular Accuracy
Accuracy with Overlapping
Basepair Offset in Sequence
Castor_canadensis
Regular Genic F1
Genic F1 with Overlapping
Regular Accuracy
Accuracy with Overlapping
Basepair Offset in Sequence
Labrus_bergylta
Regular Genic F1
Genic F1 with Overlapping
Regular Accuracy
Accuracy with Overlapping
Basepair Offset in Sequence
Meriones_unguiculatus
Regular Genic F1
Genic F1 with Overlapping
Regular Accuracy
Accuracy with Overlapping
Basepair Offset in Sequence
Acanthochromis_polyacanthus
Regular Genic F1
Genic F1 with Overlapping
Regular Accuracy
Accuracy with Overlapping
Basepair Offset in Sequence
Gopherus_agassizii
Regular Genic F1
Genic F1 with Overlapping
Regular Accuracy
Accuracy with Overlapping
Basepair Offset in Sequence
Periophthalmus_magnuspinnatus
Regular Genic F1
Genic F1 with Overlapping
Regular Accuracy
Accuracy with Overlapping
Basepair Offset in Sequence
Tursiops_truncatus
Regular Genic F1
Genic F1 with Overlapping
Regular Accuracy
Accuracy with Overlapping
Basepair Offset in Sequence
Ursus_americanus
Regular Genic F1
Genic F1 with Overlapping
Regular Accuracy
Accuracy with Overlapping
Basepair Offset in Sequence
Pteropus_vampyrus
Regular Genic F1
Genic F1 with Overlapping
Regular Accuracy
Accuracy with Overlapping
Basepair Offset in Sequence
Hucho_hucho
Regular Genic F1
Genic F1 with Overlapping
Regular Accuracy
Accuracy with Overlapping
Basepair Offset in Sequence

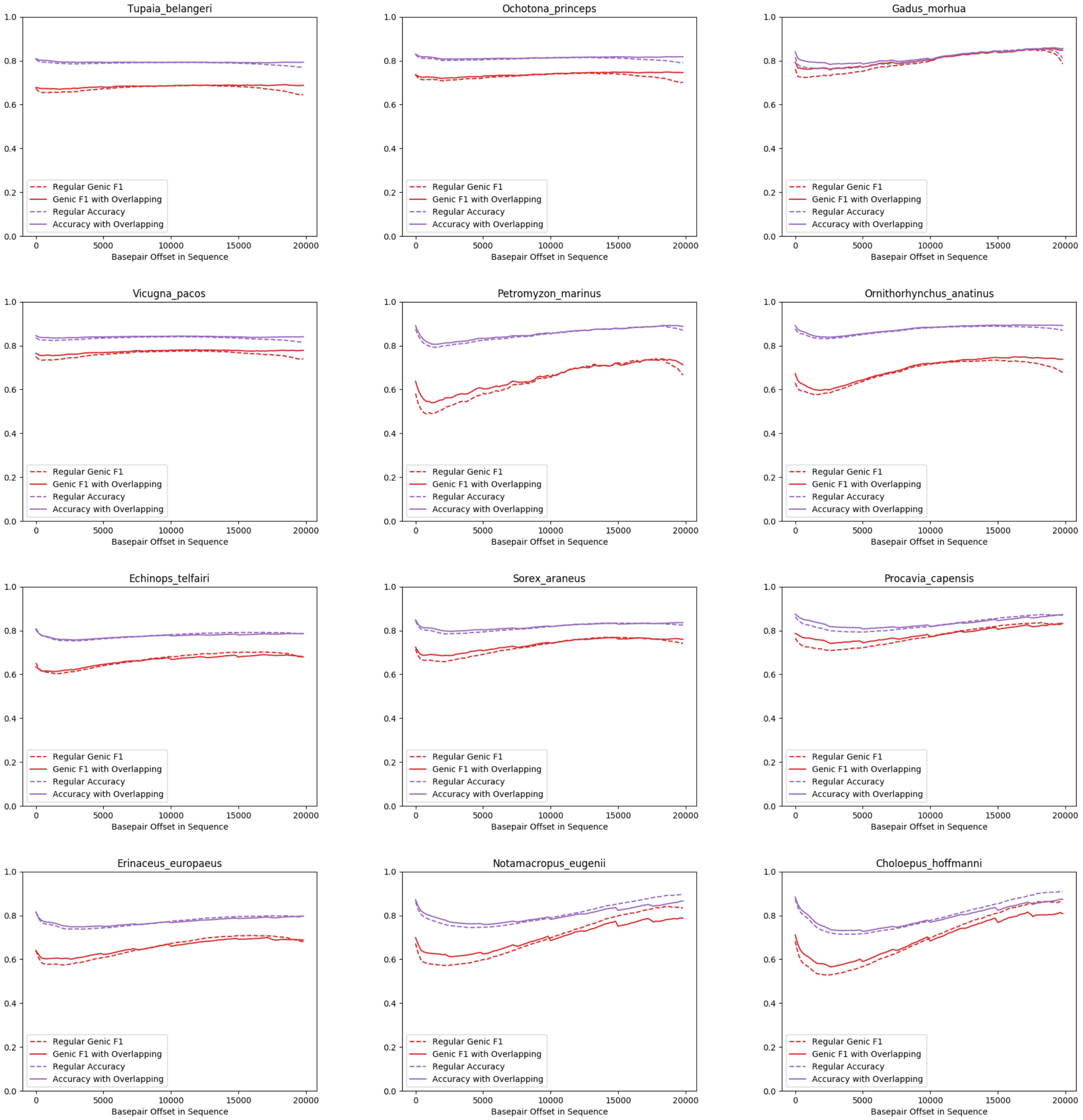

Figure S9: Effect of overlapping on the sequence positions wise bias ordered by descending N75 for all animals genomes individually.

6.2 Plant Data, Land Plant Model

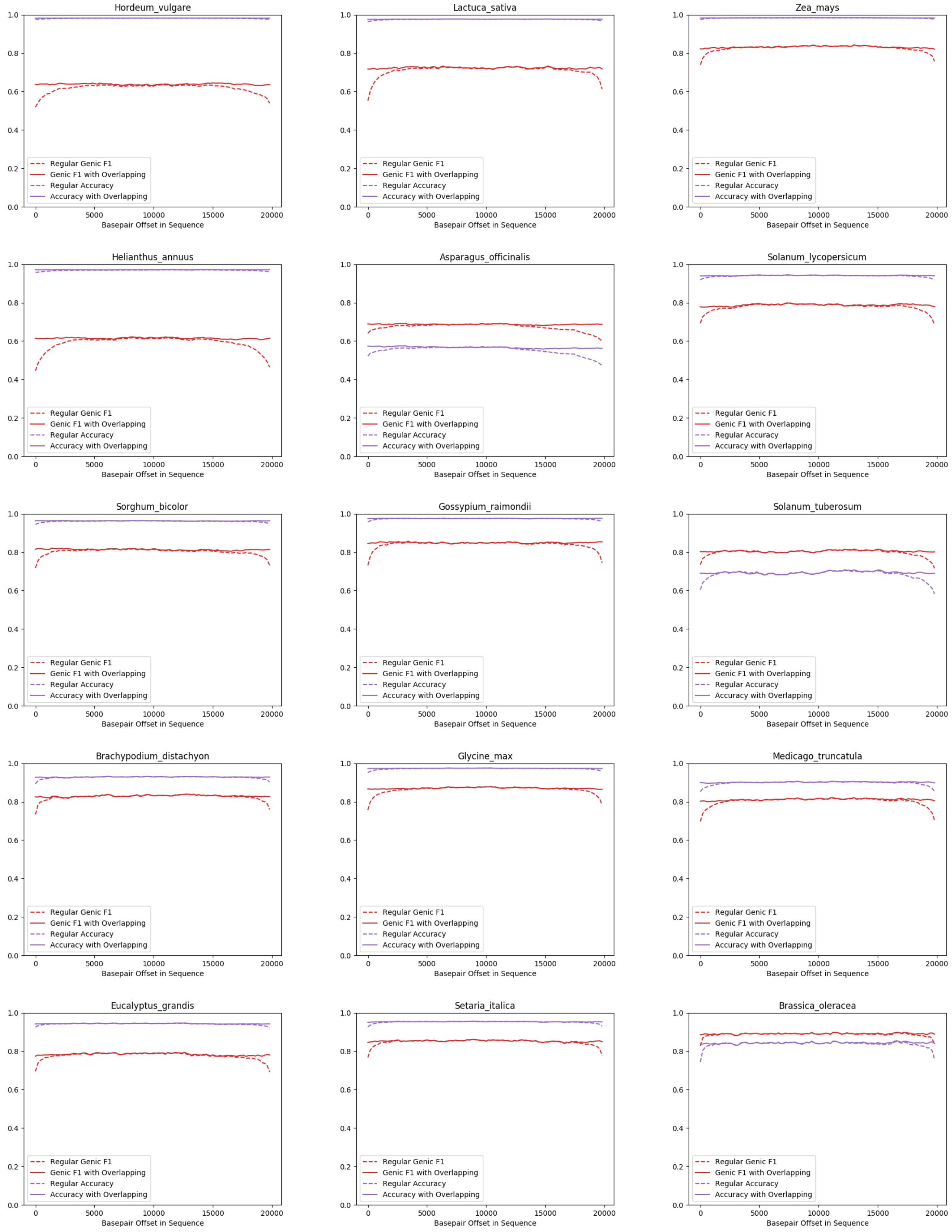

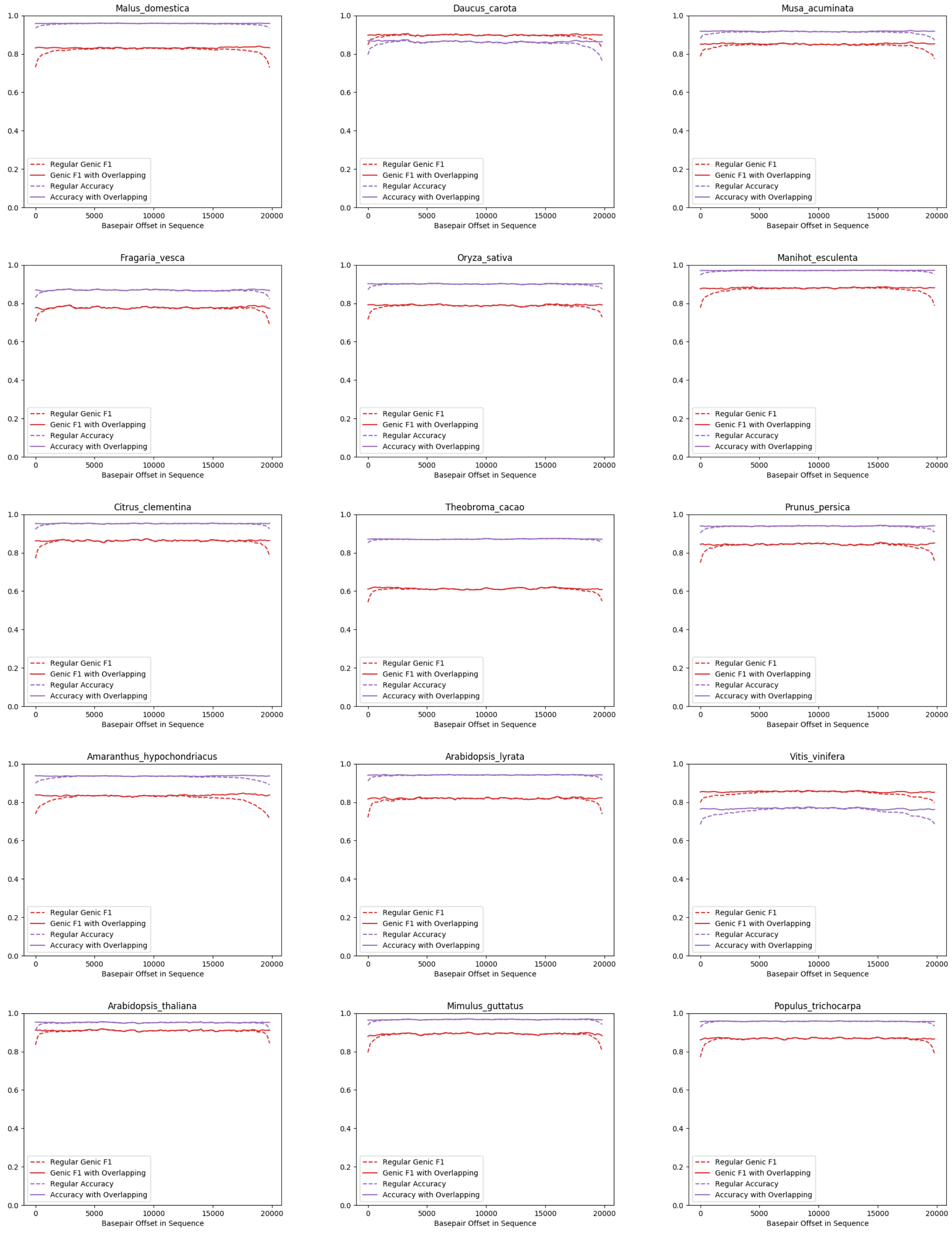
Malus_domestica
Daucus_carota
Musa_acuminata
Fragaria_vesca
Oryza_sativa
Manihot_esculenta
Citrus_clementina
Theobroma_cacao
Prunus_persica
Amaranthus_hypochondriacus
Arabidopsis_lyrata
Vitis_vinifera
Arabidopsis_thaliana
Mimulus_guttatus
Populus_trichocarpa
Regular Genic F1
Genic F1 with Overlapping
Regular Accuracy
Accuracy with Overlapping
Basepair Offset in Sequence

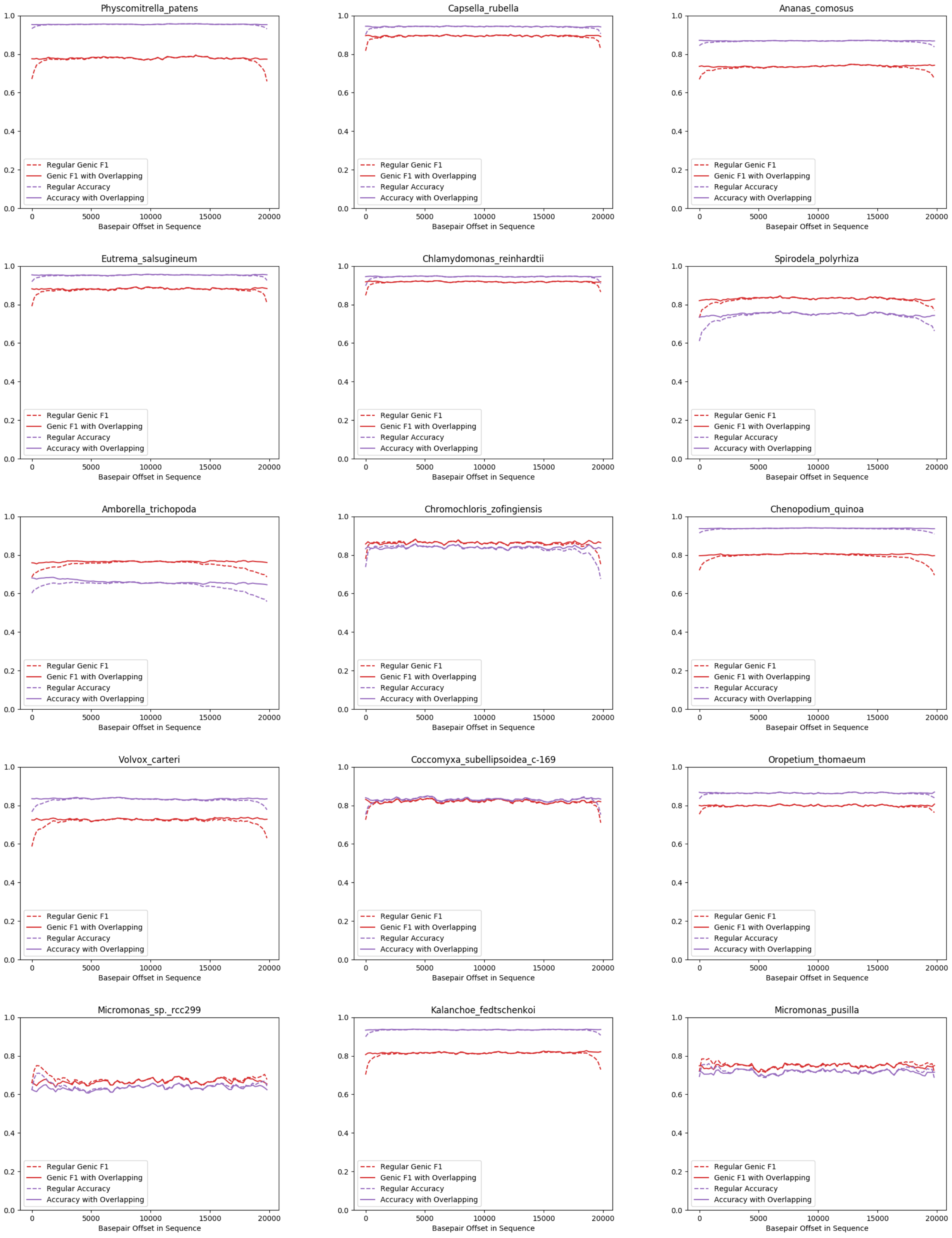
Physcomitrella_patens
Capsella_rubella
Ananas_comosus
Eutrema_salsugineum
Chlamydomonas_reinhardtii
Spirodela_polyrhiza
Amborella_trichopoda
Chromochloris_zofingiensis
Chenopodium_quinoa
Volvox_carteri
Coccomyxa_subellipsoidea_c-169
Oropetium_thomaeum
Micromonas_sp._rcc299
Kalanchoe_fedtschenkoi
Micromonas_pusilla
Regular Genic F1
Genic F1 with Overlapping
Regular Accuracy
Accuracy with Overlapping
Basepair Offset in Sequence
0.0
0.2
0.4
0.6
0.8
1.0
0
5000
10000
15000
20000

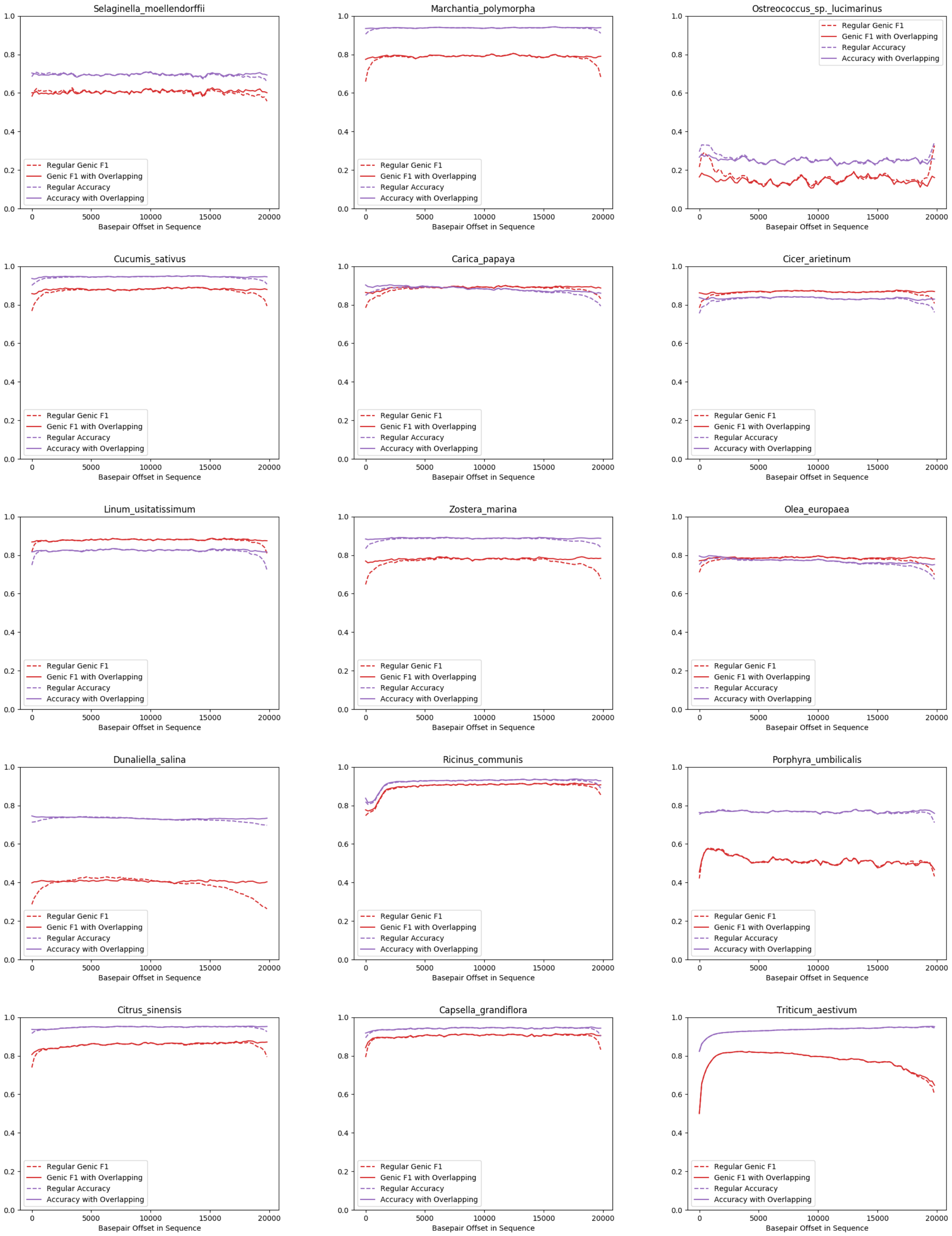

Figure S10: Effect of overlapping on the sequence positions wise bias ordered by descending N75 for all plant genomes individually.

7 Training Data statistics

	Animals	Plants
Average genome size in Gbp	2.489 (+- 2.073)	0.914 (+- 0.934)
Average gene length	25,672 (+- 16,605)	3,509 (+- 906)
Geenuff error rate	0.253 (+- 0.138)	0.134 (+- 0.072)
Fraction of class Intergenic	0.752 (+- 0.05)	0.808 (+- 0.106)
Fraction of class UTR	0.013 (+- 0.011)	0.033 (+- 0.023)
Fraction of class CDS	0.028 (+- 0.028)	0.077 (+- 0.052)
Fraction of class Intron	0.207 (+- 0.023)	0.083 (+- 0.037)

Table S18: The description of Table 2 applies here as well.

8 Detailed results

Generalizable group	file name	training run	epoch	genic Precision	genic Recall	genic F1
Vertebrates	animals_a_e07.h5	animals_a	7	0.7932	0.7189	0.7542
Vertebrates	animals_a_e08.h5	animals_a	8	0.8342	0.6498	0.7305
Vertebrates	animals_b_e09.h5	animals_b	9	0.7687	0.7331	0.7505
Vertebrates	animals_b_e06.h5	animals_b	6	0.8219	0.6728	0.7399
Vertebrates	animals_c_e09.h5	animals_c	9	0.835	0.6805	0.7498
Vertebrates	animals_c_e07.h5	animals_c	7	0.799	0.7024	0.7476
Vertebrates	animals_d_e08.h5	animals_d	8	0.809	0.693	0.7466
Vertebrates	animals_d_e06.h5	animals_d	6	0.837	0.6175	0.7107
Mean				0.812	0.684	0.741
Standard deviation				0.024	0.037	0.014
Land plants	plants_a_e10.h5	plants_a	10	0.8645	0.8145	0.8387
Land plants	plants_a_e07.h5	plants_a	7	0.8192	0.8257	0.8224
Land plants	plants_b_e11.h5	plants_b	11	0.8349	0.8178	0.8263
Land plants	plants_b_e12.h5	plants_b	12	0.8017	0.8328	0.817
Land plants	plants_c_e11.h5	plants_c	11	0.8513	0.8124	0.8314
Land plants	plants_c_e07.h5	plants_c	7	0.7978	0.8275	0.8124
Land plants	plants_d_e10.h5	plants_d	10	0.8358	0.8268	0.8313
Land plants	plants_d_e12.h5	plants_d	12	0.8059	0.8398	0.8225
Mean				0.826	0.825	0.825
Standard deviation				0.024	0.009	0.008

Table S19: Individual model checkpoints that were selected for the final ensemble and their performance on the validation set of the training genomes. Files are available at https://zenodo.org/record/3974409

9 Evaluation against RNAseq

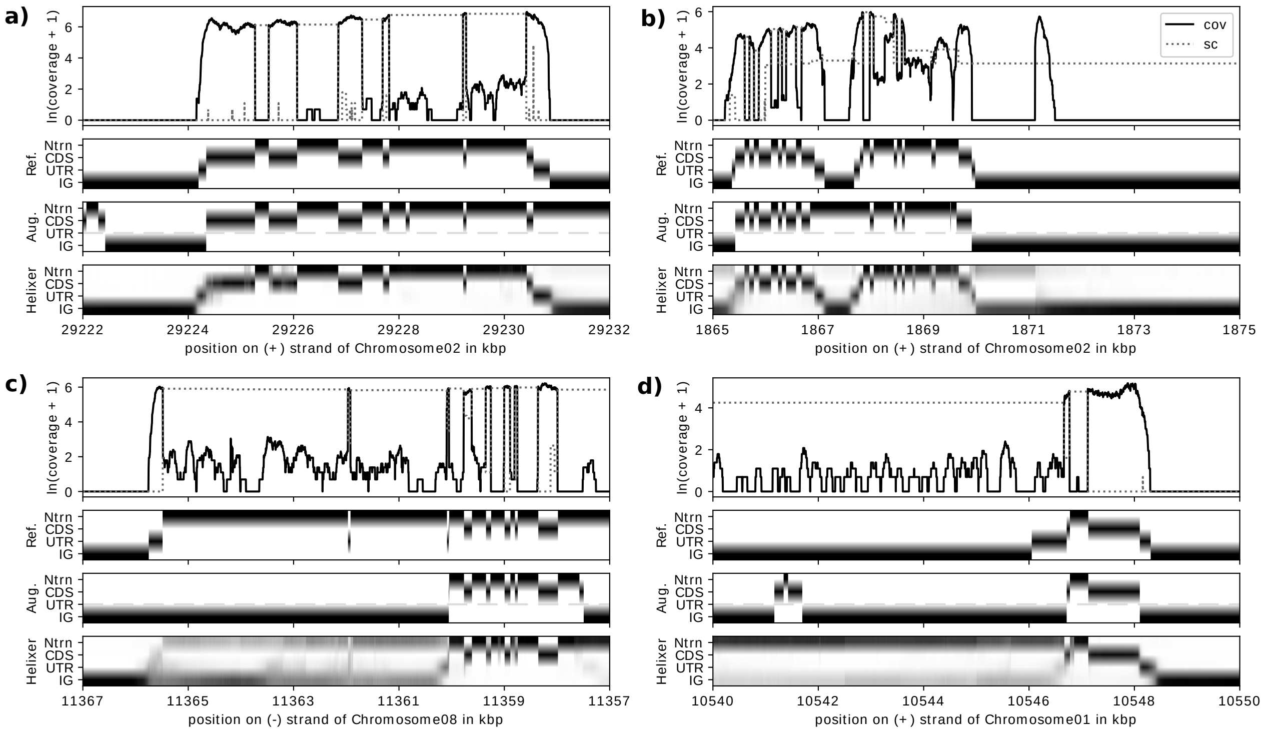

Figure S11: Four additional example helixer predictions in the context of RNAseq data, the reference, and Augustus' prediction for *M. esculenta*. The examples were chosen so that a) Helixer had high accuracy against the reference and the reference was supported by the RNAseq data, b) Helixer had high accuracy but the reference was not supported by the RNAseq data, c) Helixer had low accuracy and the reference was supported by the RNAseq data, and d) helixer had low accuracy but the reference was not supported by RNAseq data. Feasibility for visualization was a major secondary consideration. Each subplot shows from top to bottom i) the natural log of the coverage ("cov", solid) and spliced coverage ("sc", dotted) + 1, ii) the reference annotation in matrix form, iii) Augustus' predictions in matrix form (the dashed line is a reminder that no UTR predictions are expected), and iv) Helixer's predictions. The reference and Augustus have either 0 (white) or 1 (black) for each base pair and category, while Helixer emits a probability from 0-1 represented via gray-scale. "Ntrn" stand for intron, and "IG" stands for intergenic.

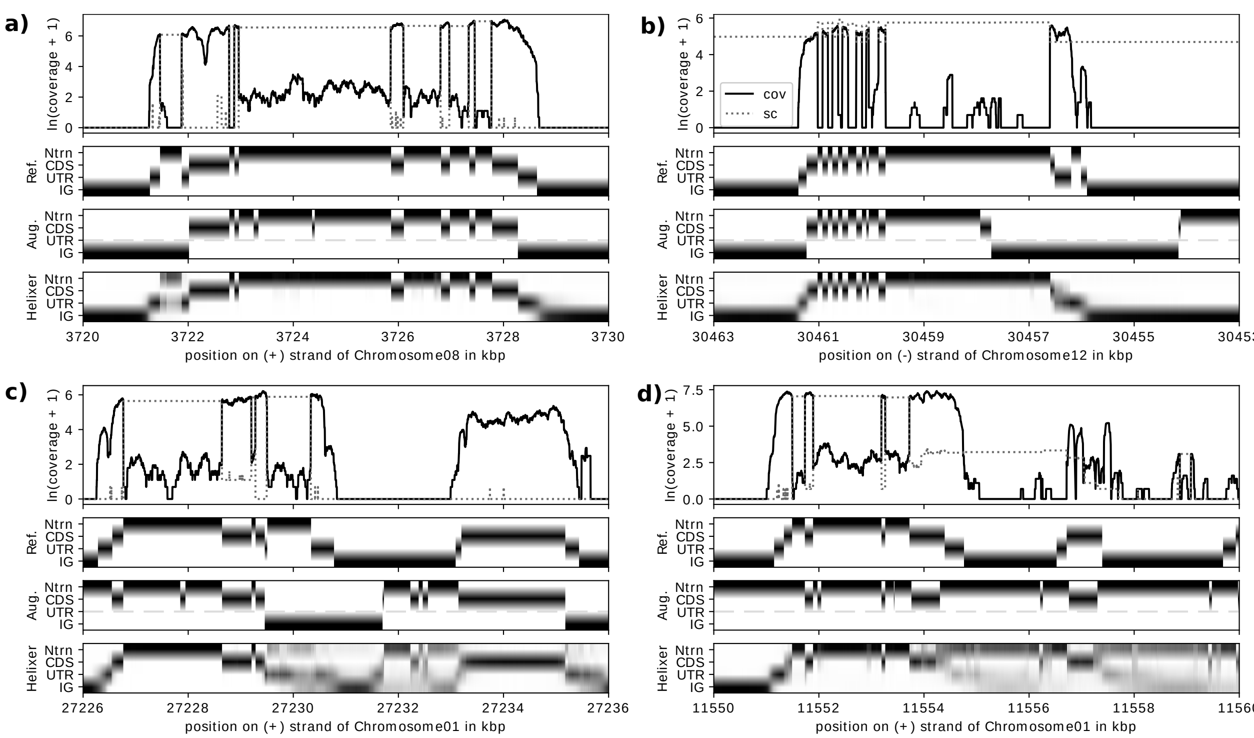

Figure S12: Four additional example helixer predictions in the context of RNAseq data, the reference, and Augustus' prediction for *M. esculenta*. The examples were chosen so that a) Helixer had high accuracy against the reference and the reference was supported by the RNAseq data, b) Helixer had high accuracy but the reference was not supported by the RNAseq data, c) Helixer had low accuracy and the reference was supported by the RNAseq data, and d) helixer had low accuracy but the reference was not supported by RNAseq data. Feasibility for visualization was a major secondary consideration. Each subplot shows from top to bottom i) the natural log of the coverage ("cov", solid) and spliced coverage ("sc", dotted) + 1, ii) the reference annotation in matrix form, iii) Augustus' predictions in matrix form (the dashed line is a reminder that no UTR predictions are expected), and iv) Helixer's predictions. The reference and Augustus have either 0 (white) or 1 (black) for each base pair and category, while Helixer emits a probability from 0-1 represented via gray-scale. "Ntrn" stand for intron, and "IG" stands for intergenic.

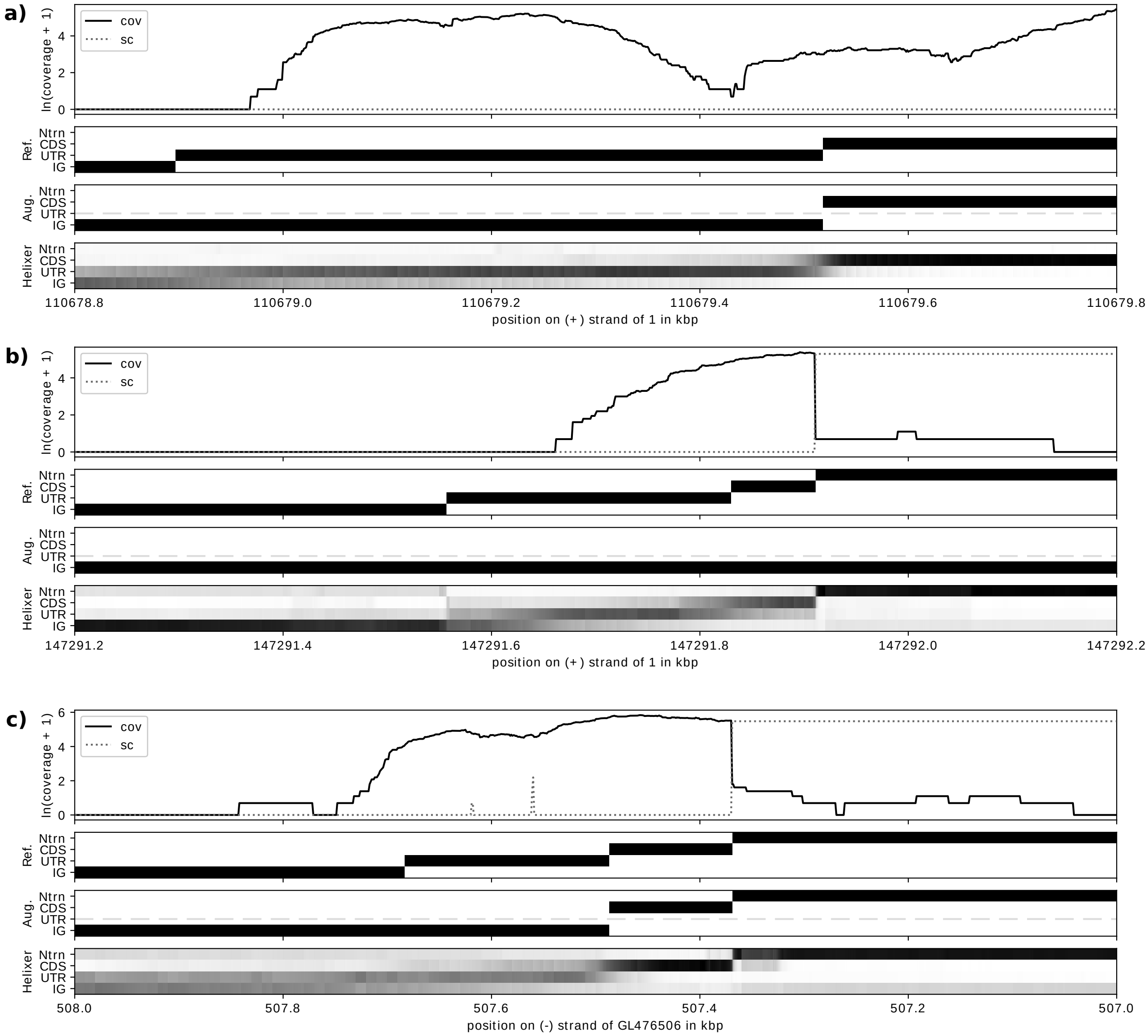

Figure S13: One example of helixer's gradual UTR to intergenic transition predictions from each RNAseq evaluation animal species in the context of RNAseq data, the reference, and Augustus' prediction. The species are a) *P. anubis*, b) *E. caballus* and c) *P. marinus.* Each subplot shows from top to bottom i) the natural log of the coverage ("cov", solid) and spliced coverage ("sc", dotted) + 1, ii) the reference annotation in matrix form, iii) Augustus' predictions in matrix form (the dashed line is a reminder that no UTR predictions are expected), and iv) Helixer's predictions. The reference and Augustus have either 0 (white) or 1 (black) for each base pair and category, while Helixer emits a probability from 0-1 represented via gray-scale. "Ntrn" stand for intron, and "IG" stands for intergenic.

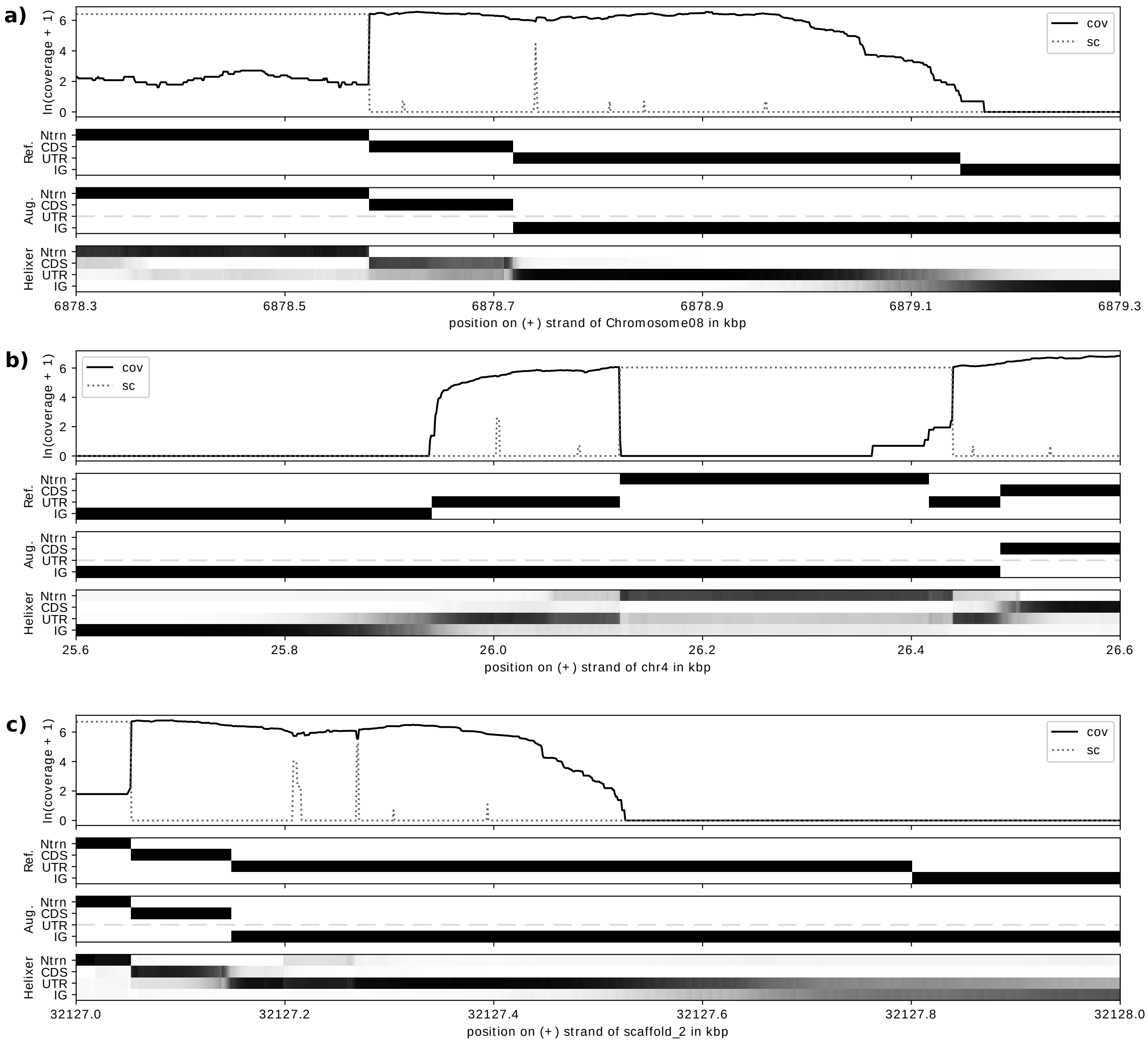

Figure S14: One example of helixer's gradual UTR to intergenic transition predictions from each RNAseq evaluation plant species in the context of RNAseq data, the reference, and Augustus' prediction. The species are a) *M. esculenta*, b) *M. truncatula* and c) *T. cacao*. Each subplot shows from top to bottom i) the natural log of the coverage ("cov", solid) and spliced coverage ("sc", dotted) + 1, ii) the reference annotation in matrix form, iii) Augustus' predictions in matrix form (the dashed line is a reminder that no UTR predictions are expected), and iv) Helixer's predictions. The reference and Augustus have either 0 (white) or 1 (black) for each base pair and category, while Helixer emits a probability from 0-1 represented via gray-scale. "Ntrn" stand for intron, and "IG" stands for intergenic.

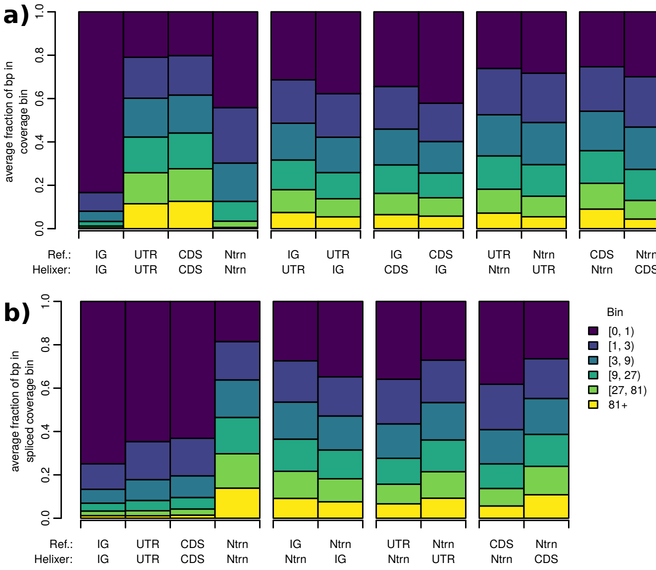

Figure S15: Fraction of bp with color-indicated a) coverage and b) spliced coverage of genomic positions broken down by the confusion matrix of the reference and Helixer's predictions. Categories are only displayed if they can be meaningfully compared by examining a) coverage or b) spliced coverage. The displayed fractions are the averages of the individual fractions for the six RNAseq-evaluation species. The left-most bars show cases where the two tools agree, while the remaining bars show paired conflicts. "Ntrn" stand for intron, and "IG" stands for intergenic.

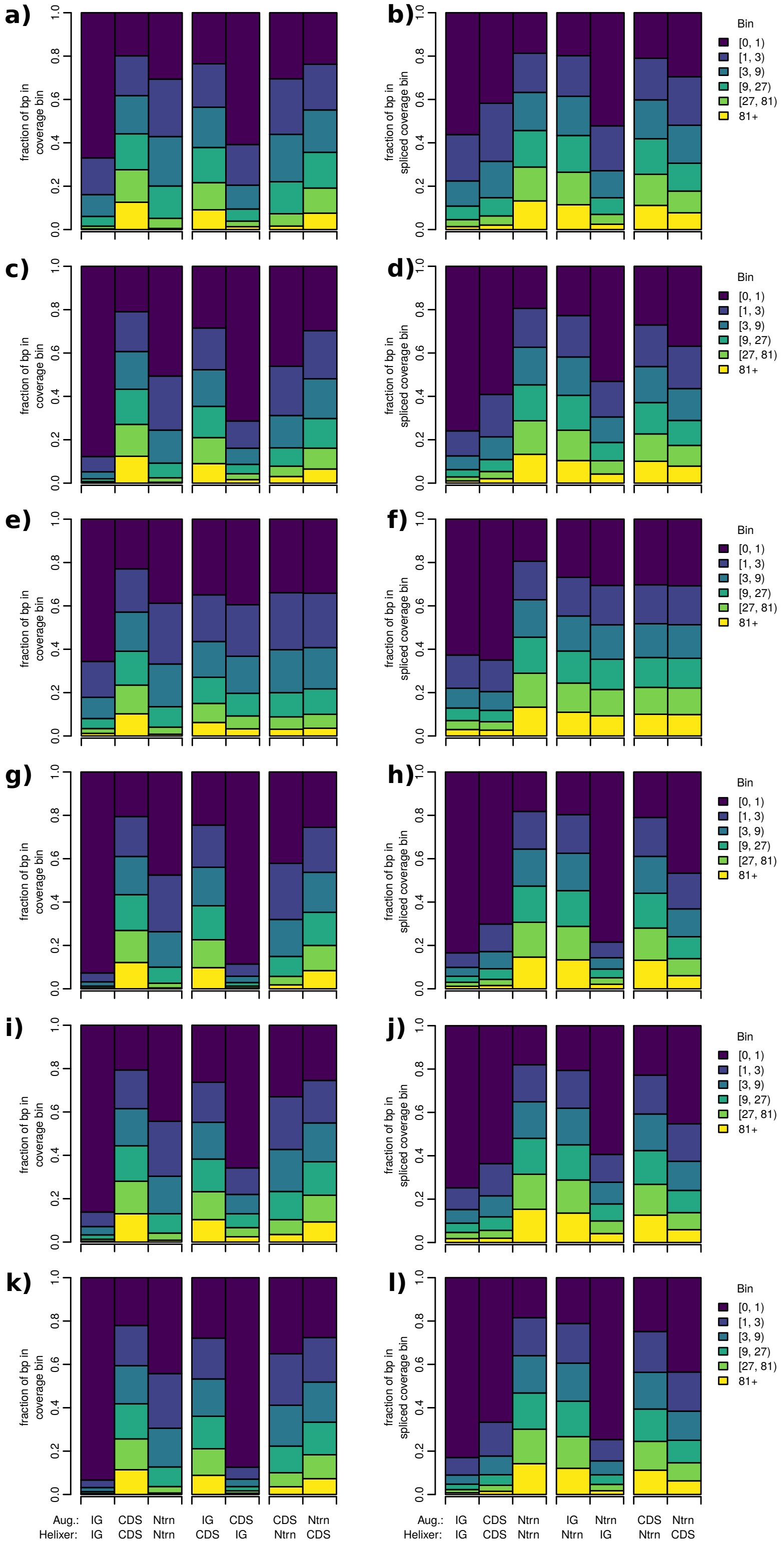

Figure S16: Fraction of bp with color-indicated a, c, e, g, i, k) coverage and b, d, f, h, j, l) spliced coverage of genomic positions broken down by the confusion matrix of Augustus' and Helixer's predictions. Categories are only displayed if they can be meaningfully compared by examining coverage or spliced coverage. The rows show the individual species: a, b) *P. anubis*, c, d) *E. caballus*, e, f) *P. marinus*, g, h) *M. esculenta*, i, j) *M. truncatula*, and k, l) *T. cacao*. The left-most bars show cases where the two tools agree, while the remaining bars show paired conflicts. "Ntrn" stand for intron, and "IG" stands for intergenic.

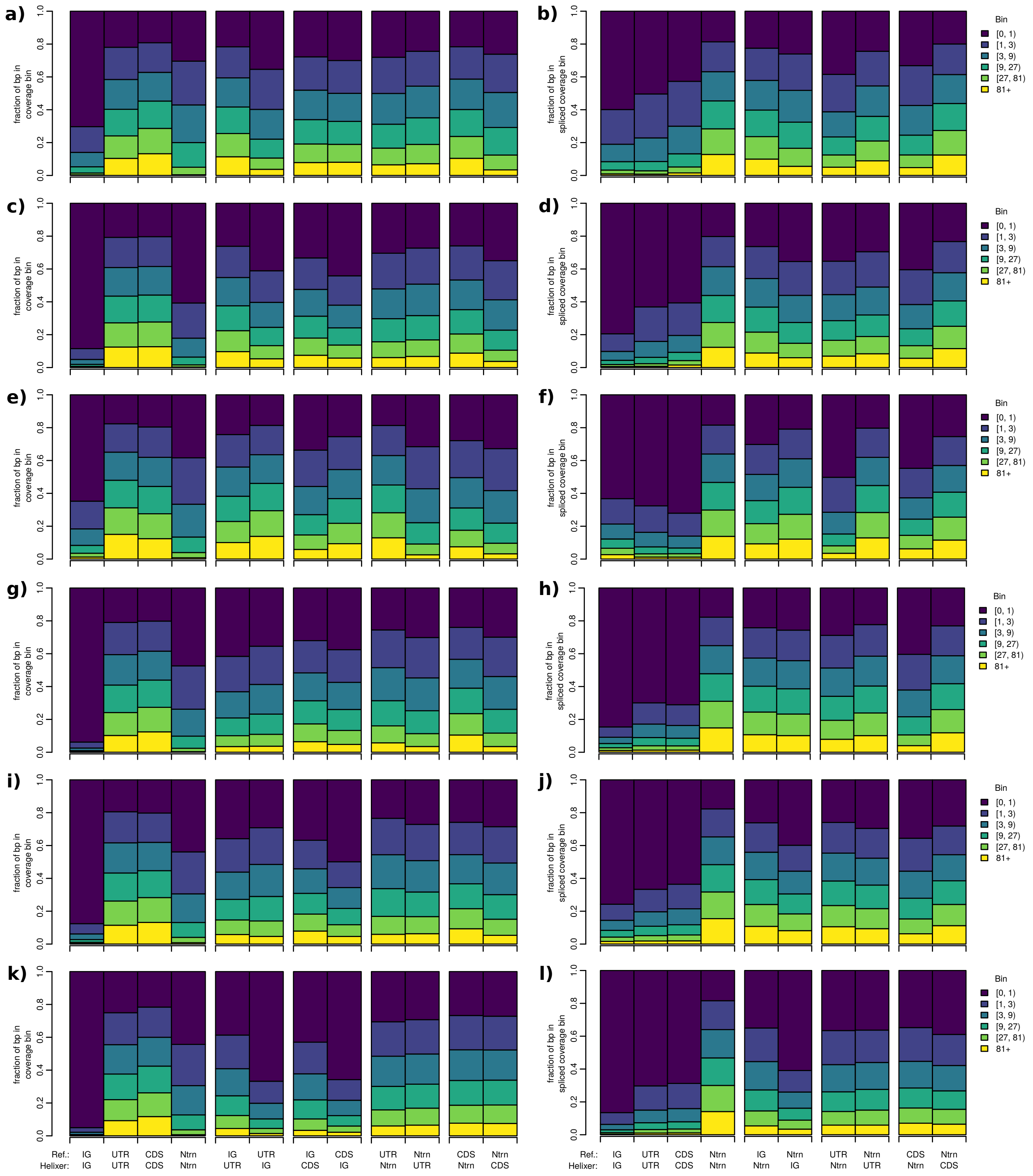

Figure S17: Fraction of bp with color-indicated a, c, e, g, i, k) coverage and b, d, f, h, j, l) spliced coverage of genomic positions broken down by the confusion matrix of the reference and Helixer's predictions. Categories are only displayed if they can be meaningfully compared by examining coverage or spliced coverage. The rows show the individual species: a, b) *P. anubis*, c, d) *E. caballus*, e, f) *P. marinus*, g, h) *M. esculenta*, i, j) *M. truncatula*, and k, l) *T. cacao*. The left-most bars show cases where the two tools agree, while the remaining bars show paired conflicts. "Ntrn" stand for intron, and "IG" stands for intergenic.

10 *In silico* mutagenesis

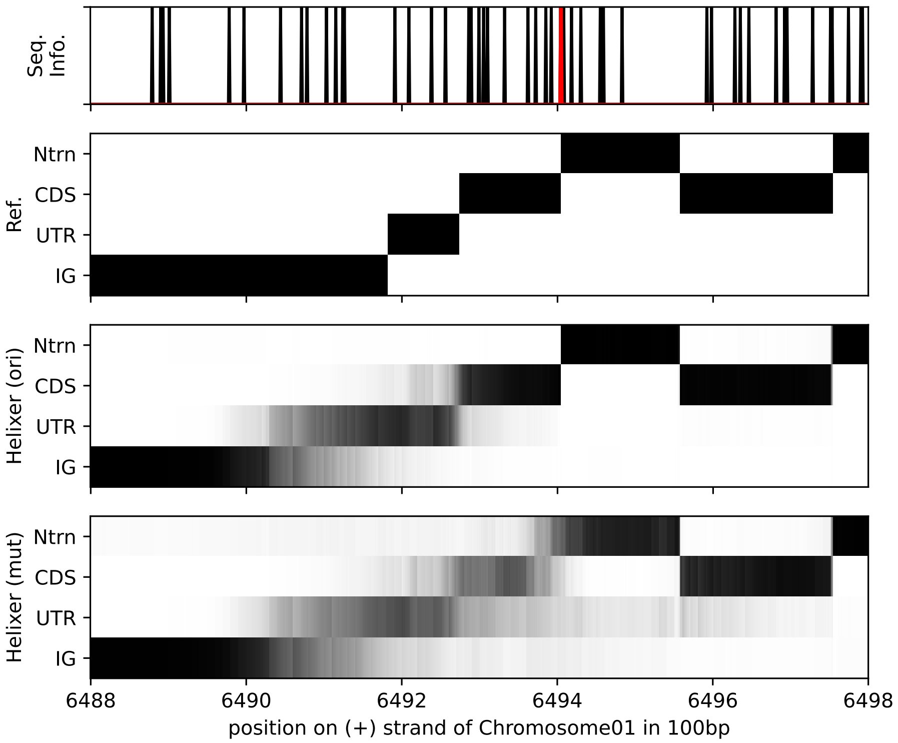

Figure S18: *In silico* mutagenesis of a donor splice site and the effect on Helixer's predictions. Every occurrence of the motif 'gt' is marked with a bar in the top panel. Second from the top is the reference in matrix form, and second from the bottom is Helixer's predictions on the unmodified sequence. On the bottom is Helixer's prediction on a mutated sequence where the 'gt' indicated in red (top panel) has been replaced with 'nn'. The reference has either 0 (white) or 1 (black) for each base pair and category, while Helixer emits a probability from 0-1 represented via gray-scale. "Ntrn" stand for intron, and "IG" stands for intergenic.

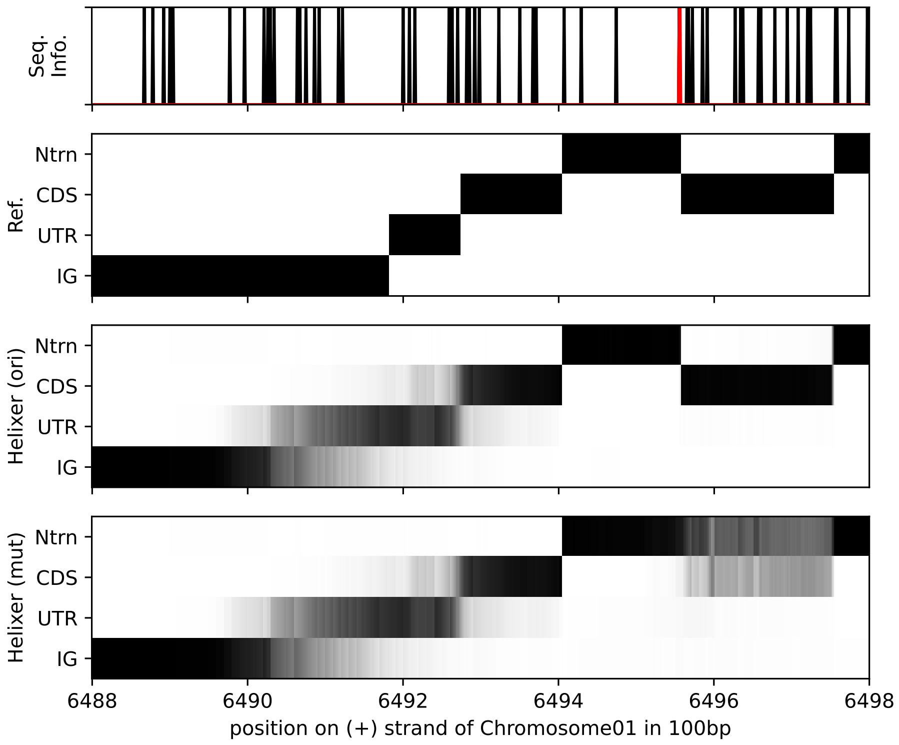

Figure S19: *In silico* mutagenesis of an acceptor splice site and the effect on Helixer's predictions. Every occurrence of the 'ag' is marked with a bar in the top panel. Second from the top is the reference in matrix form, and second from the bottom is Helixer's predictions on the unmodified sequence. On the bottom is Helixer's prediction on a mutated sequence where the 'ag' indicated in red (top panel) has been replaced with 'nn'. The reference has either 0 (white) or 1 (black) for each base pair and category, while Helixer emits a probability from 0-1 represented via gray-scale. "Ntrn" stand for intron, and "IG" stands for intergenic.

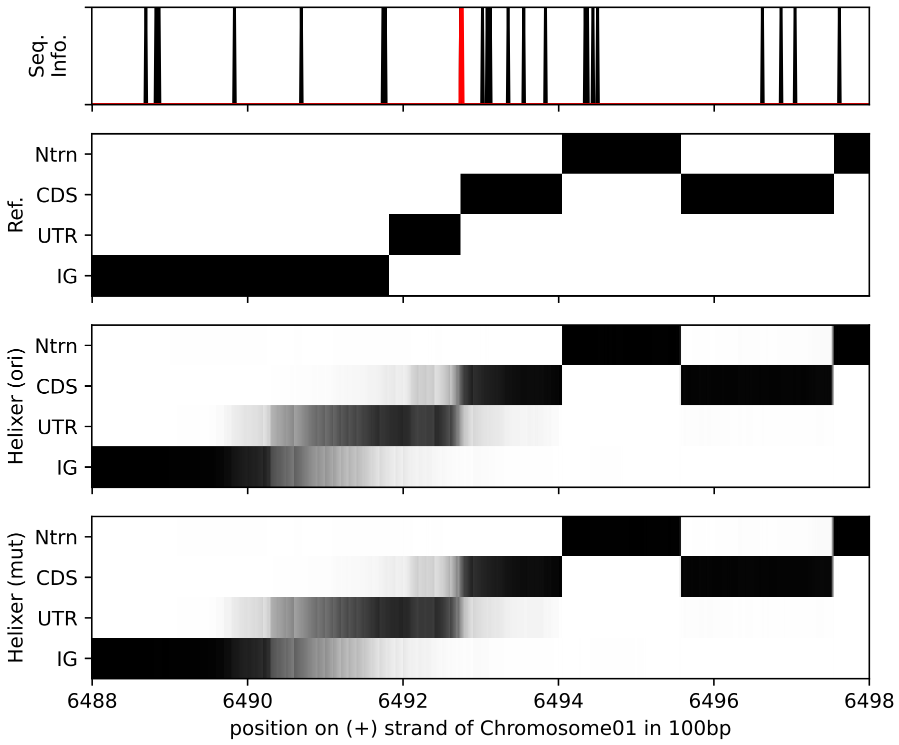

Figure S20: *In silico* mutagenesis of a start codon and the effect on Helixer's predictions. Every occurrence of the motif 'atg' is marked with a bar in the top panel. Second from the top is the reference in matrix form, and second from the bottom is Helixer's predictions on the unmodified sequence. On the bottom is Helixer's prediction on a mutated sequence where the 'atg' indicated in red (top panel) has been replaced with 'nnn'. The reference has either 0 (white) or 1 (black) for each base pair and category, while Helixer emits a probability from 0-1 represented via gray-scale. "Ntrn" stand for intron, and "IG" stands for intergenic.

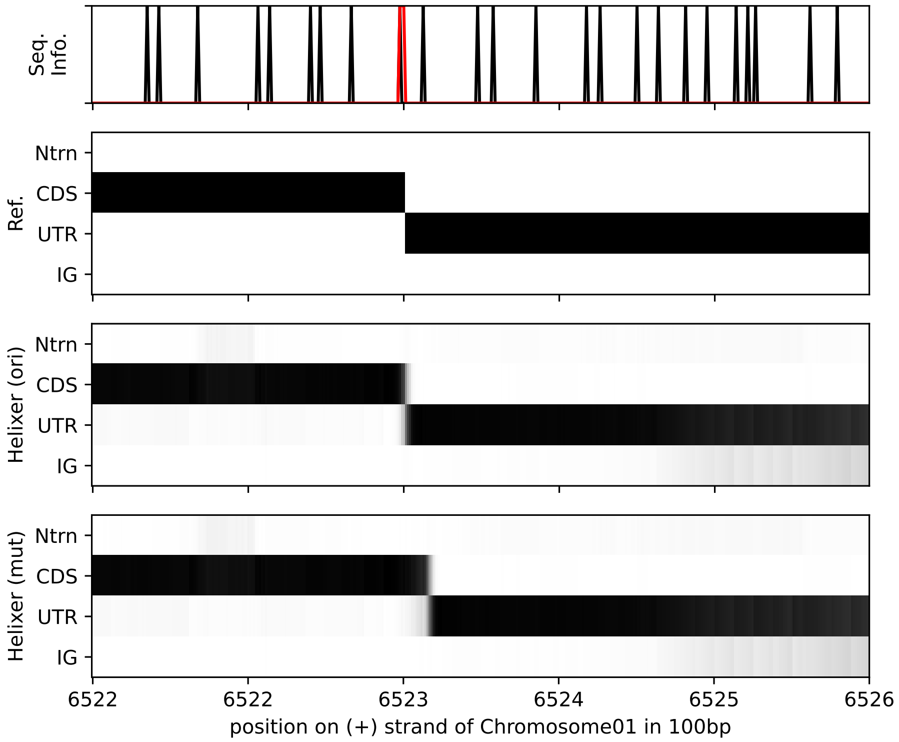

Figure S21: *In silico* mutagenesis of a stop codon and the effect on Helixer's predictions. Every occurrence of the motif 'taa', 'tga' or 'tag' is marked with a bar in the top panel. Second from the top is the reference in matrix form, and second from the bottom is Helixer's predictions on the unmodified sequence. On the bottom is Helixer's prediction on a mutated sequence where the stop codon indicated in red (top panel) has been replaced with 'nnn'. The reference has either 0 (white) or 1 (black) for each base pair and category, while Helixer emits a probability from 0-1 represented via gray-scale. "Ntrn" stand for intron, and "IG" stands for intergenic.

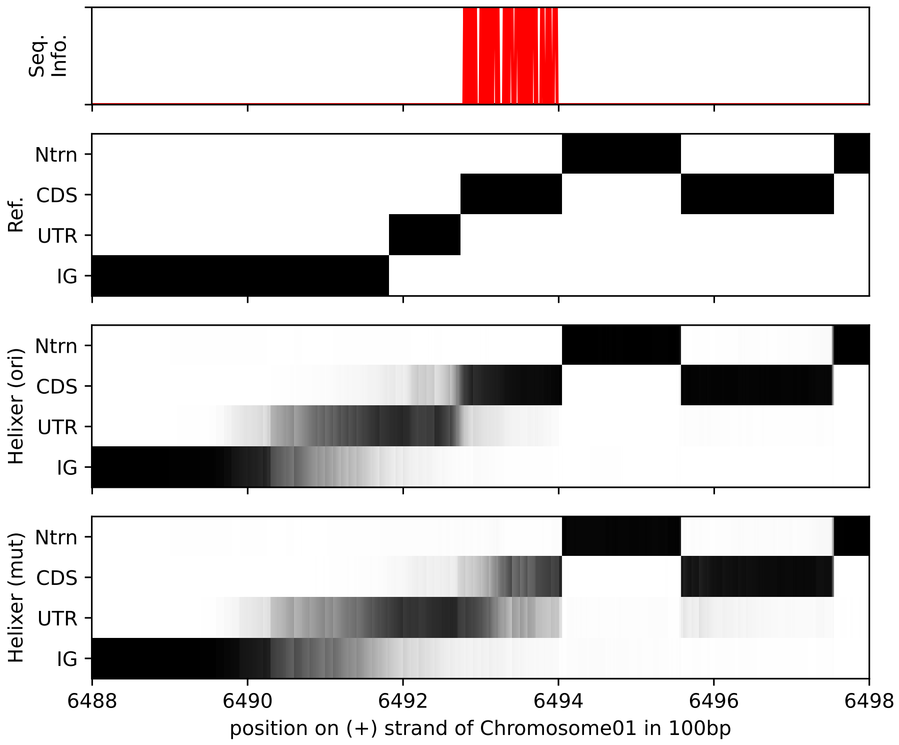

Figure S22: *In silico* mutagenesis to disrupt coding potential and the effect there of on Helixer's predictions. The region marked in red in the top panel has been scrambled in 3bp steps. Second from the top is the reference in matrix form, and second from the bottom is Helixer's predictions on the unmodified sequence. On the bottom is Helixer's prediction on the mutated sequence. The reference has either 0 (white) or 1 (black) for each base pair and category, while Helixer emits a probability from 0-1 represented via gray-scale. "Ntrn" stand for intron, and "IG" stands for intergenic.

Supplemental References

Federhen, S. (2012). The ncbi taxonomy database. *Nucleic acids research*, **40**(D1), D136–D143.

Gupta, A. and Rush, A. M. (2017). Dilated convolutions for modeling long-distance genomic dependencies. *arXiv preprint arXiv:1710.01278*.

Gurevich, A., Saveliev, V., Vyahhi, N., and Tesler, G. (2013). Quast: quality assessment tool for genome assemblies. *Bioinformatics*, **29**(8), 1072–1075.

Marçais, G. and Kingsford, C. (2011). A fast, lock-free approach for efficient parallel counting of occurrences of k-mers. *Bioinformatics*, **27**(6), 764–770.

Quang, D. and Xie, X. (2016). Danq: a hybrid convolutional and recurrent deep neural network for quantifying the function of dna sequences. *Nucleic acids research*, **44**(11), e107–e107.

Simão, F. A., Waterhouse, R. M., Ioannidis, P., Kriventseva, E. V., and Zdobnov, E. M. (2015). Busco: assessing genome assembly and annotation completeness with single-copy orthologs. *Bioinformatics*, **31**(19), 3210–3212.

Trapnell, C., Roberts, A., Goff, L., Pertea, G., Kim, D., Kelley, D. R., Pimentel, H., Salzberg, S. L., Rinn, J. L., and Pachter, L. (2012). Differential gene and transcript expression analysis of rna-seq experiments with tophat and cufflinks. *Nature protocols*, **7**(3), 562–578.
